# Antimony sulfide photovoltaics with high open-circuit voltage not limited by self-trapped excitons

**DOI:** 10.1038/s41467-026-76845-1

**Published:** 2026-08-19

**Authors:** Jiacheng Zhou, Xinwei Wang, Tianle Shi, Lei Wan, Junzhi Ye, Zhiqiang Li, Aron Walsh, Robert L. Z. Hoye, Ru Zhou

**Affiliations:** 1 School of Electrical Engineering and Automation, Hefei University of Technology, Hefei, P. R. China; 2 Department of Materials, Imperial College London, London, UK; 3 Inorganic Chemistry Laboratory, Department of Chemistry, University of Oxford, Oxford, UK; 4 National-Local Joint Engineering Laboratory of New Energy Photoelectric Devices, College of Physics Science and Technology, Hebei University, Baoding, P. R. China

**Keywords:** Electronic devices, Electronic materials

## Abstract

Sb_2_S_3_ is a promising material for low-toxicity, high-stability next-generation photovoltaics, but its device performance is constrained by large open-circuit voltage (*V*_OC_) deficits. From recent spectroscopic investigations, it was hypothesized that this arises from self-trapping, limiting *V*_OC_s to approximately 800 mV, which is indeed the level nearly all Sb_2_S_3_ solar cells have asymptotically approached. Herein, it is revealed through temperature-dependent mobility measurements that band-like transport, rather than self-trapping, occurs in Sb_2_S_3_. By lowering the defect density in Sb_2_S_3_ thin films, the 800 mV threshold is surpassed to achieve a *V*_OC_ of 824 mV. This is accomplished by adding citrate ligands to the precursor solution used for chemical bath deposition, lowering the grain boundary density in Sb_2_S_3_ films from 1114 ± 52 nm μm⁻^2^ to 586 ± 11 nm μm⁻^2^. The likely performance-limiting defects in Sb_2_S_3_ are identified to be S vacancies or Sb on S anti-sites by comparing deep level transient spectroscopy measurements with defect calculations. This work addresses the debate in the field around whether Sb_2_S_3_ is limited by defects or self-trapping, showing that it is possible to improve the performance towards the radiative limit through careful defect engineering.

## Abstract (web summary)

Antimony sulfide solar cells offer low toxicity and high stability, but efficiencies are limited by open circuit voltage. Zhou et al. find performance to not be limited by self-trapping, and that this voltage threshold can be surpassed by defect reduction through grain size increases.

## Introduction

Thin film photovoltaics are important alternatives to silicon-based solar cells because of their high performance, lower materials use, compatibility with fabrication into larger-area modules, and lower levelized cost of electricity^1,2^. For example, CdTe and Cu(In,Ga)(S,Se)_2_ (CIGS) have achieved remarkable certified power conversion efficiencies (PCEs) of 23.1% and 23.6% at the laboratory scale, respectively^3^. Nevertheless, the use of toxic (i.e., Cd) and scarce (i.e., In, Ga) elements impedes their widespread deployment at the multi-terawatt level. The ongoing development of highly efficient, cost-effective, earth-abundant solar cells that have low environmental impact and carbon dioxide equivalent (CO_2_eq) footprint has led to the continuous emergence of light-harvesting materials for next-generation thin film photovoltaics, including Sb_2_S_3_, Sb_2_Se_3_, SnS, GeSe, CuSbS_2_, CuSbSe_2_, Cu_2_ZnSnS_4_, Cu_2_ZnSn(S,Se)_4_^4–12^. Antimony chalcogenides are at the forefront of emerging sustainable photovoltaic materials due to their excellent electronic and optical properties^4,7,13–16^. These materials exhibit a high absorption coefficient (>10^5 ^cm^−^^1^ in the visible range), tunable bandgap (1.1–1.7 eV), and excellent thermal and environmental stability. Additionally, the quasi-one-dimensional (quasi-1D) crystal structure that comprises numerous [Sb_4_S_6_]_n_ or [Sb_4_Se_6_]_n_ ribbons enables efficient charge-carrier transport in the out-of-plane direction if the crystal orientation of antimony chalcogenide thin films can be effectively controlled^17,18^. Sb_2_(S,Se)_3_ solar cells have recently exceeded 10% PCE under 1-sun (i.e., AM 1.5 G) illumination, making them competitive in the emerging thin film photovoltaics field^5,7,13,19^.

**Fig. 1:**
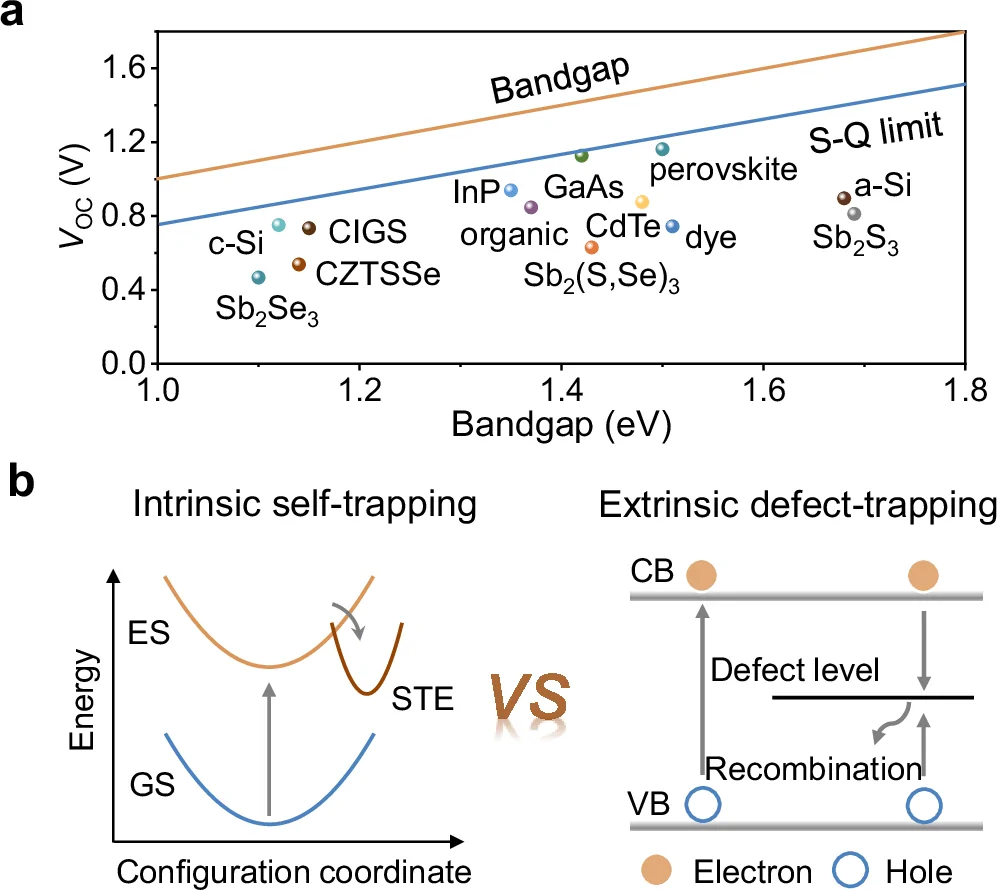
Proposed mechanisms for factors limiting the open-circuit voltage (*V*_OC_) of Sb_2_S_3_ solar cells. **a** Reported *V*_OC_ values of well-developed thin film photovoltaics, as well as the most efficient antimony chalcogenides^1,28^. Theoretical S–Q limit of *V*_OC_ (calculated based on step-function absorptance) as a function of bandgap (solid blue line) is highlighted. **b** Qualitative adiabatic potential energy curve (left) showing the self-trapping effect: photoexcitation from ground state (GS) to excited state (ES) evolves into a self-trapped exciton (STE) state from lattice distortions and loses substantial energy. Schematic (right) showing the defect-trapping effect: the photoexcited electrons and holes suffer from carrier recombination at the defect levels.

The binary compound semiconductor Sb_2_S_3_ possesses a bandgap in the range of 1.7–1.8 eV, close to the optimal value for indoor photovoltaics (IPVs) and top-cells for silicon-based tandem photovoltaics^20–22^. According to the conventional Shockley–Queisser (S–Q) detailed balance limit for the single p-n junction, under standard 1-sun illumination, the theoretical device parameters achievable with a 1.70 eV bandgap light-harvester are an open-circuit voltage (*V*_OC_) of 1.40 V, a short-circuit current density (*J*_SC_) of 22.46 mA cm^−^^2^, a fill factor (FF) of 91%, and a PCE of 28.64%^23,24^. Here, it is worth noting that this standard detailed balance formalism assumes a step-function absorptance at the bandgap and radiative recombination only^24,25^. Over the past decade, Sb_2_S_3_ solar cells have made notable strides in performance. However, the most efficient Sb_2_S_3_ solar cells reported thus far (8.32% PCE) still fall below the conventional S-Q detailed-balance limit^26^. Like other chalcogenide light absorbers, the performance of Sb_2_S_3_ solar cells is primarily limited by the large *V*_OC_ deficit^2^. At present, the highest *V*_OC_ reported so far for Sb_2_S_3_ solar cells is 811.5 mV, and was achieved through in-situ passivation of hydrothermal-processed absorber films^27^; however, most Sb_2_S_3_ devices still show *V*_OC_s in the range of 550–770 mV, much smaller than other photovoltaic devices based on absorbers with similar or smaller bandgaps, such as CdTe, GaAs, CIGS, InP, CH_3_NH_3_PbI_3_ or crystalline-Si, as summarized in Fig. 1a^1,28^. To date, the *V*_OC_ of 753 mV obtained in the highest-efficiency (i.e., 8.32% PCE) Sb_2_S_3_ device is only 53.7% of the conventional S–Q *V*_OC_ limit, suffering from a *V*_OC_ deficit greater than 600 mV^25,26^. The *V*_OC_ deficit is defined as ${V}_{{\mbox{OC}}}^{{\mbox{SQ}}}-{V}_{{\mbox{OC}}}$, where ${V}_{{\mbox{OC}}}^{{\mbox{SQ}}}$ is the S-Q limit *V*_OC_, evaluated using the empirical equation ${V}_{{\mbox{OC}}}^{{\mbox{SQ}}}=0.932\times {E}_{{{{\rm{g}}}}}/q-0.167$ (*E*_g_ is the bandgap of absorber; Fig. 1a)^29^. Overcoming the *V*_OC_ bottleneck is critical to ensure that progress with Sb_2_S_3_ photovoltaics does not stagnate.

The origin of the *V*_OC_ bottleneck for Sb_2_S_3_ solar cells remains under debate, with two perspectives illustrated in Fig. 1b. One hypothesis, proposed by Yang et al., is the self-trapping of photogenerated charge carriers^30^. The proposed mechanism involves excitation from the ground state (GS) to the excited state (ES), before relaxing to self-trapped exciton (STE) states due to electron-phonon coupling, leading to energy losses and reductions in the quasi-Fermi level splitting (Fig. 1b, left). This mechanism is linked to the experimental observation of linearly polarized photoluminescence (PL) that was 600 meV red-shifted compared to the optical bandgap of Sb_2_S_3_^30^. These features were attributed to emission from STE states, and from this data, it was hypothesized that the maximum *V*_OC_ would be 600 mV below the radiative limit of 1.4 V for a 1.7 eV bandgap semiconductor; that is, the maximum *V*_OC_ would be only 800 mV for Sb_2_S_3_ if this hypothesis were true^30^. Similar observations were made for Sb_2_Se_3_, where it was also proposed that self-trapping fundamentally limits the *V*_OC_^31^. Given that self-trapping is an intrinsic process, materials engineering to lower defect densities would not allow the *V*_OC_ and efficiency to exceed the limits set by this process, thus inhibiting future progress with these materials. Indeed, a *V*_OC_ of 800 mV is the value most Sb_2_S_3_ photovoltaics have asymptotically approached over the past decade of efforts made with this material (Supplementary Table 1). In contrast to this hypothesis, first-principles analysis of electron-lattice interactions and charge-carrier mobility suggests that the low effective masses in Sb_2_S_3_ lead to band-like transport in these materials, and the key limiting factor for *V*_OC_ is defect-mediated rather than self-trapping^32^. A deep level residing in the semiconductor bandgap between the conduction band (CB) and valence band (VB) provides an intermediate state to accelerate the carrier recombination process, where excess energy is dissipated as phonons (Fig. 1b, right). Several experimental studies have linked the high *V*_OC_ deficit in Sb_2_S_3_ solar cells to a complex set of defects with deep levels^29,33,34^. For example, we previously showed that lowering the defect density in Sb_2_S_3_ by reducing the grain boundary (GB) density results in an increase in device performance, including improvements in *V*_OC_^14^. Moreover, the justification for self-trapping and *V*_OC_ loss hypothesized by Yang et al. was based on the observed large red-shift in PL compared to the optical bandgap; however, through a careful literature analysis based on reliable reports of PL from Sb_2_S_3_ prepared by a variety of processing methods, the red-shift of PL for Sb_2_S_3_ was found to be only 0–0.1 eV in nearly all other reports (Supplementary Table 2). This further motivates the re-examination of this self-trapping hypothesis in detail. Therefore, in-depth investigations combining theory and experiments are needed to resolve this debate to understand whether Sb_2_S_3_ is fundamentally limited in performance, or has hope still of further improvements towards the radiative limit.

In this work, we developed Sb_2_S_3_ solar cells with *V*_OC_ exceeding the 800 mV threshold by using a complexing agent-mediated chemical bath deposition (CBD) method, which enables the deposition of high-quality Sb_2_S_3_ thin films with GB density approximately halved. The reduced impact of defects for Sb_2_S_3_ films, confirmed by deep-level transient spectroscopy (DLTS) characterization and first-principles calculations, is revealed to contribute to the smaller *V*_OC_ deficit. The obtained *V*_OC_ of 824 mV is the current record for antimony chalcogenide solar cells. Computations further indicate that the *V*_OC_ of Sb_2_S_3_ solar cells can exceed 800 mV by mitigating the effects of the main killer defects in this material, which we found to be sulfur vacancies and antimony on sulfur anti-sites. This work reveals that defect-mediated recombination is a key limitation to the device *V*_OC_ and PCE, and the low performance of Sb_2_S_3_ photovoltaics is surmountable through defect engineering to reach towards the radiative limit.

## Results

### Device performance of planar Sb2S3 solar cells

**Fig. 2:**
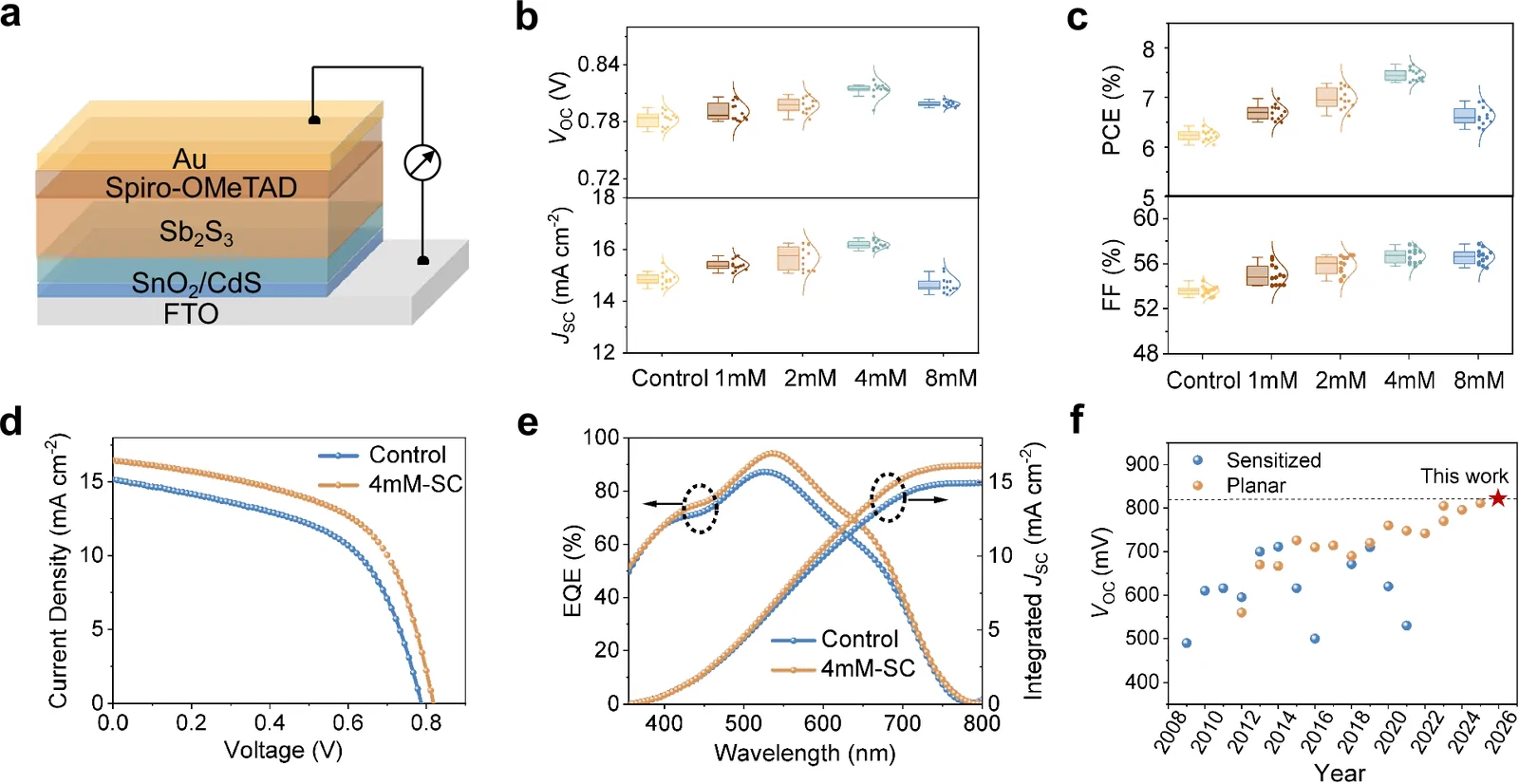
Photovoltaic performance of planar Sb_2_S_3_ thin film solar cells. **a** Schematic of the planar FTO/SnO_2_/CdS/Sb_2_S_3_/Spiro-OMeTAD/Au device stack used in this work. **b**, **c** Distribution of the performance parameters of Sb_2_S_3_ solar cells obtained without (i.e., control) and with the addition of different concentrations of sodium citrate (SC) additives to the precursor solution. Twelve devices were measured for each type of device, and the performance metrics of each device are shown as individual data points. The statistical distributions for the PCE, *V*_OC_, *J*_SC_, and FF were obtained from 12 devices, and the lower whisker, lower box edge, middle line, upper box edge, and upper whisker refer to the minimum, 25th percentile, median, 75th percentile, and maximum of the dataset, respectively. **d** Current density–voltage (*J–V*) curves of best-performing control and 4mM-SC Sb_2_S_3_ solar cells, measured under 1-sun (AM 1.5 G, 100 mW cm^−^^2^) illumination. **e** External quantum efficiency (EQE) spectra of control and 4mM-SC Sb_2_S_3_ solar cells. **f** Evolution in the record *V*_OC_ values of planar and sensitized Sb_2_S_3_ solar cells.

In this work, a modified CBD method was explored to deposit large-grained Sb_2_S_3_ absorber films. It is known that additive engineering in CBD solutions by incorporating reasonable complexing agents is an effective strategy to regulate the film properties (including surface morphology, GB density, defect density, and carrier concentration) via the facile modulation of in-situ chemical environment for film deposition^23,35^. Here, antimony potassium tartrate (C_4_H_4_KO_7_Sb·0.5H_2_O, APT) and sodium thiosulfate (Na_2_S_2_O_3_·5H_2_O, STS) were employed as the Sb and S sources, respectively, and a small amount of sodium citrate (SC) was used as a complexing additive in the precursor solution to manipulate the nucleation and growth kinetics of Sb_2_S_3_ films. An interpretation of the deposition mechanism for CBD in the presence of SC is detailed in Supplementary Note 1. For convenience, the Sb_2_S_3_ samples obtained with the SC concentrations of 1 mM, 2 mM, 4 mM, and 8 mM in the precursor solutions are labeled as 1mM-SC, 2mM-SC, 4mM-SC, and 8mM-SC, respectively. The planar n-i-p device configuration used is FTO/SnO_2_/CdS/Sb_2_S_3_/Spiro-OMeTAD/Au (Fig. 2a). Here, Sb_2_S_3_ is the absorber, and SnO_2_/CdS and Spiro-OMeTAD act as the electron transport layer (ETL) and hole transport layer (HTL), respectively. The fabrication processes of Sb_2_S_3_ solar cells are illustrated in Supplementary Fig. 1 and detailed in Methods. The photovoltaic parameters of Sb_2_S_3_ solar cells, each based on 12 cells, including *V*_OC_, *J*_SC_, FF, and PCE, achieved under 1-sun illumination (AM1.5 G, 100 mW cm⁻^2^), are summarized in the box plots of Fig. 2b, c and Table 1. Here, all devices were obtained with 8 h of CBD of the Sb_2_S_3_ films. As shown, these devices exhibit excellent performance and repeatability. The device PCE increases at first and then decreases with the increase of the SC concentration in the CBD precursor solution. The control Sb_2_S_3_ devices obtained without the use of SC deliver an average PCE of 6.23%, whereas the average PCE is increased to 7.46% for the 4mM-SC devices. The current density–voltage (*J-V*) curves of the best-performing control and 4mM-SC Sb_2_S_3_ solar cells are given in Fig. 2d. The control device yields a *V*_OC_ of 787 mV and a PCE of 6.42%; the 4mM-SC device affords a *V*_OC_ of 817 mV and a PCE of 7.67%, with a champion *V*_OC_ of 824 mV (Table 1). That is, the incorporation of 4 mM SC leads to the relative PCE increasing by nearly 20% compared to the control device. The external quantum efficiency (EQE) spectra of the control and 4mM-SC solar cells, as given in Fig. 2e, reveal that both devices present broad light responses ranging from 350 to 750 nm wavelength. Compared to the control device, the 4mM-SC device exhibits enhanced EQE, exceeding 90% at around 530 nm wavelength. The integrated *J*_SC_ obtained from the EQE spectra are 14.95 and 16.13 mA cm^−^^2^ for the control and 4mM-SC devices, respectively, consistent with the *J*_SC_ measured from *J–V* curves (within 5% deviation). Figure 2f summarizes the *V*_OC_ evolution of well-developed planar and sensitized Sb_2_S_3_ solar cells (detailed in Supplementary Table 1). As shown, the *V*_OC_ has progressively improved over the past decade, especially for the planar configuration, but has increased slowly, in contrast to the rapid increase in PCE. To the best of our knowledge, the champion *V*_OC_ of 824 mV obtained for the 4mM-SC device is the highest value reported for Sb_2_S_3_ solar cells. Interestingly, this *V*_OC_ value is beyond the threshold predicted for Sb_2_S_3_ if it had self-trapping^30^. We also found that CBD-processed Sb_2_S_3_ photovoltaics exhibited excellent environmental stability. The unencapsulated 4mM-SC Sb_2_S_3_ solar cell retained 97.4% of its initial efficiency after 30 days of storage in the dark under ambient temperature in a cabinet with 15% relative humidity (Supplementary Fig. 2).

**Table 1:** Photovoltaic parameters of the control and SC-Sb_2_S_3_ solar cells

Device	*V*_OC_ (mV)	*J*_SC_ (mA cm⁻^2^)	FF (%)	PCE (%)
Control	782 ± 8 (795)	14.87 ± 0.20 (15.15)	53.60 ± 0.42 (53.90)	6.23 ± 0.11 (6.42)
1mM-SC	791 ± 10 (806)	15.40 ± 0.21 (15.76)	55.02 ± 0.90 (56.58)	6.70 ± 0.15(6.98)
2mM-SC	797 ± 8 (807)	15.69 ± 0.45 (16.23)	55.85 ± 0.86 (56.75)	6.98 ± 0.21 (7.29)
4mM-SC	814 ± 8 (824)	16.18 ± 0.15 (16.44)	56.68 ± 0.67 (57.70)	7.46 ± 0.12 (7.67)
8mM-SC	799 ± 3 (803)	14.65 ± 0.31 (15.26)	56.60 ± 0.67 (56.99)	6.62 ± 0.19 (6.93)

Devices measured under 1-sun (AM 1.5 G, 100 mW cm⁻^2^) illumination. Format: average ± standard deviation (best value).

Moreover, it is known that the bandgap of Sb_2_S_3_ is ideal for IPVs^35^. Thus, we further measured the IPV performance of Sb_2_S_3_ solar cells under illumination from white light-emitting diodes (WLEDs) with a correlated color temperature (CCT) of 3000 K. Details of the spectral irradiance, temporal stability of the light source, and IPV performance for the 4 mM-SC device are shown in Supplementary Figs. 3 and 4, along with Supplementary Table 3. The *J*_SC_ is approximately linearly proportional to the light intensity, while the *V*_OC_ and FF increase approximately logarithmically with irradiance. As a result, the highest Sb_2_S_3_ IPV efficiency is under 1000 lux, with a state-of-the-art indoor PCE of 18.56%. This exceeds our previous report of Sb_2_S_3_ IPVs (17.55% indoor PCE), and is comparable to the current state-of-the-art for not just Sb_2_S_3_, but also other Sb- and Bi-based solar absorbers for IPV^35–37^. The loss in *V*_OC_ from 1000 lux to 200 lux in the 4 mM-SC IPVs is 61 mV, which is a small reduction compared to other established and emerging IPVs^38^.

### Characterization of CBD-processed Sb_2_S_3_ films

**Fig. 3:**
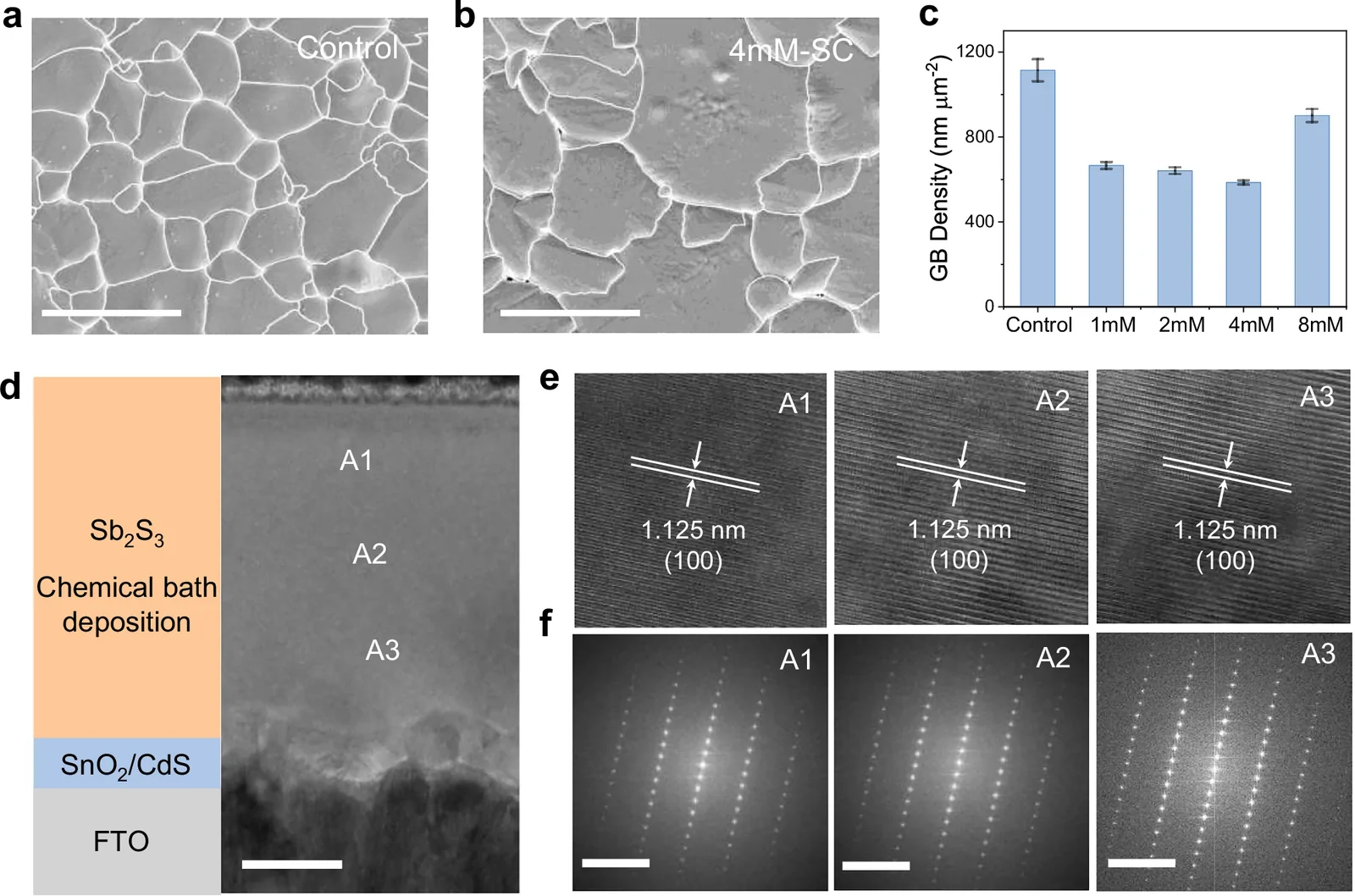
Morphological properties of highly crystalline Sb_2_S_3_ thin films. **a**, **b** Top-view scanning electron microscopy (SEM) images of Sb_2_S_3_ films prepared without (i.e., control) and with the addition of 4 mM SC. The GBs are highlighted to clearly show the changes in GB density. Scale bar, 5 µm. **c** Change in GB density with SC concentration in the precursor solution. Three samples were used to determine the mean GB density values shown, and the error bars denote the standard deviation. **d** Cross-sectional transmission electron microscopy (TEM) image of 4mM-SC Sb_2_S_3_ sample deposited on FTO/SnO_2_/CdS substrate. Scale bar, 100 nm. **e**, **f** High-resolution TEM images performed at points A1, A2, and A3 (see part D), and corresponding fast Fourier transform (FFT) patterns. Scale bar, 5 nm^−1^.

Figure 3a, b shows top-view scanning electron microscopy (SEM) images of Sb_2_S_3_ thin films prepared without the SC additive (i.e., the control sample) and with 4 mM SC in the precursor solution, respectively. It can be seen that the control Sb_2_S_3_ film sample displays grain sizes smaller than 3 μm based on the long diameters of polygon-shaped grains, consistent with that reported for CBD-processed Sb_2_S_3_ films in the literature^4^. With the addition of 4 mM SC, the grain size of Sb_2_S_3_ films involves an obvious increase, with many Sb_2_S_3_ grains exceeding 5 μm. Top-view SEM images of Sb_2_S_3_ films prepared with the use of other SC concentrations are provided in Supplementary Fig. 5. The increase in grain size is accompanied by a decrease in GB density, which is defined as the GB length per unit area. Figure 3c gives the dependence of GB density on the SC concentration for different Sb_2_S_3_ films. As shown, the GB density on the surface of Sb_2_S_3_ films decreases from 1114 ± 52 nm μm^−^^2^ for the control sample to 666 ± 17, 641 ± 16, 586 ± 11, and 901 ± 31 nm μm^−^^2^ for 1mM-SC, 2mM-SC, 4mM-SC, and 8mM-SC film samples, respectively.

To further confirm the single-crystalline nature of individual Sb_2_S_3_ grains observed from SEM images, we performed characterization based on the Sb_2_S_3_ lamella sample (4mM-SC film) prepared by focused ion beam (FIB). The cross-sectional transmission electron microscopy (TEM) image (Fig. 3d) indicates that the Sb_2_S_3_ sample is compact and composed of large grains spanning the thickness of the Sb_2_S_3_ film. Figure 3e shows the high-resolution TEM (HRTEM) images of three arbitrarily selected points A1, A2, and A3, as labeled in Fig. 3d. As shown, points A1, A2, and A3 share identical crystallographic orientations, revealing that the crystal planes extend from the top to the bottom of the Sb_2_S_3_ film. Lattice fringes with interplanar spacings of 1.125 nm, corresponding to the (100) plane in orthorhombic Sb_2_S_3_, were identified^14^. The nearly identical fast Fourier transform (FFT) patterns of HRTEM images of points A1, A2, and A3 (Fig. 3f) further confirm that the individual microstructural units bounded by GBs are indeed single crystals. The cross-sectional elemental mapping images (Supplementary Fig. 6) indicate the uniform distribution of Sb and S across the absorber layer and the conformal growth of Sb_2_S_3_ on top of the CdS buffer layer. The HRTEM image at the CdS/Sb_2_S_3_ interface (Supplementary Fig. 7) reveals the lattice fringes assigned to Sb_2_S_3_ and CdS^14^. The results confirmed that Sb_2_S_3_ films with large single-crystalline grains could be easily produced via CBD.

X-ray diffraction (XRD) patterns of Sb_2_S_3_ films (Supplementary Fig. 8a) show that the diffraction peaks can be well indexed to orthorhombic Sb_2_S_3_ (JCPDS #42-1393)^23^. The diffraction peaks of Sb_2_S_3_ films reveal negligible shifts with the use of SC, indicating that the SC additive could not be incorporated into the Sb_2_S_3_ lattice or interstitial sites. We further calculated the texture coefficient (TC) of the dominant (*hk*0) and (*hk*1) planes to quantify the degree of preferred orientation of Sb_2_S_3_ films (Supplementary Fig. 8b). The addition of SC does not cause significant changes to the film orientation. Additionally, compared to the control film, the SC-Sb_2_S_3_ films involve a gradual reduction in the dominant diffraction peak intensities, such as (120), (130), (211), (221), with the increase in the SC concentration. This should be closely associated with the decrease in the film thickness, as reflected in the cross-sectional SEM images of Sb_2_S_3_ films (Supplementary Fig. 9). As shown, the film thickness gradually decreases from 506 ± 26 nm for the control sample to 394 ± 10 nm, 347 ± 28 nm, 318 ± 15 nm, and 116 ± 32 nm for 1mM-SC, 2mM-SC, 4mM-SC, and 8mM-SC samples, respectively.

Atomic force microscopy (AFM) maps of the morphology of the Sb_2_S_3_ films (Supplementary Fig. 10) confirm that the 4mM-SC film shows larger grain sizes with fewer GBs than the control. Furthermore, the addition of SC leads to a reduction in the root-mean-square roughness from 21.9 nm (control sample) to 16.3 nm (4mM-SC sample). Conductive-atomic force microscopy (c-AFM) images, with the corresponding morphology and current maps (Supplementary Fig. 11) reveal that, compared to the control sample, the 4mM-SC films have more uniform electrical conductivity over the 5 × 5 μm^2^ area measured. We further performed Kelvin probe force microscopy (KPFM) to map the surface potential of Sb_2_S_3_ films (Supplementary Fig. 12), which reveals a higher surface potential for the 4mM-SC film in contrast to the control sample. Given that an increase in the surface potential corresponds to a reduced work function (WF) (i.e., Fermi level closer to vacuum level), the smaller WF for the 4mM-SC sample implies an upshift of its Fermi level^39,40^. This is consistent with the band structure of Sb_2_S_3_ solar cells, obtained from ultraviolet photoelectron spectroscopy (UPS) spectra (Supplementary Fig. 13) and absorption spectra (Supplementary Fig. 14). Compared to the control Sb_2_S_3_ film, the Fermi level of SC-Sb_2_S_3_ is shifted up towards the CB, indicating an increase in the electron carrier concentration for n-type Sb_2_S_3_.

We further investigate the underlying mechanism that could explain the impact of the SC complexing agent on Sb_2_S_3_ film properties. For the CBD of Sb_2_S_3_ films, the substrate is exposed to soluble precursors, which react or decompose to form the desired film materials^41^. From the perspective of nucleation and growth, an intricate balance of growth parameters is necessary to achieve high-quality films. The formation of Sb_2_S_3_ grains typically involves nucleation and growth, which are greatly influenced by the chemical environment of precursor solutions^42–44^. We measured the contact angles of the substrate treated by precursor solutions with different SC concentrations and further analyzed the ratio of the free energies of homogeneous and heterogeneous nucleation as a function of the contact angle (Supplementary Fig. 15a, b, detailed in Supplementary Note 1). The results demonstrate that the incorporation of SC would promote the heterogeneous nucleation of Sb_2_S_3_ on the substrate. Our proposed mechanism is schematically illustrated in Supplementary Fig. 15c. We suggest that adding SC changes the growth mode of Sb_2_S_3_ films from cluster deposition to ion-by-ion deposition. For the pristine CBD solution without the use of SC, the strong complexation effect of APT would cause the formation of many aggregates in the precursor, which serve as homogeneous nucleation centers for Sb_2_S_3_^45^. These small clusters created within the solution further migrate and adhere onto the substrate surface, resulting in the deposition of Sb_2_S_3_ films in the cluster mechanism. For the ion-by-ion deposition mechanism, the complexing agent enables the slow release of free metal ions to directly react with anions onto the substrate in a more controlled manner, being capable of forming well-textured crystalline or even monocrystalline films^41^. Ion-by-ion deposition therefore promotes heterogeneous nucleation on the substrate and suppresses homogeneous nucleation in the precursor solution, similar to the scenario demonstrated for the CBD of other metal chalcogenide thin films, such as ZnS^46^. High-density small nuclei formed on the substrate would further coalesce into a large-grained Sb_2_S_3_ film during the CBD process. Hence, the formation of stable citrate-complexed Sb^3+^ succeeds in controlling the nucleation and growth rate of Sb_2_S_3_, contributing to the deposition of large-grained Sb_2_S_3_ films. In fact, previous studies also demonstrated that films grown by ion-by-ion deposition are usually associated with larger crystalline size compared to those grown by cluster deposition^41^. In addition, the decrease in the release rate of Sb^3+^ further reduces the film thickness, explaining the thickness evolution of Sb_2_S_3_ films with the addition of SC. Apart from the complexation of citrate, we speculate that pH might be another critical factor influencing the deposition of Sb_2_S_3_ films. The influence of pH on the deposition of Sb_2_S_3_ films is further discussed in Supplementary Note 2. It is revealed that, besides the complexation of Sb^3+^ with citrate, an increase in pH should also contribute to a reduction in the deposition rate. However, controlling pH alone does not lead to increases in PCE as high as with the addition of SC (Supplementary Fig. 16). Furthermore, since the incorporation of SC leads to a change in Sb_2_S_3_ film thickness, we also investigated how the performance of Sb_2_S_3_ solar cells depends on absorber thickness (Supplementary Fig. 17). We found that the optimal device PCE of the control sample was still lower than that of the SC-Sb_2_S_3_ samples, confirming that the change in film thickness caused by the use of SC is not the dominant contributor to the efficiency enhancements obtained here. These results imply that the increase in PCE for SC-Sb_2_S_3_ solar cells is mainly associated with the complexation of Sb^3+^ with citrate, which lowers the GB density and reduces the surface roughness of Sb_2_S_3_ absorber films.

### Experimental and theoretical insights into the defect physics of Sb_2_S_3_

**Fig. 4:**
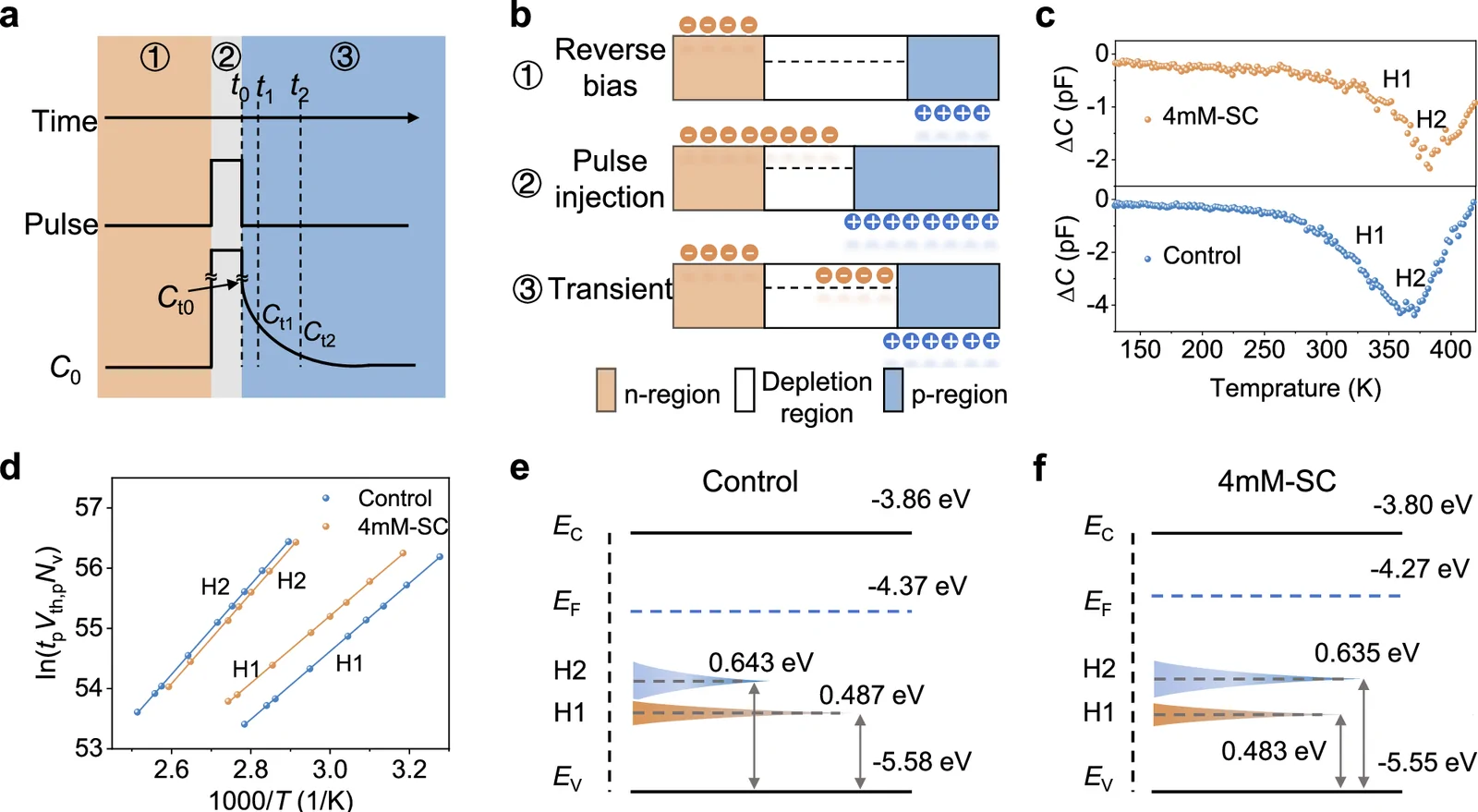
DLTS characterization of defect physics in Sb_2_S_3_ solar cells. **a** Schematic illustration of the mechanism of DLTS measurements. Here, we take the minority traps (i.e., electron traps in p-type semiconductors) as an example to illustrate the change of transient capacitance caused by electron capture and emission. **b** Variation of the depletion width and the process of carriers being trapped and emitted during the measurement. **c** DLTS signals from the control and 4mM-SC Sb_2_S_3_ solar cells. **d** Arrhenius plots derived from DLTS signals. Data points are achieved by calculating internal transients included in DLTS signals with the discrete Laplace transform, and the solid lines are corresponding linear fits. **e**, **f** Schematic of band edge positions and defect levels of the control and 4mM-SC Sb_2_S_3_, respectively, including conduction band minimum (*E*_C_) and valence band maximum (*E*_V_), Fermi level (*E*_F_) and defect levels (H1, H2), relative to the vacuum level.

Understanding the nature of active defects in Sb_2_S_3_ is necessary to design strategies to minimize their impact. DLTS is a powerful technique offering insights into the trap energy, defect type and defect concentration in thin film solar cells^47–50^. DLTS utilizes the transient capacitance of a p-n junction at varying temperatures as a probe to reflect the changes in the charge state of a deep defect center. The traps within the device are filled with carriers by applying a voltage pulse, which in turn alters the capacitance associated with the p-n junction. Figure 4a, b illustrates the mechanism of DLTS measurements. Here, we take the minority traps (i.e., electron traps in p-type semiconductors) as an example to show the change of transient capacitance caused by electron capture and emission. For a p-n junction, when a constant reverse bias (*U*_R_) is applied, a stable space charge region (SCR) is established, corresponding to a stable capacitance (*C*_R_). If an extremely short fill voltage pulse (*U*_P_) is applied as a perturbation, the SCR width and capacitance change due to the injection of majority carriers into the SCR, causing the traps to be filled during this process. Once the voltage pulse is removed, the SCR and capacitance cannot immediately recover to their original state. This delayed recovery occurs because the trapped carriers are emitted to the CB or VB at a specific rate, rather than instantaneously. The capacitance changes (Δ*C* = *C*_t2_ – *C*_t1_) within a time window (*t*_2_ – *t*_1_) *vs*. the different temperatures (*T*) are sampled as the DLTS signals. In particular, the trap type is determined by the Δ*C*. If Δ*C* < 0 and the DLTS signal peaks are negative, they correspond to the minority-carrier traps; otherwise, they would be associated with majority-carrier traps^50–52^. The basic principles of DLTS are detailed in Supplementary Note 3.

As shown in Fig. 4c, the control device and 4mM-device both present two negative DLTS signal peaks in the high-temperature region, denoted as H1 and H2, due to the effect of multiple minority-carrier traps, i.e., hole defects in n-type Sb_2_S_3_ films^6^. The defect characteristic parameters, including trap energy (*E*_T_) and capture cross-section (*σ*), and trap concentration (*N*_T_), can be extracted from the linear fittings of the Arrhenius plots as given in Fig. 4d, with the results summarized in Table 2. Both devices share similar types of deep levels with densities of approximately 10^15 ^cm^−^^3^. The trapping behavior can be quantified by using the thermal trapping rate (*C*_trap_), expressed by ${C}_{{\mbox{trap}}}=1/{\tau }_{{\mbox{trap}}}=v\sigma {N}_{{{{\rm{T}}}}}$^49^, where *τ*_trap_ is the carrier lifetime associated with the trap-assisted Shockley–Read–Hall (SRH) recombination, which is inversely proportional to *σ* ∙ *N*_T_, and *v* is the thermal velocity of charge related to the intrinsic charge transport feature of semiconductors. The values of *σ* ∙ *N*_T_ for the control and 4mM-SC devices are summarized in Table 2. Compared to H1, H2 dominates the charge trapping in absorber films with a larger *σ* ∙ *N*_T_ value and greater energy depth. Compared to the control device, the *σ* ∙ *N*_T_ value for the H2 defect in 4mM-SC Sb_2_S_3_ is decreased. The band edge position and defect levels are illustrated in Fig. 4e, f, with the hole traps situated 0.4–0.7 eV above the valence band maximum (VBM). Since the trap energy reflects the difficulty of charge emission from the traps, the H1 and H2 defects located at more than 0.3 eV above the VBM would trap carriers without re-emission and act as recombination centers due to their high ionization energy, contributing to severe non-radiative recombination. In particular, the deep-level defects would cause a fluctuation in the bandgap and bring band tail states within the bandgap, giving rise to a further *V*_OC_ loss. Hence, the 4mM-SC Sb_2_S_3_ film with reduced defect density is suggested to effectively suppress charge-carrier recombination in solar cells.

**Table 2:** Defect characteristic parameters

Devices	Trap	*E*_T_ (eV)	*σ* (cm^2^)	*N*_T_ (cm^−^^3^)	*σ* ∙ *N*_T_ (cm^−^^1^)
Control	H1	*E*_V_ + 0.487	4.43 × 10^−^^17^	2.42 × 10^15^	0.107
	H2	*E*_V_ + 0.643	7.35 × 10^−^^16^	1.39 × 10^15^	1.020
4mM-SC	H1	*E*_V_ + 0.483	2.08 × 10^−^^17^	9.14 × 10^14^	0.019
	H2	*E*_V_ + 0.635	6.62 × 10^−^^16^	1.03 × 10^15^	0.682

These parameters are the trap energy level (*E*_T_), capture cross-section (*σ*), and concentration (*N*_T_), obtained using DLTS measurements in the control and 4mM-SC Sb_2_S_3_ solar cells.

**Fig. 5:**
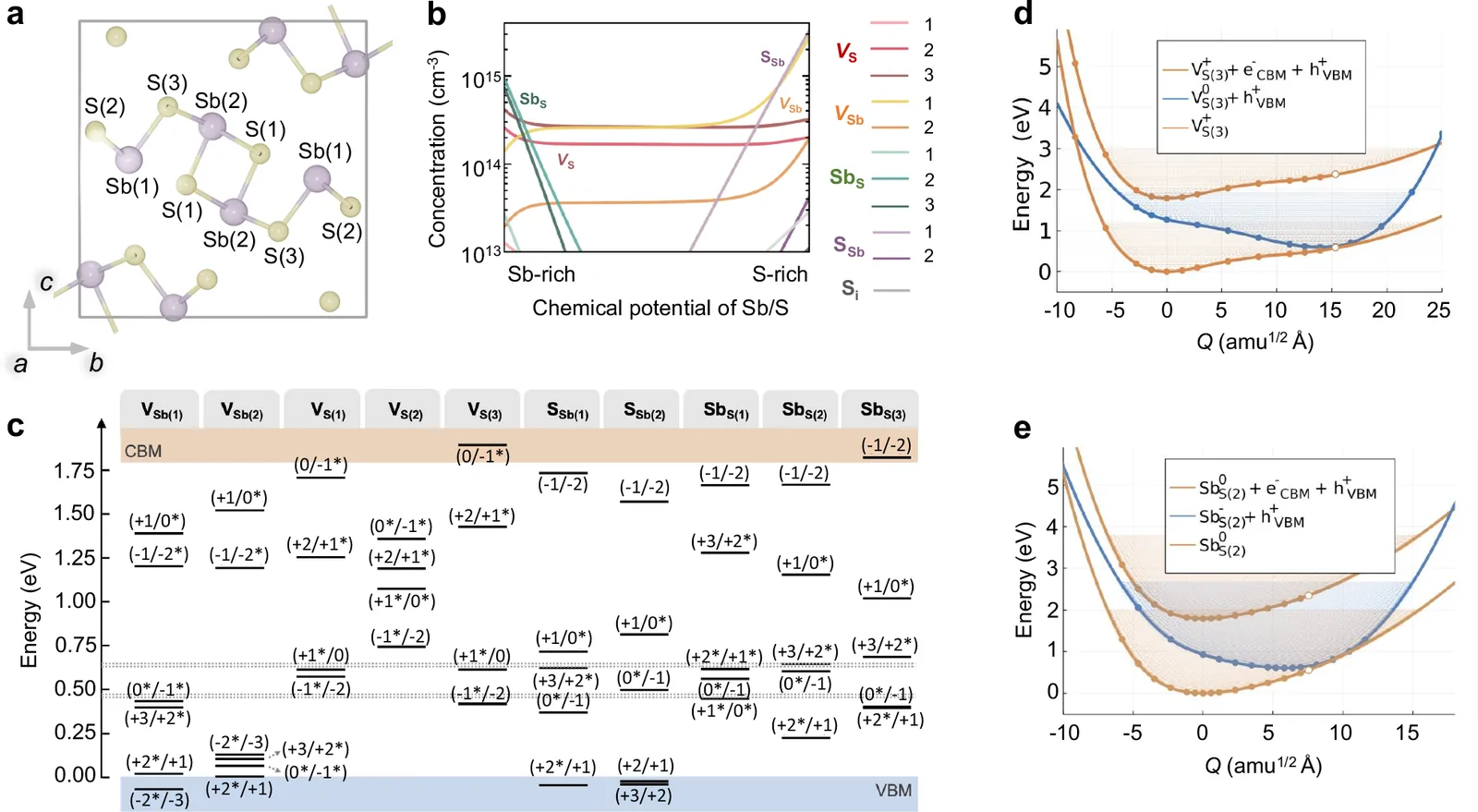
First-principles calculations to identify defect species. **a** Illustration of a [Sb_4_S_6_]_n_ unit in the Sb_2_S_3_ crystal structure. **b** Calculated equilibrium defect concentration at 300 K in Sb_2_S_3_ crystals grown at 643 K as a function of growth conditions based on the defect energies reported in ref. ^56^. The numbers in the legend represent different inequivalent sites. **c** Calculated charge transition levels of intrinsic point defects with high concentrations under Sb-rich conditions in Sb_2_S_3_. Metastable charge states are marked with asterisks (*). The Fermi level is referenced to the valence band maximum (VBM). Dashed gray lines indicate trap levels in control and 4mM-SC Sb_2_S_3_ devices, determined by DLTS. **d**, **e** 1D configuration coordinate diagram for charge transitions between V_S(3)_^+^ and V_S(3)_^0^ as well as Sb_S(2)_^0^ and Sb_S(2)_^−^. *Q* represents the generalized configuration coordinate, which describes the collective atomic displacement along the reaction pathway during charge transition. The equilibrium structures of V_S(3)_^+^ and Sb_S(2)_^0^ are set as references with *Q* = 0 amu^1/2^ Å and total energy *E* = 0 eV. Solid circles are data points obtained by DFT calculations and used for fitting, while hollow circles are discarded for fitting due to charge delocalization. Solid lines represent best fits to the data.

To identify the defect species, we performed first-principles calculations combined with a global searching strategy, implemented in the doped^53^ and ShakeNBreak packages^54^, to systematically explore all symmetry-inequivalent configurations of intrinsic point defects in Sb_2_S_3_. It is known that quasi-1D Sb_2_S_3_ consists of [Sb_4_S_6_]_n_ chains which have five nonequivalent atomic sites, i.e., Sb(1), Sb(2), S(1), S(2) and S(3), as illustrated in Fig. 5a. First principles calculations have shown that Sb_2_S_3_ involves intricate defect types, including five vacancies (V_Sb(1)_, V_Sb(2)_, V_S(1)_, V_S(2)_ and V_S(3)_), five anti-sites (S_Sb(1)_, S_Sb(2)_, Sb_S(1)_, Sb_S(2)_ and Sb_S(3)_), and twelve interstitials. The interstitials were identified using the Voronoi scheme^55^, despite the material being comprised of only two elemental components^56–58^. Our calculation details are provided in Ref. ^56^ and Methods. The equilibrium defect concentrations as a function of the Sb/S chemical potential are shown in Fig. 5b. Under Sb-rich conditions (i.e., synthesis conditions for control and 4mM-SC Sb_2_S_3_ devices), Sb_S_, V_S_, and V_Sb_ are the most dominant point defects with concentrations higher than 10^14 ^cm^−^^3^. The corresponding defect levels are shown in Fig. 5c and summarized in Supplementary Table 4. Except for V_Sb(2)_, most of these defects have traps that match those observed experimentally via DLTS (as indicated by the dashed gray lines). This highlights the challenges in identifying specific defects based solely on defect concentrations or energy levels in Sb_2_S_3_. Moreover, our global search reveals that all intrinsic point defects in Sb_2_S_3_ are amphoteric, exhibiting both donor- and acceptor-like charge states. Therefore, directly comparing experimental defect levels with simulations under the assumption that each defect acts exclusively as a donor or an acceptor may be misleading^57^, since amphoteric defects introduce additional possibilities for matching the measured levels.

While defect formation energies and defect levels calculated from simulations provide insights into the thermodynamic stability, they do not directly account for kinetic factors, such as carrier capture processes under real device conditions. Experimental techniques are sensitive to defects that actively capture charge-carriers. To identify the most relevant defects, we first selected those with defect levels and equilibrium concentrations close to the experimental values (Supplementary Table 5), then investigated the potential energy surfaces associated with charge transition processes. Capture cross-sections were then calculated for each transition using configuration coordinate (CC) diagrams^56^. The magnitude of the cross-sections was used to determine whether each defect predominantly behaves as an electron or a hole trap. Based on the combined information from defect levels, concentrations, and capture cross-sections, we suggest V_S(3)_ and Sb_S(2)_ as the defects that are most consistent with the experimental observations, with calculated trap levels located at 0.59 and 0.61 eV above the VBM and calculated capture cross-sections of 3.77 × 10^−^^16^ and 4.00 × 10^−^^17^ cm^−^^2^, respectively. These calculated levels are closest to the experimentally observed H2 levels, differing by approximately 0.05 eV for V_S(3)_ and 0.03 eV for Sb_S(2)_. Their differences from the H1 levels are larger but remain on the order of 0.1 eV. We note that the calculated transition levels are the thermodynamically stable states at absolute zero temperature, whereas DLTS extracts activation energies from finite temperature carrier emission processes. The small differences between the calculated and experimental trap levels on the order of 0.1 eV or less are consistent with the typical uncertainty in comparing state-of-the-art hybrid functional defect calculations with experimental defect levels^59,60^. We do, however, acknowledge that these deviations prevent us from unambiguously assigning the experimental H1 and H2 levels to V_S(3)_ and Sb_S(2)_. Regardless, this does not affect the conclusion that V_S(3)_ and Sb_S(2)_ are deep hole traps with efficient non-radiative capture.

The CC diagrams for V_S(3)_ and Sb_S(2)_ are shown in Fig. 5d, e. A larger displacement along the generalized coordinate *Q* corresponds to stronger lattice relaxation during the charge transition process. For both defects, the energy barriers for hole capture are very small, and the corresponding phonon wavefunctions of the initial and final states exhibit large overlap. A small capture barrier combined with large phonon overlap greatly enhances the transition probability, leading to large hole capture cross-sections on the order of 10⁻^16^ ~ 10⁻^17^ cm^2^.

We also evaluated the Franck-Condon relaxation energies and defect transition Stokes shifts for these hole capture processes from the CC diagrams (Supplementary Fig. 18, Supplementary Tables 6 and 7, detailed in Supplementary Note 4). The calculated Stokes shifts are 1.26 eV for V_S(3)_ and 0.68 eV for Sb_S(2)_, consistent with large lattice relaxation. Together with the large capture cross-sections, these large Stokes shifts suggest efficient non-radiative multiphonon capture, so any radiative signatures from these deep defect transitions would likely be weak or masked rather than directly observable as PL red-shifts. That is, we would not expect these two point defects to contribute substantially to the PL observed from Sb_2_S_3_ thin films.

Based on the most-probable limiting defects identified (V_S(3)_ and Sb_S(2)_), and their measured trap densities and capture cross-sections, we predicted the trap-limited *V*_OC_ accounting for both radiative (band-to-band) and defect-mediated non-radiative recombination processes (Supplementary Note 5 and Supplementary Fig. 19). It is worth noting that, within the conventional S-Q detailed balance framework, a material-specific radiative reference based on the finite-thickness absorptance of Sb_2_S_3_ is used for the trap-limited *V*_OC_ analysis. The predicted *V*_OC_ reaches 1.15 V for the control sample and 1.26 V for the 4mM-SC sample, both exceeding the 0.8 V threshold. The predicted improvement in performance and *V*_OC_ by lowering trap density for the 4 mM-SC sample is qualitatively consistent with the improvement observed experimentally. At the same time, these predictions show that other loss processes are present, beyond non-radiative losses from V_S(3)_ and Sb_S(2)_.

### Charge-carrier transport properties and device physics

**Fig. 6:**
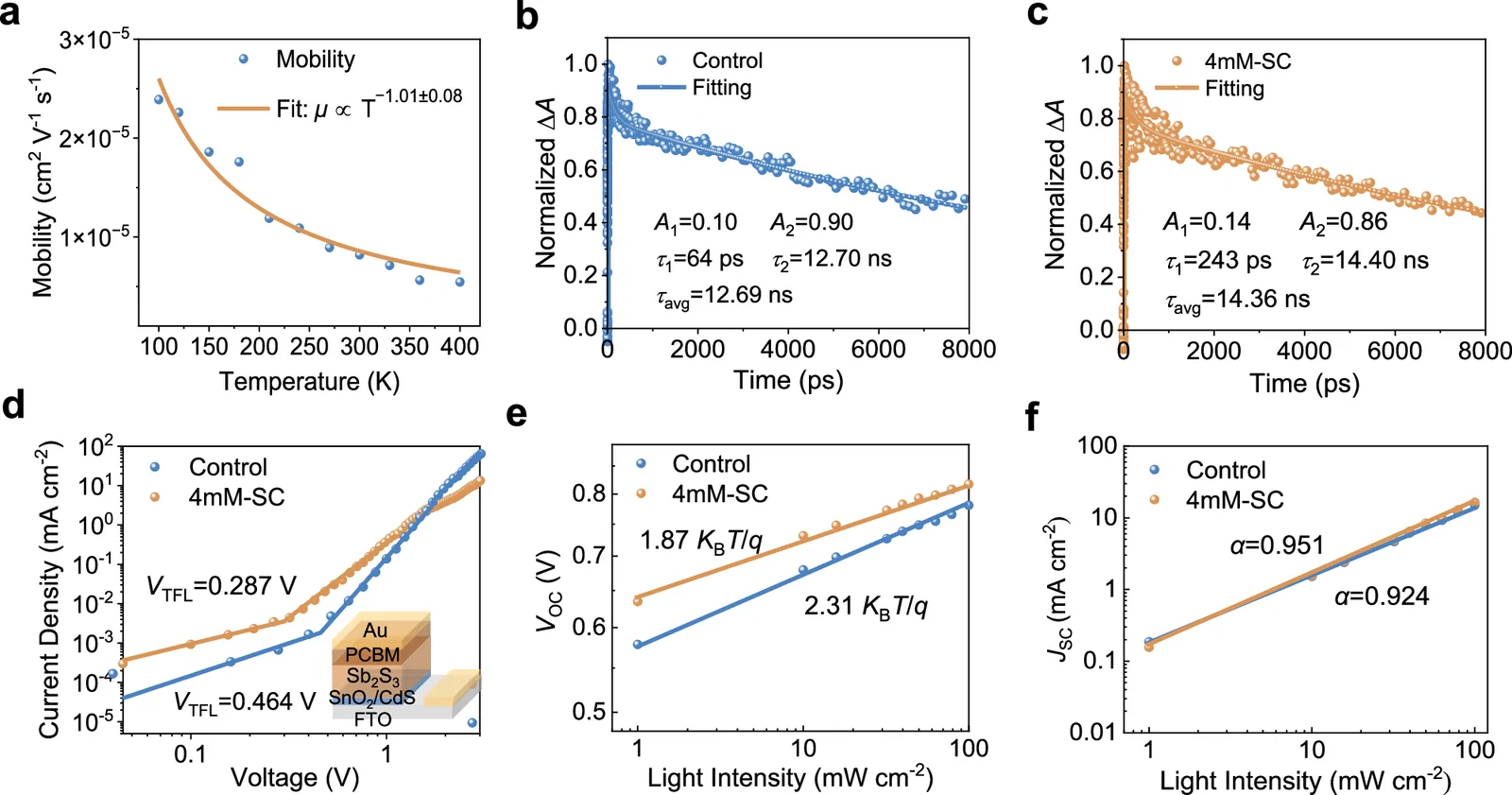
Charge-carrier transport properties and device physics. **a** Temperature-dependent mobility of Sb_2_S_3_ films determined using time of flight (TOF) measurements, fitted with a power law model that indicates $\mu \propto {T}^{-1.01\pm 0.08}$. Here, ±0.08 is the uncertainty of the exponent obtained by fitting the data across all temperatures. **b**, **c** Photoinduced absorption (PIA) kinetics (scatter) measurements monitored at 560 nm from transient absorption (TA) spectroscopy, along with phenomenological biexponential curve fits (solid line) for the TA spectra of the control and 4mM-SC Sb_2_S_3_ films. Excitation was with a 400 nm wavelength pulsed laser at a repetition rate of 1000 Hz. Δ*A* is defined as the change in the absorption of the sample before and after pumping. **d** Space-charge-limited current density (SCLC) measurements of the control and 4mM-SC devices based on the electron-only device, with the configuration: FTO/SnO_2_/CdS/Sb_2_S_3_/PCBM/Au. Here, both devices share thickness-matched absorber films of 318 ± 15 nm to exclude the influence of film thickness on the electrical properties. **e**, **f** Dependences of *V*_OC_ and *J*_SC_ on the light intensity for the control and 4mM-SC Sb_2_S_3_ solar cells.

To verify the formation of delocalized large polarons instead of small polarons in Sb_2_S_3_, we determined the temperature dependence of the mobility through time-of-flight (TOF) measurements^61^. It is known that large polarons show a reduction in the overall mobility with increasing temperature because of increased phonon scattering. On the other hand, if small polarons dominate charge-carrier transport, the mobility should increase as the temperature increases because small polaron formation changes the transport mechanism to hopping-based. The device structure for TOF measurements was FTO/Sb_2_S_3_/Au, and the measurement details can be found in Methods. Note that CBD was unable to grow compact films directly on the FTO substrates, and so we used close-spaced sublimation (CSS) to form continuous thin films that avoid shunting; we would not expect any differences in the trends in mobility with temperature for CBD *vs*. CSS-grown Sb_2_S_3_. Figure 6a shows the obtained mobilities of Sb_2_S_3_ at different temperatures ranging from 100 to 400 K based on the bilogarithmic plots of transient current *vs*. transit time (Supplementary Fig. 20). The observation of an obvious decrease in mobility with an increase in temperature is consistent with the behavior of large polarons. The obtained mobilities are surprisingly as low as 10^−^^5^–10^−^^6^ cm^2^ V^−^^1^ s^−^^1^. Previous studies also demonstrated very low mobilities with similar magnitudes, such as 4.9 × 10^−^^5^ cm^2^ V^−^^1^ s^−^^1^ based on defect-resolved mobility measurements^62^, 2.2 × 10^−^^5^ cm^2^ V^−^^1^ s^−^^1^ based on space-charge-limited current (SCLC)^63^, 3 × 10^−^^3^ cm^2^ V^−^^1^ s^−^^1^ based on time-resolved microwave conductivity (TRMC) measurements^64^. By fitting a power law model to the mobility data, the exponent was found to be −1.01 ± 0.08. However, this exponent does not straightforwardly imply any particular scattering mechanism^65^.

Ultrafast transient absorption (TA) spectroscopy analysis was further performed to understand the charge-carrier kinetics of FTO/SnO_2_/CdS/Sb_2_S_3_ films, where we focused on CBD-grown Sb_2_S_3_, in order to be consistent with our device measurements, and because Sb_2_S_3_ grown by CBD forms continuous films on CdS. The TA spectra of both the control sample and the 4mM-SC sample (Supplementary Fig. 21a, b) display distinct negative differential absorbance (ground state bleach (GSB) peaks), as well as regions of positive differential absorbance (photoinduced absorption (PIA) peaks). The GSB peaks observed at the wavelengths of 460–520 nm and 610–680 nm can be ascribed to the state filling of CdS and the GS absorption of Sb_2_S_3_, respectively. The PIA peaks centered at 520 to 610 nm wavelength can be assigned to the formation of sulfide radicals (${{{{\rm{S}}}}}^{-{{{\rm{\bullet }}}}}$) as a result of the localization of photogenerated holes on the S atom within the Sb_2_S_3_ lattice^6,14,66^. The transient kinetic decays monitored at 560 nm for both the control and 4mM-SC films can be extracted from the pseudocolor images for the TA spectra of both samples (Supplementary Fig. 21c, d), as presented in Fig. 6b, c. The transient dynamics are fit well with a phenomenological biexponential model in order to quantitatively compare between samples. The gradual decrease of the PIA peak can be attributed to the decay of trapped holes, i.e., the ${{{{\rm{S}}}}}^{-{{{\rm{\bullet }}}}}$ species, which we here attribute to non-radiative charge-carrier recombination in Sb_2_S_3_. The 4mM-SC sample delivers higher average time constant (*τ*_avg_) values (14.36 ns) compared to the control sample (12.69 ns). Such lifetime values lie within the range of 4–60 ns that are commonly reported for antimony chalcogenides^33^. In general, the charge-carrier lifetime of the absorber material is inversely related to the reverse-bias saturation current density (*J*_0_) of the device. Hence, a long carrier lifetime is the prerequisite for achieving high *V*_OC_ of solar cells.

We further investigated the device physics of Sb_2_S_3_ solar cells to fully understand the underlying mechanism of the performance enhancements we obtained. The SCLC measurements of the dark currents from the electron-only devices (FTO/SnO_2_/CdS/Sb_2_S_3_/PCBM/Au) are given in Fig. 6d. In the low-voltage Ohmic region, the curve is generally linear. When the voltage exceeds the trap-filling limit, the current increases substantially, implying that the injected carriers have completely occupied the trap states^26^. The trap-filled limiting voltage (*V*_TFL_) values for the control and 4mM-SC device are estimated to be 0.464 V and 0.287 V, respectively. The trap state density (*n*_τ_) of Sb_2_S_3_ films can be evaluated according to the equation ${V}_{{\mbox{TFL}}}=q{n}_{{{{\rm{\tau }}}}}{L}^{2}/2{\varepsilon }_{{{{\rm{r}}}}}{\varepsilon }_{0}$, where *q* is the elementary charge, ${n}_{{{{\rm{\tau }}}}}$ is the trap state density, *L* is the thickness of the absorber film (here the control and 4mM-SC devices share thickness-matched absorber films of 318 nm), ${\varepsilon }_{0}$ is the vacuum permittivity (8.85 × 10^−^^12^ F m^−^^1^), and ${\varepsilon }_{{{{\rm{r}}}}}$ is the relative permittivity of Sb_2_S_3_ (6.67 for the static dielectric constant)^67^. As a result, the trap state densities for the control and 4mM-SC devices are estimated to be 3.39 × 10^15 ^cm^−^^3^ and 2.12 × 10^15 ^cm^−^^3^, respectively. The overall reduction in trap state density for the 4mM-SC Sb_2_S_3_ sample is consistent with the reduction in GB density, and the order of magnitude of the trap density is consistent with DLTS measurements. Here, it is worth noting that the reduced trap density in absorber films could arise from lower GB density caused by the incorporation of SC additives, or could be due to lower bulk trap density as a result of improved growth conditions.

The dependencies of *V*_OC_ and *J*_SC_ on the light intensity for both solar cells were determined to further understand trap-assisted charge recombination loss mechanisms, as shown in Fig. 6e, f. The relationship between *V*_OC_ (and *J*_SC_) and the light intensity *I* can be described by ${V}_{{\mbox{OC}}}\propto (n{k}_{{{{\rm{B}}}}}T/q){{\mathrm{ln}}}(I)$ and ${J}_{{\mbox{SC}}}\propto {I}^{\alpha }$, where *k*_B_ is the Boltzmann constant, *T* is the absolute temperature, *q* is the elementary charge, and *α* and *n* reflect the level of charge-carrier recombination^68–70^. Under open-circuit, all photogenerated charge-carriers recombine. For ideal trap-free solar cells, the slope of *V*_OC_ versus ln(*I*) is *k*_B_*T*/*q*. The slopes for the control and 4mM-SC devices were found from fitting to be 2.31*k*_B_*T*/*q* and 1.87*k*_B_*T*/*q*, respectively. The reduced slope for the 4mM-SC device reflects the suppression of trap-assisted charge-carrier recombination. The parameter *α* should ideally be unity, but *α* < 1 suggests the loss of photogenerated charge-carriers caused by the incomplete collection. The *α* values for the control and 4mM-SC devices are estimated to be 0.924 and 0.951, respectively, implying improved charge-carrier collection for the 4mM-SC device^70^. Impedance spectroscopy analysis (Supplementary Fig. 22 and Supplementary Table 8) shows the 4mM-SC device delivers an increased recombination resistance at the CdS/Sb_2_S_3_ interface (*R*_rec_ = 10.66 kΩ cm^2^) compared to the control device (*R*_rec_ = 4.51 kΩ cm^2^). The increase in the recombination resistance suggests suppressed charge-carrier recombination and improved charge-carrier collection for the 4mM-SC device. The reduced *J*_0_ and diode ideality factor, obtained from the analysis of *J–V* curves of both devices measured in the dark (Supplementary Fig. 23 and Supplementary Note 6), is also consistent with suppression of trap states in the absorber films^69^.

## Discussion

There have been substantial efforts over the past decade to develop light absorbers that can replicate the exceptional optoelectronic properties of lead-halide perovskites and overcome their toxicity and stability limitations^71,72^. A critical limitation of many of these perovskite-inspired materials (especially with Sb- and Bi-based compounds) is carrier localization, which has been so widely found that it was being labeled a hallmark of these materials^73^. This prevents their performance from approaching that of lead-halide perovskite solar cells, despite exhibiting improved stability^71^. Sb_2_S_3_ was thought to be an example of a material with this limitation^30^. Despite continuous improvements in the performance of Sb_2_S_3_ solar cells over the past decade, device optimization of these materials has mostly been based on trial-and-error materials engineering approaches. A more general understanding of the physics behind charge-carrier generation, recombination, and transport is an indispensable prerequisite for revealing fundamental limitations and prospects of Sb_2_S_3_ for optoelectronic applications, as well as offering essential feedback for further device improvements. Our experimental and computational results here demonstrate that the previously-proposed hypothesis of self-trapping in Sb_2_S_3_ is not present, showing that Sb_2_S_3_ avoids the carrier localization limitations prevalent among other Sb- and Bi-based solar absorbers, and that this material has the potential to reach significantly higher efficiencies. This motivates detailed investigations to understand the underlying principles behind the delocalized nature of charge-carriers in Sb_2_S_3_, which can be generalized to other classes of perovskite-inspired materials.

Although Yang et al. observed PL that was red-shifted to the optical gap, and used this as part of their proposition of self-trapping in Sb_2_S_3_, other studies reported broad PL emission near the band-edge, with significant temperature-dependence of the PL intensity, which is consistent with defect-dominated emission^64,74^. Optical pump terahertz probe (OPTP) measurements are also often used to probe whether self-trapping occurs. But previous room-temperature OPTP measurements of solution-processed Sb_2_S_3_ films were inconclusive on whether the initial fast decay in photoconductivity arose from self-trapping or defect-mediated recombination^64^. In this regard, the temperature-dependent mobility measurements we obtained here provide a clearer indication of large polarons being present. Furthermore, extensive investigations into Sb_2_S_3_ films have shown high defect densities >10^15 ^cm^−^^3^ to be common, with a strong correlation between the concentration of defects and device performance^6,66,75^. These experimental findings support our findings made here, where lowering defect densities in the Sb_2_S_3_ films was critical to achieve the high *V*_OC_s obtained. Moreover, our previous first-principles analyses have also challenged the hypothesis of self-trapping in Sb_2_S_3_, where delocalized electrons and holes were found to occur in a perfect supercell^32^. Taken together, our new experimental and computational analyses of defects, coupled with prior polaron calculations, disprove the hypothesis of self-trapping in Sb_2_S_3_, showing that it is possible to improve the performance of this material towards its radiative limit through defect engineering.

In conclusion, we have succeeded in overcoming the 800 mV *V*_OC_ threshold that has been a bottleneck on progress with Sb_2_S_3_ photovoltaics. This achievement was realized through the formation of large-grained thin films by adding SC to the CBD precursor solution. The growth of high-quality uniform films produces Sb_2_S_3_ solar cells with a PCE of 7.67% under 1-sun illumination and a *V*_OC_ of 824 mV, which is the current record value. Temperature-dependent mobility measurements confirm large polaron formation in Sb_2_S_3_, rather than small polarons or STEs. Defect characterization and computations reveal that the reduced defect activity in Sb_2_S_3_ films strongly correlates with the increase in device *V*_OC_. We therefore conclude that antimony chalcogenides have the potential to approach their fundamental limit in performance for optoelectronic devices through careful defect engineering. By comparing defect calculations with the energy of the two deep traps determined from DLTS measurements, we suggest S vacancies and Sb on S anti-sites to be critical defects to address.

## Methods

### Materials

Ethanol (99.0%), acetone (99.0%), methanol (99.0%), ammonia solution (25%-28%, in water), thiourea (99.0%), cadmium chloride (CdCl_2_·2.5H_2_O, 99.0%), antimony potassium tartrate (C_4_H_4_KO_7_Sb·0.5H_2_O, 99.0%), and sodium thiosulfate (Na_2_S_2_O_3_·5H_2_O, 99.0%) were purchased from Sinopharm. Cadmium nitrate (Cd(NO_3_)_2_·4H_2_O, 99.0%) were purchased from Macklin. SnO_2_ colloidal dispersion (15.0% in H_2_O) was purchased from Alfa Aesar. Sodium citrate (C_6_H_5_O_7_Na_3_·2H_2_O, 99.0%), acetonitrile (99.9%), chlorobenzene (99.5%), and tert-butylpyridine (TBP, 96%) were purchased from Aladdin. 2,2’,7,7’-tetrakis(N,N-di-p-methoxyphenylamine)-9,9’-spirobifluorene (Spiro-OMeTAD,99.8%) and lithium bis(trifluoromethylsyfonyl)imide salt (99.95%) were purchased from Xi’an Polymer Light Technology. [6,6] phenyl-C61-butyric acid methyl ester (PC_61_BM, 99.5%) was purchased from Xi’an Yuri Solar Energy Technology. Gold wires were purchased from Tim Advanced Materials. Sb_2_S_3_ powders (99.999%, 100 mesh) were purchased from Beijing Hawk Science & Technology. All the chemicals and materials were purchased and used as received.

### Device fabrication

The SnO_2_/CdS ETL was deposited onto pre-cleaned fluorine-doped tin oxide (FTO, ~15 Ω sq^−^^1^, NSG) conductive glass substrates. The detailed fabrication processes can be referred to from our previous work^14^. Specifically, the commercial SnO_2_ colloidal dispersion diluted seven times with deionized (DI) water was spun onto the FTO substrate at a speed of 3000 rpm for 30 s to form an ultrathin SnO_2_ layer, followed by annealing at 150 °C for 30 min. The CdS buffer layer was deposited using the CBD method. Typically, 30 mL of 15 × 10^−3^ M Cd(NO_3_)_2_·4H_2_O and 39 mL of ammonia solution in water were mixed together and stirred for 2 min, followed by adding 19.2 mL of 1.2 M thiourea and 210 mL of DI water and stirring for another 2 min. The FTO/SnO_2_ substrates were vertically immersed in such precursor solutions and maintained at 65 °C for 14 min with continuous stirring. Here, the thicknesses of SnO_2_ and CdS layers in FTO/SnO_2_/CdS substrates were about 10 and 60 nm, respectively. Afterward, as-prepared CdS layers were treated with CdCl_2_ by spin-coating 0.088 M CdCl_2_·2.5H_2_O in methanol solution onto CdS, followed by annealing in air at 400 °C for 10 min. The Sb_2_S_3_ films were further deposited onto FTO/SnO_2_/CdS substrate via a CBD method. Typically, C_4_H_4_KO_7_Sb·0.5H_2_O and Na_2_S_2_O_3_·5H_2_O were employed as Sb and S sources, respectively. 0.5009 g C_4_H_4_KO_7_Sb·0.5H_2_O (37.5 mM) and 1.985 g Na_2_S_2_O_3_·5H_2_O (200 mM) were dissolved into 40 mL DI water in a small bottle, stirred for 6 min. For the case with the use of SC additive, a small amount of SC with different concentrations of 1 mM, 2 mM, 4 mM, and 8 mM were added into the above mixture solution. The substrates were immersed in the precursor solution with the conducting side facing down and tilted at a certain angle of ~58° to the wall of the bottle. The bottle was then sealed and kept at 95 °C for a certain duration (8 h unless otherwise stated). The substrates were taken out immediately once the reaction ended, followed by rinsing with DI water and drying with nitrogen flow. The as-deposited films were then annealed at 370 °C for 10 min in a nitrogen-filled glovebox. Furthermore, the Spiro-OMeTAD layer was spin-coated onto the Sb_2_S_3_ layer as the HTL, and the detailed deposition procedure for Spiro-OMeTAD can be found in the literature^14^. Specifically, the HTL precursor was prepared by mixing 36.6 mg spiro-OMeTAD, 14.5 μL tert-butylpyridine, and 9.5 μL 0.52 g mL^−1^ of lithium bis(trifluoromethylsyfonyl)imide salt dissolved in acetonitrile in 1 mL chlorobenzene. Then the as-obtained precursor was deposited onto absorber films by spin-coating at a speed of 3000 rpm for 30 s, followed by annealing at 100 °C for 10 min on a hot plate. Finally, the superstrate Sb_2_S_3_ solar cells were completed by evaporating a ~ 80 nm gold layer as the back electrode.

### Materials and devices characterization

The XRD characterization of film samples was performed on a diffractometer (X’Pert PRO MPD, *λ* = 1.54056 Å). SEM images were collected on a JEOL field emission scanning electron microscope (JSM-6700F). The GB densities of films were obtained by depicting the GB grooves in corresponding SEM images using Adobe Photoshop software. AFM images were recorded on a Bruker atomic force microscope (Dimension Icon). Absorption spectra were recorded using a CARY 5000 Agilent spectrophotometer. UPS spectra were collected on a Thermo ESCALAB XI instrument. *J–V* curves of devices were measured under 1-sun illumination (AM 1.5 G, 100 mW cm^−^^2^, calibrated with a standard cell from Newport) using a Newport Oriel Sol 3 A Solar Simulator, combined with a Keithley 2400 digital source meter. The active device area was defined using a 0.06-cm^2^ mask. EQE measurements were performed on a QTest Station 1000 ADI system (Crowntech. Inc) under the DC mode, and the excitation source was a 300 W xenon lamp (CT-XE-300) which was split into specific wavelengths via a M24-S 1/4 m monochromator. The IPV efficiencies of Sb_2_S_3_ solar cells were measured on a home-made platform. The lux levels of WLED lamps (Ccobalance T5/5w) were measured using a luxmeter (TES-1330A), and the spectral irradiance of the diffuse indoor light source of 3000 K WLED was obtained with a high-precision fiber-optic spectrometer (Maya2000 Pro, Ocean Optics). The illustration of the home-made platform and calibration of the spectrometer can be referred to our previous work^35^. Impedance spectra were acquired on a Zahner workstation (Zennium Pro.) under an applied voltage of 0.7 V, a scanning range of 0.5 Hz to 1 MHz, and an AC amplitude of 20 mV under dark; the impedance data were then fitted using a Z-view software. DLTS measurements were performed using a Phystech FT-1230 HERADLTS system. The samples were mounted on a SEMISHARE SCG-O-2 cryostat stage controlled by a Lakeshore 336 temperature controller. DLTS spectra were collected with a reverse bias of –0.3 V and a trap filling forward pulse amplitude of 0.3 V, and the filling pulse length was 10 ms. Ultrafast TA spectra of films were collected using a Helios Ultrafast pump-probe system. For this system, a nondegenerate pump-probe configuration was used to probe the transient dynamics within the femtosecond to nanosecond time range (50 fs to 7 ns) under ambient conditions. The pump pulses at a wavelength of 400 nm were generated by doubling the 800 nm pulse using a beta barium borate crystal on an optical parametric amplifier. The white light continuum probe pulses were formed by 800 nm femtosecond with a 2 mm sapphire plate for the 400–800 nm range. TOF measurements were performed under 100 to 400 K based on a device configuration of FTO/Sb_2_S_3_/Au, which was contained in an optically and electrically shielded box. Here, the thick Sb_2_S_3_ film was deposited using CSS, and the processing conditions are detailed in our previous work^76^. The thickness of Sb_2_S_3_ film was measured to be around 2.2 μm using a KLA-Tencor D100 step profilometer based on three measurements for each sample. TOF measurements were carried out using a Keithley 2400 as the power source, and an Agilent DSOS054A high-definition oscilloscope for acquiring the transient signals. A pulsed laser with 405 nm wavelength, corresponding to ~100 nm absorption depth in Sb_2_S_3_, was used to excite the thick Sb_2_S_3_ film from the transparent FTO side. The hole mobility could be extracted by ${\mu }_{{{{\rm{h}}}}}={d}^{2}/V{\tau }_{{{{\rm{t}}}}}$, where *μ*_h_ is hole mobility, *d* is film thickness, *V* is the driving voltage varying from 36 to 72 V and *τ*_t_ is the transit time defined by the turning point in the bilogarithmic plot of photocurrent versus decay time.

### Computational methods

The formation energy of a defect *D* in charge state *q* is obtained by:

1 $$\triangle {H}_{f}^{D,q}={E}^{D,q}-{E}^{{{{\rm{pristine}}}}}-{\sum}_{i}{n}_{i}{\mu }_{i}+q{E}_{{{{\rm{F}}}}}+{E}_{{{{\rm{corr}}}}}$$

Here, ${E}^{D,q}$ and ${{E}}^{{{{\rm{pristine}}}}}$ denote the total energies of the defective and pristine supercells, respectively. The term ${\sum}_{i}{n}_{i}{\mu }_{i}$ accounts for the energy change associated with adding (*n*_*i*_ > 0) or removing (*n*_*i*_ < 0) atoms of type *i*, with *μ*_*i*_ being the corresponding chemical potential. *E*_corr_ corrects for finite-size effects arising from charged defects in periodic boundary conditions, following the approach developed by Kumagai and Oba^77^. The underlying density functional theory (DFT) calculations are performed using the HSE06 functional with a plane wave basis set as detailed in ref. ^50^.

The thermodynamic charge transition level *ε*(*q*_1_/*q*_2_) is calculated as:

2 $$\varepsilon \left({q}_{1}/{q}_{2}\right)=\frac{\triangle {H}_{f}^{D,{q}_{1}}\left({E}_{{{{\rm{F}}}}}=0\right)-\triangle {H}_{f}^{D,{q}_{2}}\left({E}_{{{{\rm{F}}}}}=0\right)}{{{q}_{2}-q}_{1}}$$

where $\triangle {H}_{f}^{D,q}\left({E}_{F}=0\right)$ represents the formation energy of defect *D* in charge state *q* when the Fermi level is at the VBM.

The equilibrium concentration of defect *D,q* is determined by:

3 $${C}_{D,q}=g{N}_{D}{e}^{\frac{-\triangle {H}_{f}^{D,q}}{{k}_{{{{\rm{B}}}}}{T}_{{{{\rm{anneal}}}}}}}$$

where *g* accounts for degeneracies from geometry and spin. *N*_*D*_ is the number of defect sites per unit volume in the supercell. *k*_B_ is the Boltzmann constant, and *T*_anneal_ is the annealing temperature.

The capture cross-section is calculated by

4 $$\sigma=\frac{C}{\left\langle v\right\rangle }$$

where $\left\langle v\right\rangle=\sqrt{3{k}_{{{{\rm{B}}}}}T/{m}^{*}}$ is the average thermal velocity of the carrier, with ${m}^{*}$ the average effective mass. The carrier capture coefficient *C* is derived from the Fermi’s golden rule^78,79^.

5 $$C=\frac{2\pi }{\hslash }{Vg}{\sum}_{m}{\omega }_{m}\left\langle {\psi }_{i}\left| \frac {\partial \hat{h}}{\partial Q}\right|{\psi }_{f}\right\rangle {\sum}_{n}{\left|\left\langle {\chi }_{{im}}\left|Q-{Q}{_0}\right|{\chi }_{{fn}}\right\rangle \right|}^{2}\delta {\left(\Delta E+m\hslash {\Omega }_{i}-n{{\hslash }}{\Omega }_{f}\right)}$$

Here, *V* is the volume of the supercell, and *g* represents the degeneracy factor, accounting for the number of equivalent transition pathways. The subscripts *i* and *f* indicate the initial and final states, respectively. The quantum numbers of the ionic states are denoted as *n* and *m*, while ${\omega }_{m}$ represents the thermal occupation. $\hat{h}$ and ${\psi }_{\{i,f\}}$ correspond to the single-particle Hamiltonian and single-particle wavefunctions, respectively. ${\Omega }_{\{i,f\}}$ refer to the phonon frequencies associated with the initial and final states.

A one-dimensional generalized coordinate *Q* is used to represent the atomic relaxation

6 $${Q}^{2}={\sum}_{\alpha }{M}_{\alpha }\Delta {R}_{\alpha }^{2}$$

where ${M}_{\alpha }$ and $\Delta {R}_{\alpha }$ are the mass and displacement of atom *α* between the initial and final states, respectively.

The maximum power conversion efficiency (${\eta }_{\max }$) of a solar cell is expressed as:

7 $${\eta }_{\max }={\max }_{V}\left(\frac{{JV}}{e{\int }_{0}^{\infty }E{\phi }_{{{{\rm{sun}}}}}\left(E\right){{{\rm{d}}}}E}\right)$$

where *e* is the elementary charge. ${\phi }_{{{{\rm{sun}}}}}\left(E\right)$ is the incident spectral photon flux density at the photon energy *E*. A standard AM1.5 solar spectrum is used in this work.

Following the methodology by Kim et al.^80,81^, the net current density $J\left({V;W}\right)$, accounting for both radiative and non-radiative recombination, is given by:

8 $$J\left(V;W\right)={J}_{{{{\rm{SC}}}}}\left(W\right)+{J}_{0}^{{{{\rm{rad}}}}}\left(W\right)\left(1-{e}^{\frac{{eV}}{{k}_{{{{\rm{B}}}}}T}}\right)-{eR}\left(V\right)W$$

where $R\left(V\right)$ is the defect-mediated non-radiative recombination rate. *W* is the film thickness. ${J}_{0}^{{{{\rm{rad}}}}}$ is the saturation current density.

Assuming that each absorbed photon contributes one collected electron hole pair, the short-circuit current density ${J}_{{{{\rm{SC}}}}}$ is given by

9 $${J}_{{SC}}(W)=e{\int }_{{E}_{g}}^{\infty }a({E;W}){\phi }_{{sun}}(E){dE}$$

The radiative limit is obtained by setting the defect mediated non-radiative recombination term *R*(*V*) to zero and solving $J\left({V}_{{{{\rm{OC}}}}}^{{{{\rm{rad}}}}}{;W}\right)$ = 0, giving

10 $${V}_{{{{\rm{OC}}}}}^{{{{\rm{rad}}}}}\left(W\right)=\frac{{k}_{{{{\rm{B}}}}}T}{e}{{\mathrm{ln}}}\left(\frac{{J}_{{{{\rm{SC}}}}}\left(W\right)}{\,{J}_{0}^{{{{\rm{rad}}}}}\left(W\right)}+1\right)$$

The radiative saturation current density is calculated from detailed balance as

11 $$\,{J}_{0}^{{rad}}(W)=e{\int }_{0}^{\infty }a({E;W}){\phi }_{{{\rm{bb}}}}(E){{{\rm{d}}}}{E}$$

where ${\phi }_{{{{\rm{bb}}}}}\left(E\right)$ is the black body photon flux. The photon absorptivity $a({E;W})$ describes the probability of absorption as a function of photon energy. In the Shockley–Queisser model, it is idealized as a step-like function at the bandgap. In our calculation, the absorptivity is defined as $a\left({E;W}\right)=1-{e}^{-2\alpha \left(E\right)W}$, where α(E) is the optical absorption coefficient of Sb_2_S_3_ obtained from first-principles calculations using the HSE06 functional^50^. The factor of 2 accounts for a double-pass optical path through the absorber, assuming perfect reflection from the back contact. A film thickness of *W* = 350 nm was used to match the experimental absorber thickness.

### Reporting summary

Further information on research design is available in the Nature Portfolio Reporting Summary linked to this article.

## Supplementary information

Supplementary Information

Solar Cells Reporting Summary

Transparent Peer Review file

## Data Availability

The experimental and computational data generated in this paper and in the Supplementary Information have been deposited in the Oxford Research Archive (ORA) Data Repository, with the link https://doi.org/10.5287/ora-8j95djknz.
