# Supplementary material for: Antimony sulfide photovoltaics with high open-circuit voltage not limited by self-trapped excitons
Source: Nat Commun. 2026 Aug 19;17:9914. doi: 10.1038/s41467-026-76845-1 (PMC13583045; doi:10.1038/s41467-026-76845-1)
Supplement: Supplementary file 1 [file 41467_2026_76845_MOESM1_ESM.pdf]

Supplementary Information for

Antimony sulfide photovoltaics with high open-circuit voltage not limited by self-trapped excitons

Jiacheng Zhou[1,†], Xinwei Wang[2,†], Tianle Shi[1,†], Lei Wan[1], Junzhi Ye[3], Zhiqiang Li[4], Aron Walsh[2,*], Robert L. Z. Hoye[3,*], Ru Zhou[1,*]

[1] School of Electrical Engineering and Automation, Hefei University of Technology, Hefei 230009, P. R. China

[2] Department of Materials, Imperial College London, Exhibition Road, London SW7 2AZ, United Kingdom

[3] Inorganic Chemistry Laboratory, Department of Chemistry, University of Oxford, South Parks Road, Oxford OX1 3QR, United Kingdom

[4] National-Local Joint Engineering Laboratory of New Energy Photoelectric Devices, College of Physics Science and Technology, Hebei University, Baoding 071002, P. R. China

[†]These authors contributed equally: Jiacheng Zhou, Xinwei Wang, Tianle Shi

* Corresponding authors: Aron Walsh (a.walsh@imperial.ac.uk), Robert L. Z. Hoye (robert.hoye@chem.ox.ac.uk), Ru Zhou (zhouru@hfut.edu.cn)

Supplementary Note 1. Deposition mechanism of Sb_2S_3 films

We investigated the underlying mechanism that could explain the impact of the SC complexing agent on the properties of Sb_2S_3 films. According to the XRD characterization discussed earlier, it is reasonable to assume that SC only plays roles during the CBD of Sb_2S_3 thin films. For the CBD of films, the substrate is exposed to soluble precursors which react or decompose to form the desired film materials[1]. The nucleation of Sb_2S_3 occurs when the ionic product ($IP=[Sb^{3+}]^2[S^{2-}]^3$) exceeds the solubility product of Sb_2S_3 ($K_{sp}=10^{-92.77}$)[2]. From the perspective of nucleation and growth, an intricate balance of growth parameters is necessary to achieve high-quality films. The formation of Sb_2S_3 grains typically involves the formation of stable nuclei and the growth of nuclei into network[3, 4, 5]. The supersaturation of precursors leads to the formation of monomers (atoms, ion, molecules, or formula units), which would cluster together into nuclei. Such nuclei are likely to appear at the substrate/precursor-solution interface and/or within the precursor solution, referred to as heterogeneous nucleation and homogeneous nucleation, respectively. In the case of classical homogeneous nucleation, the nucleation rate exponentially decreases with the homogenous Gibbs free energy (ΔG_{homo}^*), which can be described by Eq. S1[4]:

$$\Delta G_{\mathrm{homo}}{}^{*}=\frac{4}{3}\pi r_k^3 n\Delta G_v+4\pi r_k^2 n\gamma_{\mathrm{SP}} \tag{S1}$$

where r_k presents the critical radius of the nuclei, n is the number of nuclei per unit volume, ΔG_v corresponds to the change in free energy between the nuclei and solution per unit volume, γ_{SP} is the particle surface energy, θ is the contact angle. The free energy of heterogeneous nucleation, i.e., nucleation barrier (ΔG_{hetero}^*) can be described as Eq. S2, by introducing a factor of $f(\theta)$ which depends on the contact angle (θ):

$$\Delta G_{\mathrm{hetero}}{}^{*}=f(\theta)\Delta G_{\mathrm{homo}}{}^{*} \tag{S2}$$

where the factor $f(\theta)$ can be defined as Eq. S3 and S4:

$$f(\theta)=\frac{(2+\cos\theta)(1-\cos\theta)^2}{4} \tag{S3}$$

$$\cos\theta=\frac{\gamma_{\mathrm{SL}}-\gamma_{\mathrm{CS}}}{\gamma_{\mathrm{CL}}} \tag{S4}$$

In Eq. S4, the terms γ_{CL}, γ_{SL} and γ_{CS} denote the interfacial energies between the crystalline phase and the liquid, the substrate and the liquid, and the crystalline phase and the solid surface, respectively.

The free energy of heterogeneous nucleation, i.e., nucleation barrier (ΔG_{hetero}^*), can be expressed by multiplying ΔG_{homo}^* with a factor of $f(\theta)$ which depends on the contact angle (θ). Here we measured the water contact angles on the substrates used for Sb_2S_3 deposition to understand the role of SC on the nucleation behavior of Sb_2S_3. Supplementary Fig. 15a schematically shows the contact angle (θ) in an interfacial energy diagram for three phases of two solids and a liquid in contact, and the water contact angles of the pristine CdS substrate, as well as the CdS substrate treated by precursor solutions with different SC concentrations for 45 min CBD. As shown, the water contact angle of the pristine CdS substrate is 76.0°, and the contact angle is gradually reduced to 65.3°, 58.2°, 53.8°, and 45.4° for the substrates involving the treatments by precursor solutions with 1 mM, 2 mM, 4 mM, and 8 mM SC. According to the ratio of the free energies of homogeneous and heterogeneous nucleation as a function of the contact angle, as shown in Supplementary Fig. 15b, a reduced contact angle would result in a decrease in the nucleation barrier for the heterogeneous nucleation and thus promote the nucleation rate of Sb_2S_3 on the substrate. Therefore, we propose that the incorporation of SC would enhance the heterogeneous nucleation of Sb_2S_3 on the substrate.

Next, we propose a plausible mechanism for the deposition of Sb_2S_3 films on the substrate, as schematically illustrated in Supplementary Fig. 15c. In the conventional precursor solution without the addition of SC, the APT slowly releases Sb^{3+} ions while thiosulfate ions are hydrolyzed to release S^{2-} ions in the acidic medium[6]. The deposition of Sb_2S_3 with the addition of SC as complexing agent can be described by Eq. S5-S12[7, 8]:

(1) The release of Sb^{3+}:

$$[Sb_2(C_4H_2O_6)_2]K_2 \rightarrow [Sb_2(C_4H_2O_6)_2]^{2-} + 2K^+ \quad (S5)$$

$$[Sb_2(C_4H_2O_6)_2]^{2-} + 4H^+ \rightarrow 2Sb^{3+} + 2(C_4H_4O_6)^{2-} \quad (S6)$$

(2) The release of S^{2-}:

$$Na_2S_2O_3 \rightarrow 2Na^+ + S_2O_3^{2-} \tag{S7}$$

$$2S_2O_3^{2-} \rightarrow S_4O_6^{2-} + 2e^- \tag{S8}$$

$$S_2O_3^{2-} + H^+ \rightleftharpoons S + HSO_3^- \tag{S9}$$

$$S + 2e^- \rightarrow S^{2-} \tag{S10}$$

(3) The formation of complexing agent:

$$C_6H_5O_7^{3-} + Sb^{3+} \rightleftharpoons (C_6H_5O_7)Sb \tag{S11}$$

(4) The formation of Sb_2S_3:

$$2Sb^{3+} + 3S^{2-} \rightarrow Sb_2S_3 \tag{S12}$$

Here it is noted that the strong complexation effect of APT would cause the formation of many aggregates in the precursor solution, which serve as homogeneous nucleation centers for Sb_2S_3[9]. The small clusters created within the solution further migrate and adhere onto the substrate surface, resulting in the deposition of Sb_2S_3 films in the cluster mechanism. In contrast, with the addition of SC, the strong complexing effect of citrate causes the formation of Sb^{3+}-citrate complex via electron transfer and thus reduces the amount of APT aggregates in the precursor solution, preventing the bulk precipitation of final products. This leads to a slow release of Sb^{3+} and a more uniform distribution of Sb^{3+} in the solution, allowing the deposition of Sb_2S_3 films on the substrate in a more controlled manner. Therefore, the citrate-mediated CBD is proposed to realize the involution of the deposition mode for Sb_2S_3 films from the cluster deposition to the ion-by-ion deposition. For the ion-by-ion deposition mechanism, the complexing agent enables the slow release of free metal ions to directly react with anions onto the substrate, being capable of forming well textured crystalline or even monocrystalline films[1]. The ion-by-ion deposition therefore promotes the heterogeneous nucleation on the substrate and suppresses the homogeneous nucleation in the precursor solution, similar to the scenario demonstrated for the CBD of other metal chalcogenide thin films, such as ZnS[10]. High-density small nuclei formed on the substrate would further coalesce into a large-grained Sb_2S_3 film during the CBD process. Hence the formation of stable citrate-complexed Sb^{3+} succeeds to control the nucleation

and growth rate of Sb_2S_3, contributing to the deposition of large-grained Sb_2S_3 films. In fact, it has been demonstrated that films grown by ion-by-ion deposition mechanism are usually associated with larger crystalline size compared to those grown by cluster deposition mechanism[1].

Supplementary Note 2. Influence of pH on the deposition of Sb_2S_3 films

Apart from the complexation of citrate, we speculate that pH might be another critical factor influencing the deposition of Sb_2S_3 films. To investigate the influence of pH on the deposition of Sb_2S_3 films, we measured pH of the precursor solutions without and with the addition of SC (Supplementary Fig. 16a-c). It is revealed that the addition of 4 mM SC leads to the increase of pH from 4.61 to 5.73. Here, we further used ammonia solution to adjust the pH of precursor solution to a similar level of 4 mM SC containing precursor. It is found that the thickness of the Sb_2S_3 film prepared with the addition of ammonia solution (383 ± 24 nm) is thinner than that of the control sample (506 ± 26 nm) but thicker than that of the 4mM-SC sample (Supplementary Fig. 16d). This indicates that, besides the complexation of citrate, the increase in pH should also contribute to the reduction in the deposition rate. However, the device performance reveals that, the incorporation of ammonia solution does not lead to remarkable efficiency increase as high as that of the case with the addition of SC (Supplementary Fig. 16e), although both precursor solutions share similar pH values. This implies that the increase in PCE for SC-Sb_2S_3 devices should be mainly associated with the complexation effect of citrate which causes the evolution in the morphology of Sb_2S_3 absorber films.

Supplementary Note 3. Basic principles of DLTS

The transient capacitance change over time can be described using the Eq. S13[11]:

$$C(t)=C_R+\Delta C*\exp\left(-\frac{t}{\tau_e}\right) \quad \text{(S13)}$$

where ΔC is the amplitude of the transient capacitance and τ_e is the electron emission time constant, which is the reciprocal of electron emission rate, expressed by the Eq. S14[12]:

$$\tau_e = \frac{t_2 - t_1}{\ln\left(\frac{t_2}{t_1}\right)} \tag{S14}$$

where t_1 and t_2 determine a fixed time window used to calculate $\Delta C = C_{t2} - C_{t1}$ (Figure 4a). In this work, the time window was set to be 100 ms. As ΔC and τ_e vary with temperature, a sequence of the transient capacitance is further collected at various temperatures. The capacitance changes within a time window (ΔC) *vs.* the different temperatures (T) are sampled as the DLTS signals. For the analysis of DLTS measurements, the activation energy (E_a) and capture cross-section (σ) of defects are determined from the Arrhenius plots using Eqs. S15 and S16:

$$\ln(\tau_e v_{th,n} N_C) = \frac{E_C - E_T}{k_B \mathrm{T}} - \ln(X_n \sigma_n) \tag{S15}$$

$$\ln(\tau_p v_{th,p} N_V) = \frac{E_T - E_V}{k_B \mathrm{T}} - \ln(X_p \sigma_p) \tag{S16}$$

where τ_e and τ_p represents electron and hole emission time constant, respectively ($\tau_p = \tau_e$), E_C, E_V, and E_T are the CB minimum (CBM), valance band maximum (VBM), and defect energy level, respectively. $v_{th,n}$ and $v_{th,p}$ indicates the thermal velocities for electron and hole traps, respectively. X_n and X_p are the entropy factor for hole and electron, respectively. N_C and N_V represent the density of states of CB and VB, respectively. σ_n and σ_p represent the capture cross-section of electron and hole traps, respectively. T and k_B are the temperature and Boltzmann constant, respectively. E_T is the defect level. Here $v_{th,n}$ and N_C can be obtained from Eqs. S17 and S18:

$$v_{th,n} = \sqrt{\frac{3k_B T}{m_n^*}} \tag{S17}$$

$$N_C = 2\left(\frac{2\pi m_n^* k_B T}{h^2}\right)^{\frac{3}{2}} \tag{S18}$$

where m_n^* is the effective mass for electrons, with similar equations for $v_{th,p}$ and N_V. E_a is calculated using E_C–E_T for electron trap and E_T–E_V for hole trap. E_a can be obtained from the slope of Arrhenius plots through linear regression. σ can be extracted from the intersection of a line with the y-axis. The trap density (N_T) can be obtained using Eq. S19[11]:

$$N_T = \frac{2N_S \Delta C}{C_R} \tag{S19}$$

where N_S represents the shallow donor/acceptor concentration in absorber films, C_R and ΔC represent the capacitance at reverse bias in equilibrium and the amplitude of capacitance transient, respectively.

Supplementary Note 4. Franck-Condon relaxation energies, Stokes shifts, and defect related emission energies

The configuration coordinate parameters (shown in Figure 5d, e) can be used to estimate the Franck-Condon relaxation energies and defect transition Stokes shifts.

For a transition between an initial electronic state (i) and a final electronic state (f), the relaxation energies are defined as

$$\lambda_i = E_i(Q_f) - E_i(Q_i) \tag{S20}$$

$$\lambda_f = E_f(Q_i) - E_f(Q_f) \tag{S21}$$

where Q_i and Q_f are the equilibrium configurations of the initial and final states, respectively. The Stokes shift of the defect charge transition is the sum of the two relaxation energies expressed as

$$\Delta E_{\text{Stokes}} = E_{\text{abs}} - E_{\text{em}} = \lambda_i + \lambda_f \tag{S22}$$

The relevant hole-capture transitions shown in Figure 5d, e are $V_{S(3)}^0 + h^+_{VBM} \rightarrow V_{S(3)}^+$ and $Sb_{S(2)}^- + h^+_{VBM} \rightarrow Sb_{S(2)}^0$. Therefore, for $V_{S(3)}$, the initial state i and final state f correspond to $V_{S(3)}^0$ and $V_{S(3)}^+$, respectively. For $Sb_{S(2)}$, the initial state i and final state f correspond to $Sb_{S(2)}^-$ and $Sb_{S(2)}^0$, respectively. The corresponding state labels and definitions of relaxation energies are illustrated schematically in Supplementary Fig. 18. The resulting values are summarized in Supplementary Table 6.

These defect transition Stokes shifts are not the quantity directly probed by PL. The experimental red shifts (about 0.1 eV in this work and about 0.6 eV in Yang et al.) are emission energies relative to the optical gap, $E_g - E_{PL}$, where E_g is the optical gap.

For a defect level at E_t above the VBM, the radiative hole-capture emission energy

is calculated as

$$E_{PL}^{h} = E_t - \lambda_f \tag{S23}$$

The red shift relative to the optical gap is then $E_g - E_{PL}^h$. Using the optical gap E_g = 1.75 eV (Supplementary Fig. 14) and the calculated levels E_t = 0.59 eV ($V_{S(3)}$) and 0.61 eV ($Sb_{S(2)}$), the hole-capture emission energies are listed in Supplementary Table 7.

For $V_{S(3)}$, the hole capture gives E_{PL}^h ~0 eV because λ_f is nearly equal to E_t. This indicates that the relaxed configuration is close to the crossing region of the two potential energy surfaces, consistent with very efficient non-radiative multiphonon capture. For $Sb_{S(2)}$, the calculated hole capture emission energy is also deep in the infrared, around 0.25 eV. These values are below the PL energies associated with either the small red shift measured in this work or the 0.6 eV red shift reported by Yang et al. The latter would correspond to an emission energy of approximately 1.15 eV if referenced to an optical gap of 1.75 eV.

Together with the large calculated hole-capture cross sections, these results indicate that $V_{S(3)}$ and $Sb_{S(2)}$ are efficient deep non-radiative recombination centers. Any radiative signatures from these specific hole-capture transitions are expected to be weak or masked by the competing non-radiative multiphonon capture pathway. We therefore do not assign the measured PL red shift to direct radiative emission from $V_{S(3)}$ or $Sb_{S(2)}$ based on these configuration coordinate diagrams.

Supplementary Note 5. Trap-limited V_{OC} and PCE estimation

To estimate the maximum achievable V_{OC} and PCE of Sb_2S_3 solar cells, we accounted for both radiative (band-to-band) and defect-mediated non-radiative recombination following the methodology of Kim *et al*[13, 14]. Within the conventional S-Q detailed balance framework, we replace the ideal step-function absorptance with the finite-thickness absorptance of Sb_2S_3. A representative film thickness of 350 nm was used to ensure a consistent radiative limit for both samples. Using this finite-thickness absorptance, we calculated the radiative V_{OC} limit to be 1.70 V with a corresponding

maximum PCE of 21.98%. Non-radiative recombination losses were then incorporated using the experimentally measured trap levels, trap densities and capture cross sections from DLTS. Since only hole traps are detected by DLTS, we assume non-radiative recombination in these samples is dominated by hole capture processes (i.e., electron capture coefficients are set infinitely high).

For the control sample, the predicted V_{OC} is 1.15 V, corresponding to a PCE of 13.34% and a non-radiative voltage loss ΔV_{OC} of 0.35 V (defined as $V_{OC}^{SQ} - V_{OC}$, where V_{OC}^{SQ} is calculated to be around 1.50 V). The 4mM-SC sample shows an improved V_{OC} of 1.26 V and a PCE of 14.72%, with a reduced ΔV_{OC} of 0.24 V. The calculated *J*–*V* curves are shown in Supplementary Fig. 19. These predictions are consistent with the experimentally observed improvement in the 4mM-SC device performance.

Supplementary Note 6. Analysis of dark *J-V* curves

According to the abrupt junction *J-V* equation as expressed in Eq. S24, and its formula manipulation as given in Eq. S25-S27, the parameters of junction ideality factor (*A*), the reverse saturation current density (J_0), the series resistance (R_s), and the shunt conductance (*G,* i.e., $1/R_{sh}$) can be extracted[15]:

$$J=J_0\exp(\frac{V-JR_s}{AV_{th}})+GV-J_L \quad \text{(S24)}$$

$$\frac{dJ}{dV}=\frac{1}{AV_{th}}\times J_0\exp(\frac{V-JR_s}{AV_{th}})+G \quad \text{(S25)}$$

$$\frac{dV}{dJ}=R_s+\frac{AV_{th}(1-G\frac{dV}{dJ})}{J+J_L-GV} \quad \text{(S26)}$$

$$\ln(J+J_L-GV)=\frac{1}{AV_{th}}\times(V-R_sJ)+\ln J_0 \quad \text{(S27)}$$

where J_L is the photocurrent density, *q* is the electron charge, V_{th} is the thermal energy (k_BT/q) and is generally regarded to be 0.026 eV at room temperature, k_B is the Boltzmann's constant.

As shown in Supplementary Fig. 23, compared to the control device, the 4mM-SC device has lower R_S and *G*, which implies an improvement in the carrier extraction capability. The *A* values extracted from the control and 4mM-SC devices are 2.38 and 1.91, respectively; the reduced *A* value indicates the suppression of defect states in

absorber films. The calculated J_0 is reduced from 3.22×10^{-5} mA cm^{-2} for the control device to 2.09×10^{-6} mA cm^{-2} for the 4mM-SC device. The main reason for the generation of J_0 is the charge-carrier recombination caused by deep-level defects in the devices, and thus the reduced J_0 implies the suppression of the defect-induced recombination, contributing to the V_{OC} increase for the 4mM-SC device.

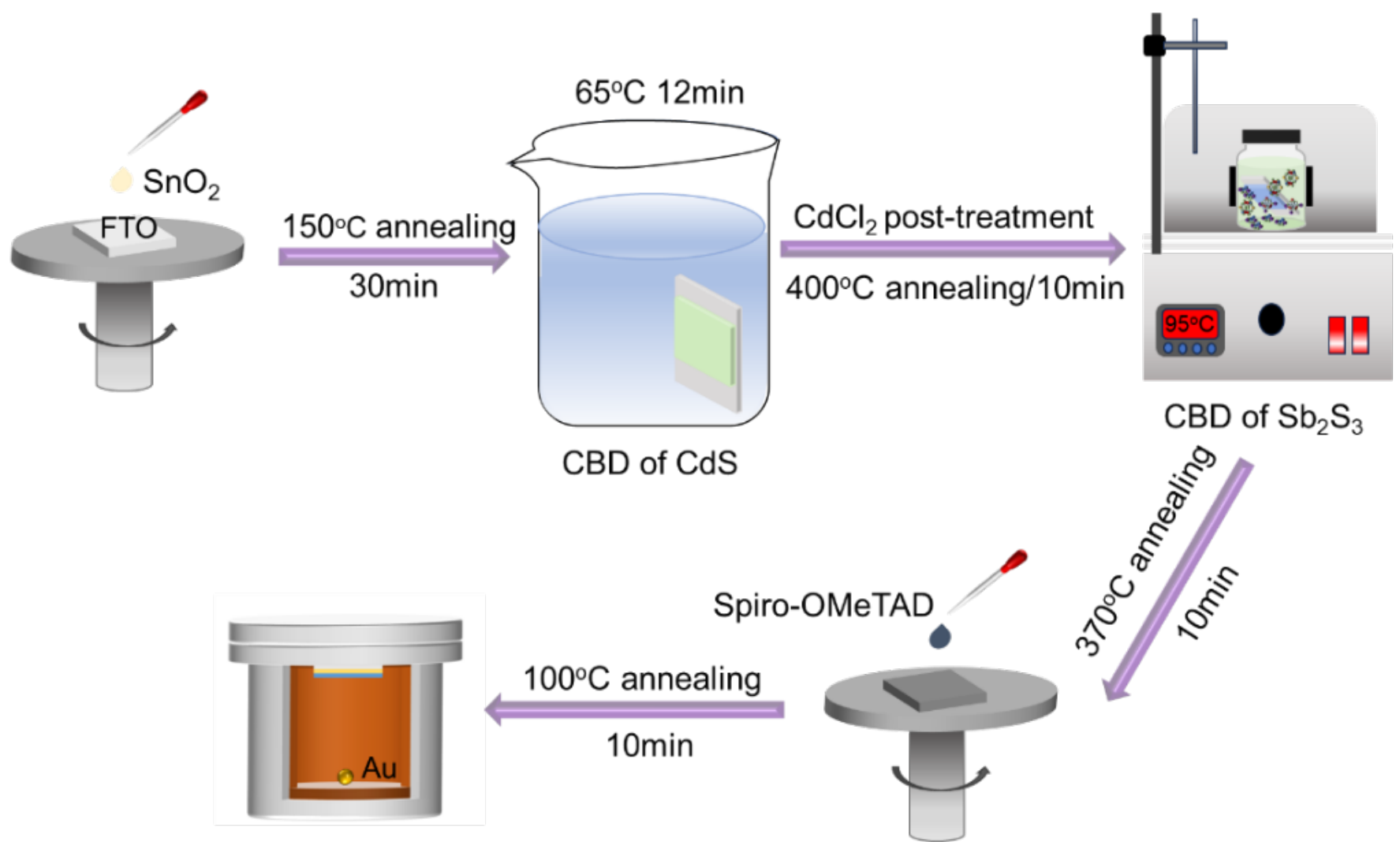

Supplementary Fig. 1 | The device fabrication processes. The detailed process flow diagram for fabricating Sb_2S_3 solar cells.

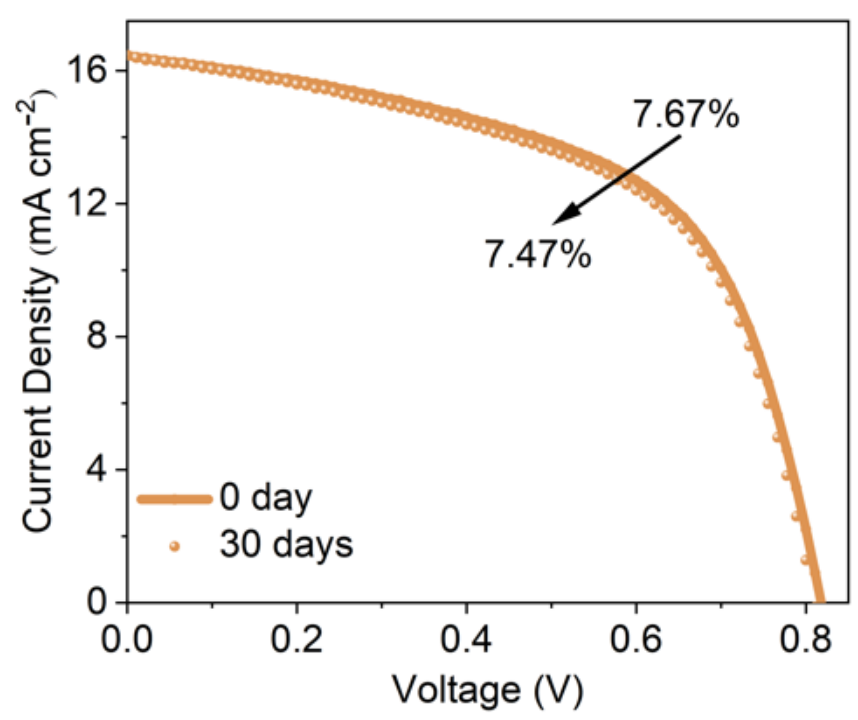

Supplementary Fig. 2 | Device stability of Sb_2S_3 solar cells. Device stability of unencapsulated 4mM-SC Sb_2S_3 solar cells stored for 30 days in an electronic cabinet with 15% humidity under the ambient temperature.

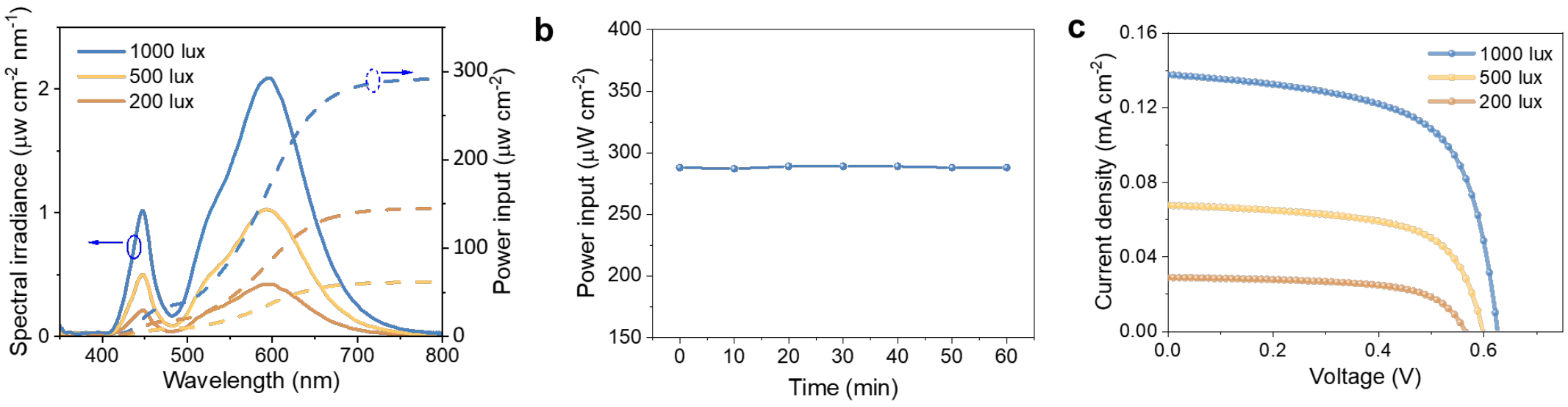

Supplementary Fig. 3 | The IPV performance of Sb_2S_3 solar cells. a Spectral irradiance of the WLED light source at 1000, 500, 200 lux illuminances. The associated irradiances are calculated to be 291.9, 145.2, 62.0 μW cm^{-2}, respectively, which were calculated by integrating the absolute spectral irradiances across all wavelengths. **b** Stability of the WLED light source on the device plane. The light source used for IPV measurements show excellent stability. **c** *J–V* curves of the 4mM-SC Sb_2S_3 solar cells measured under 1000, 500, and 200 lux illuminances.

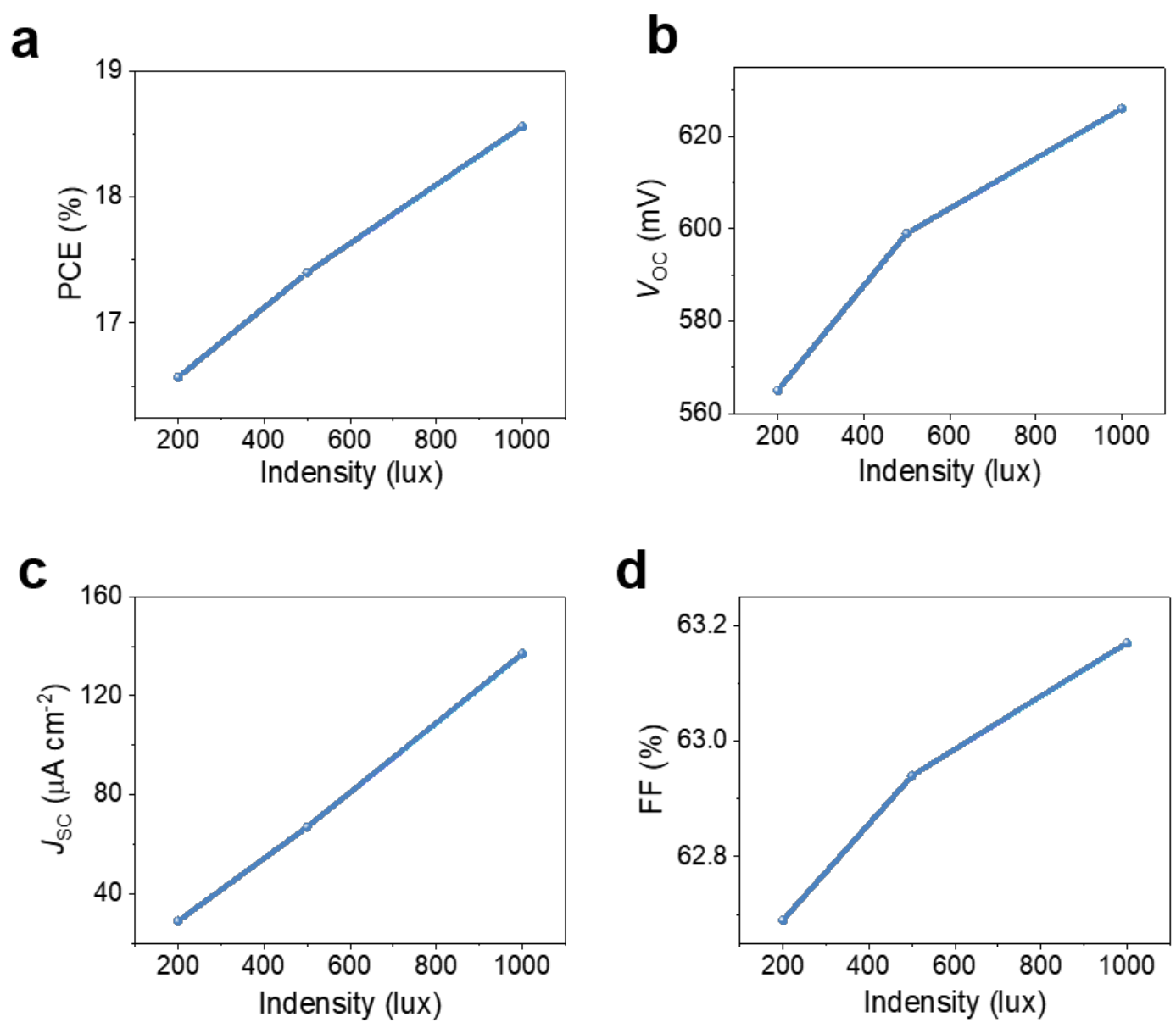

Supplementary Fig. 4 | The dependences of Sb_2S_3 device parameters on the indoor light intensity. a PCE, **b** V_{OC}, **c** J_{SC}, **d** FF.

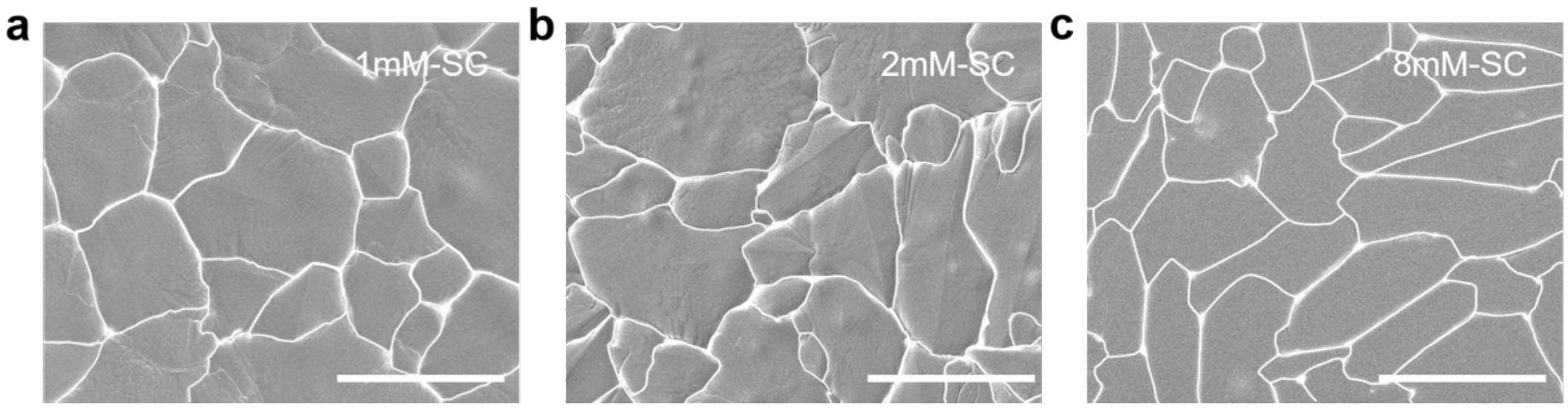

Supplementary Fig. 5 | Top-view SEM images of Sb_2S_3 films prepared with the use of other SC concentrations. **a** 1 mM, **b** 2 mM, and **c** 8 mM SC. The GBs are highlighted to clearly show the changes in the GB. Scale bar, 5 µm.

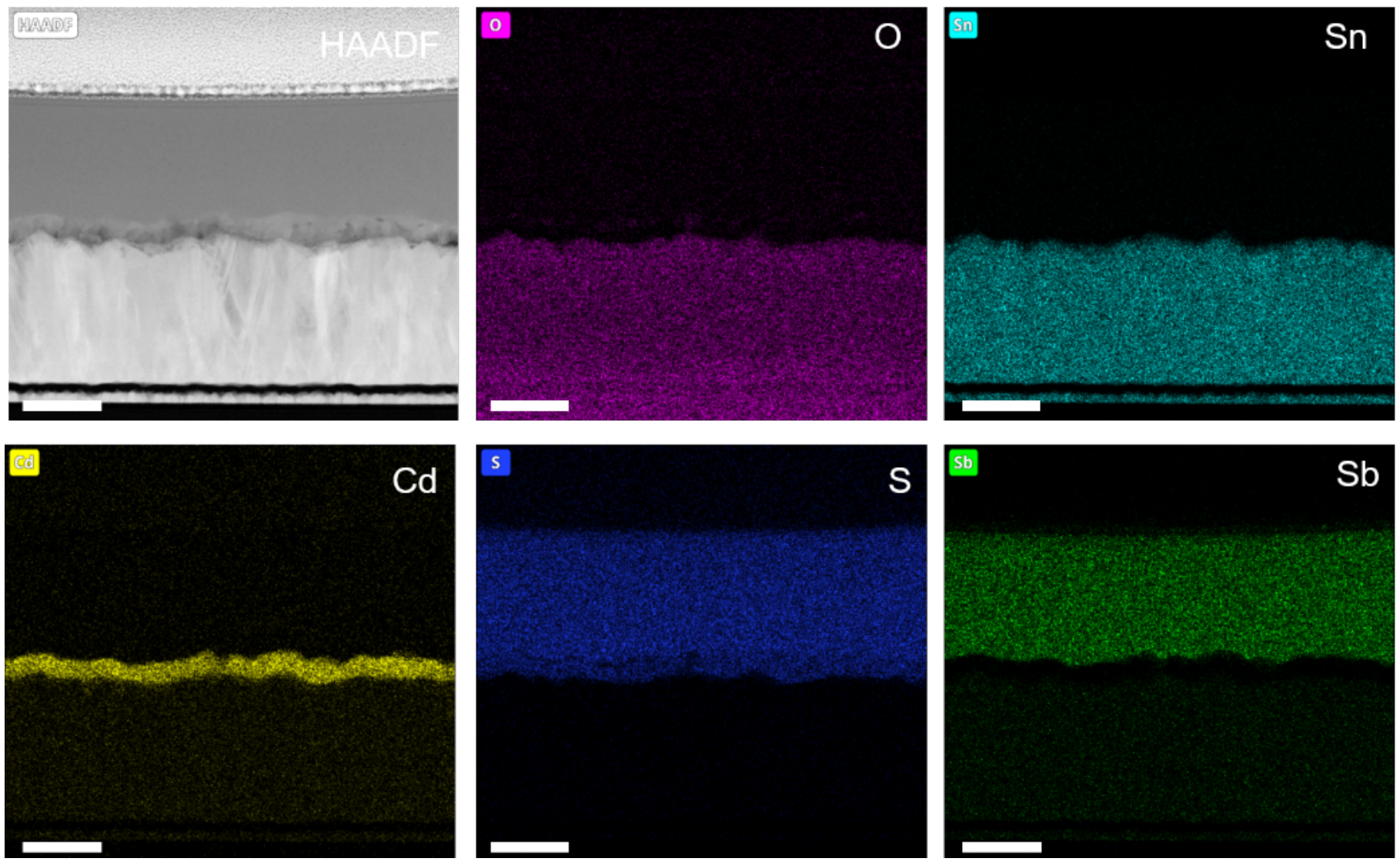

Supplementary Fig. 6 | Cross-sectional elemental mapping images of the 4mM-SC Sb_2S_3 film. The sample deposited on FTO/SnO_2/CdS substrate. Scale bar, 200 nm.

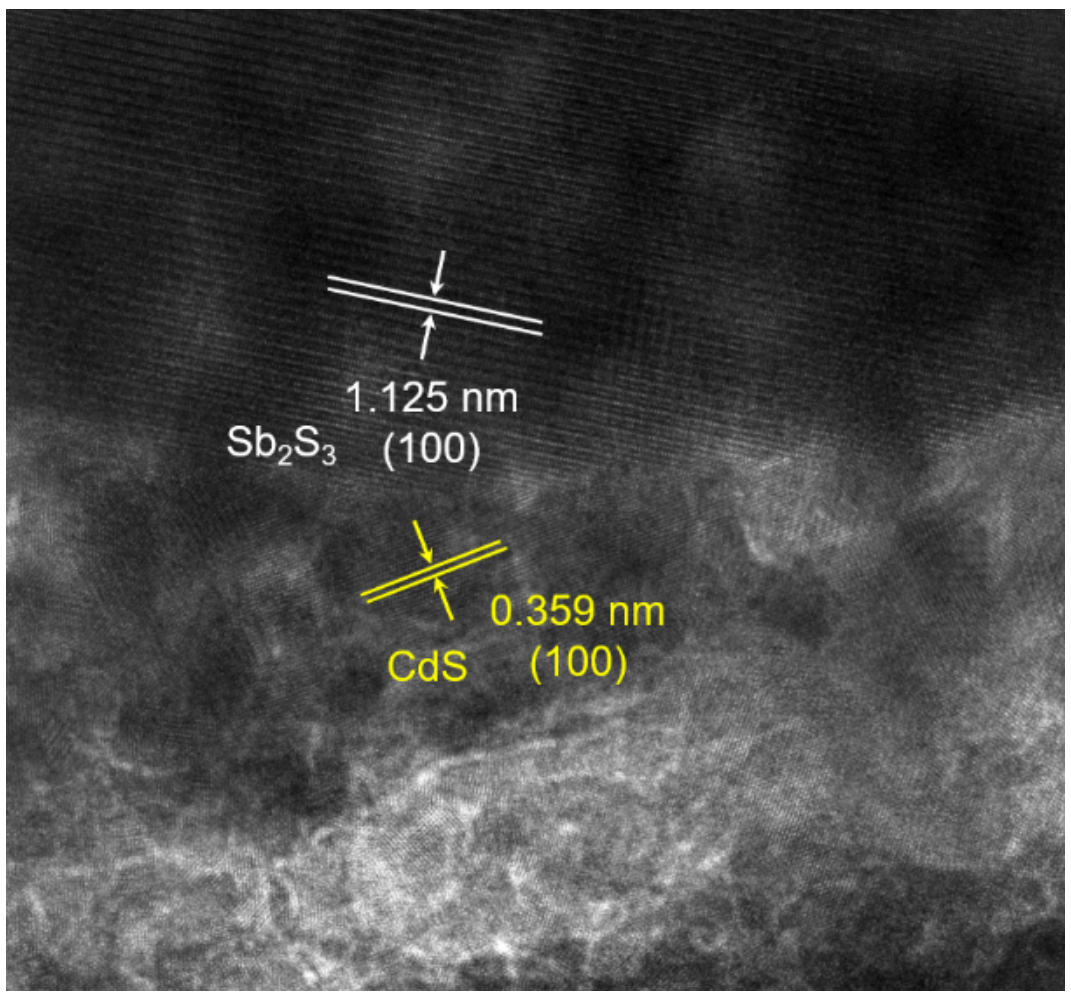

Supplementary Fig. 7 | The HRTEM image of the Sb_2S_3 film. The HRTEM image at the CdS/Sb_2S_3 interface of the Sb_2S_3 film sample. Scale bar, 20 nm.

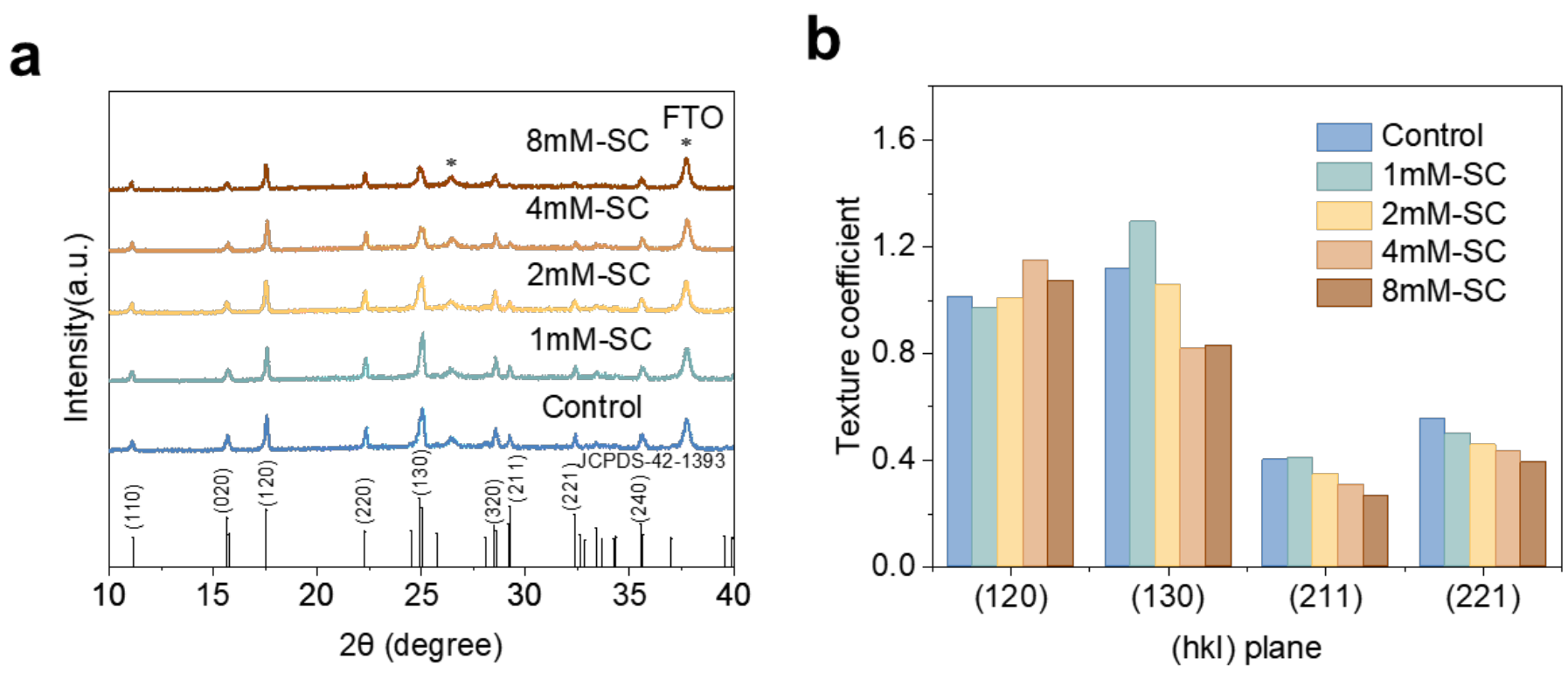

Supplementary Fig. 8 | X-ray diffraction (XRD) patterns and texture coefficients of Sb_2S_3 films. **a** XRD patterns of the control film and SC-Sb_2S_3 films obtained with the addition of different SC concentrations. The background peaks from FTO substrates are marked by the asterisks. **b** Texture coefficients of the (120), (130), (211), and (221) peaks, which dominate the diffraction patterns of Sb_2S_3 films.

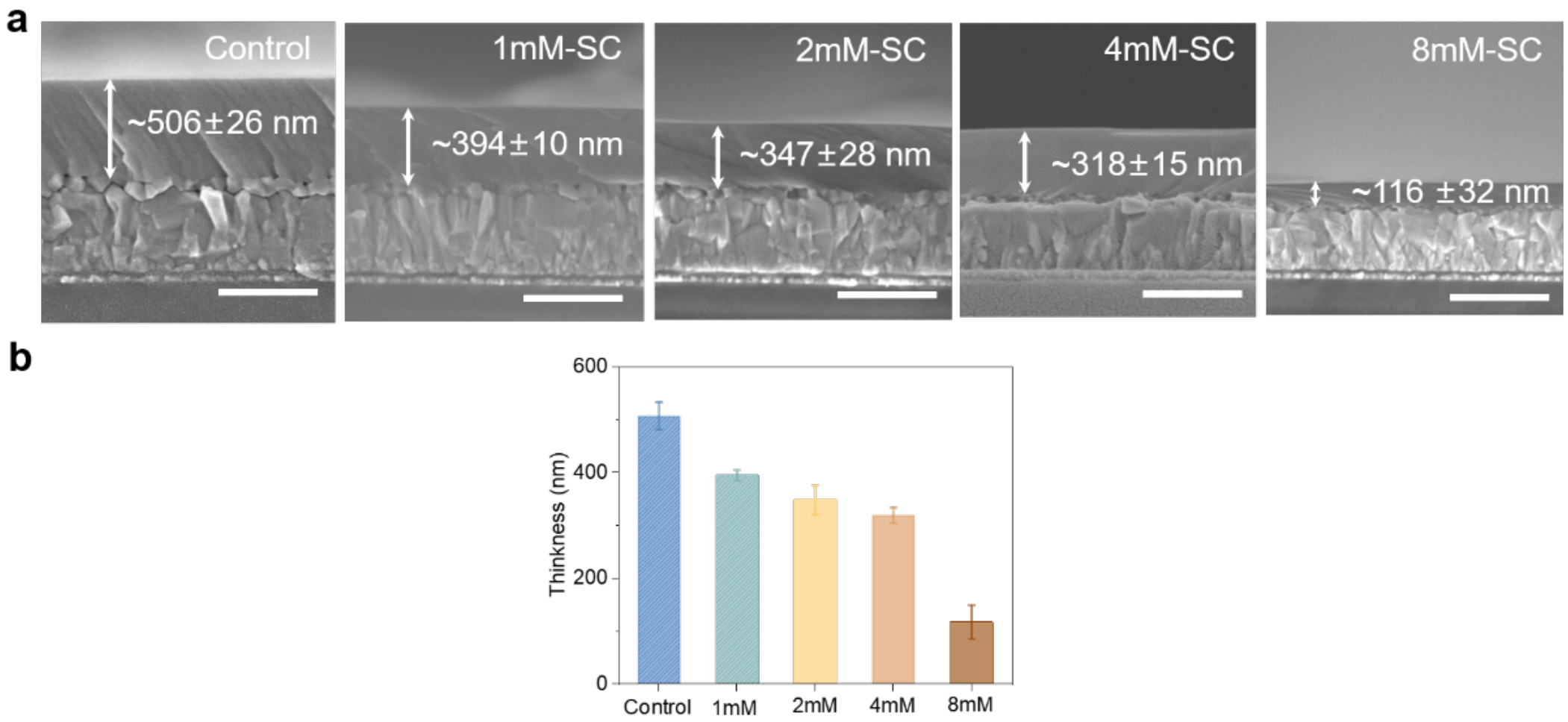

Supplementary Fig. 9 | The influence of SC concentrations on the thickness of the Sb_2S_3 film. **a** Cross-sectional SEM images of the control Sb_2S_3 films and SC-Sb_2S_3 films obtained with different concentrations of SC. Scale bar, 500 nm. The format of film thickness: average ± standard deviation. **b** Evolution of the thickness of Sb_2S_3 films obtained with the addition of different SC concentrations, and the error bars represent the standard deviation.

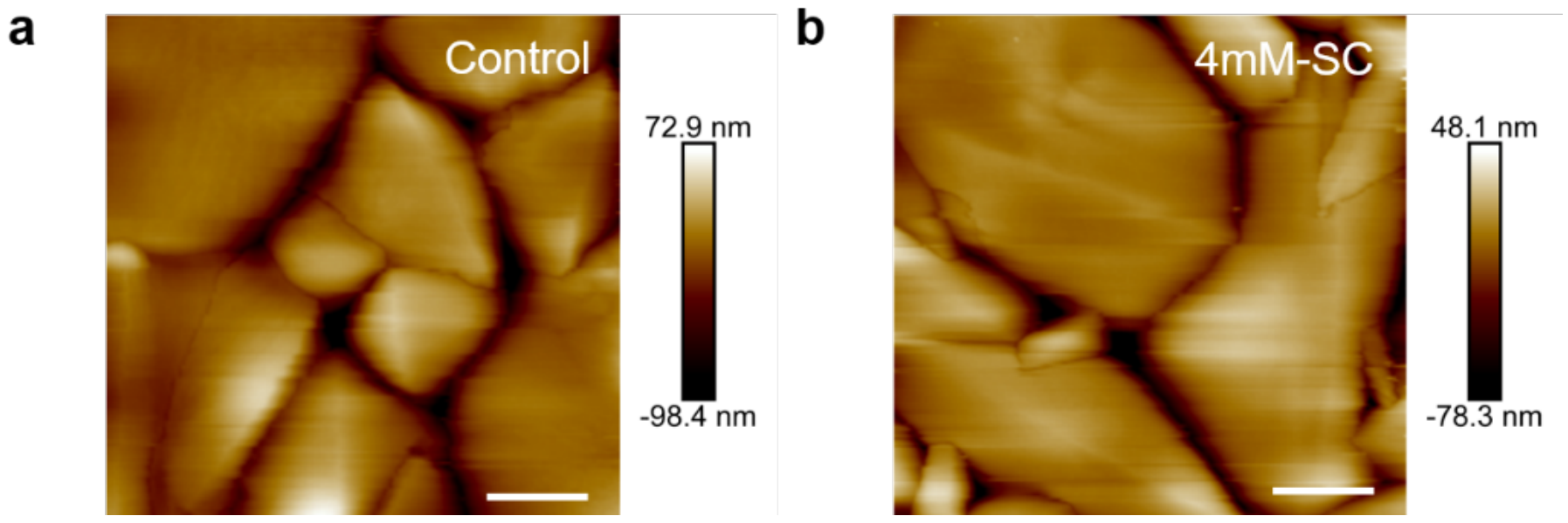

Supplementary Fig. 10 | Atomic force microscopy (AFM) maps of the Sb_2S_3 films. 2D topography spatial maps of **a** the control film sample and **b** 4mM-SC Sb_2S_3 film sample. Scale bar, 1 μm.

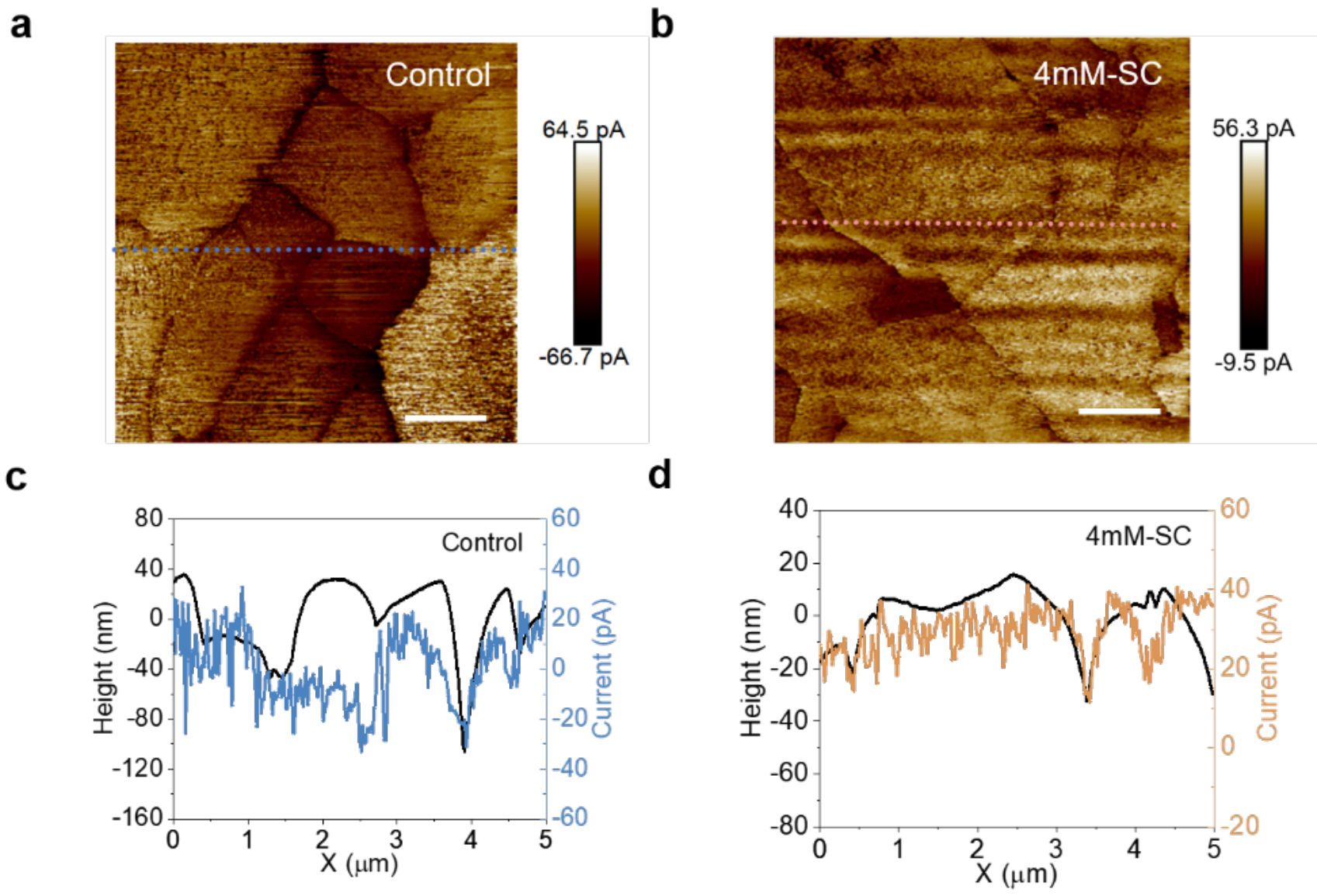

Supplementary Fig. 11 | Conductive-atomic force microscopy (c-AFM) images and the roughness and current maps of Sb_2S_3 films. a, b c-AFM images and **c, d** corresponding height profile and current analysis of the control film sample and 4mM-SC Sb_2S_3 film sample. The films suffer from evident current fluctuations at the GBs. Scale bar, 1 μm.

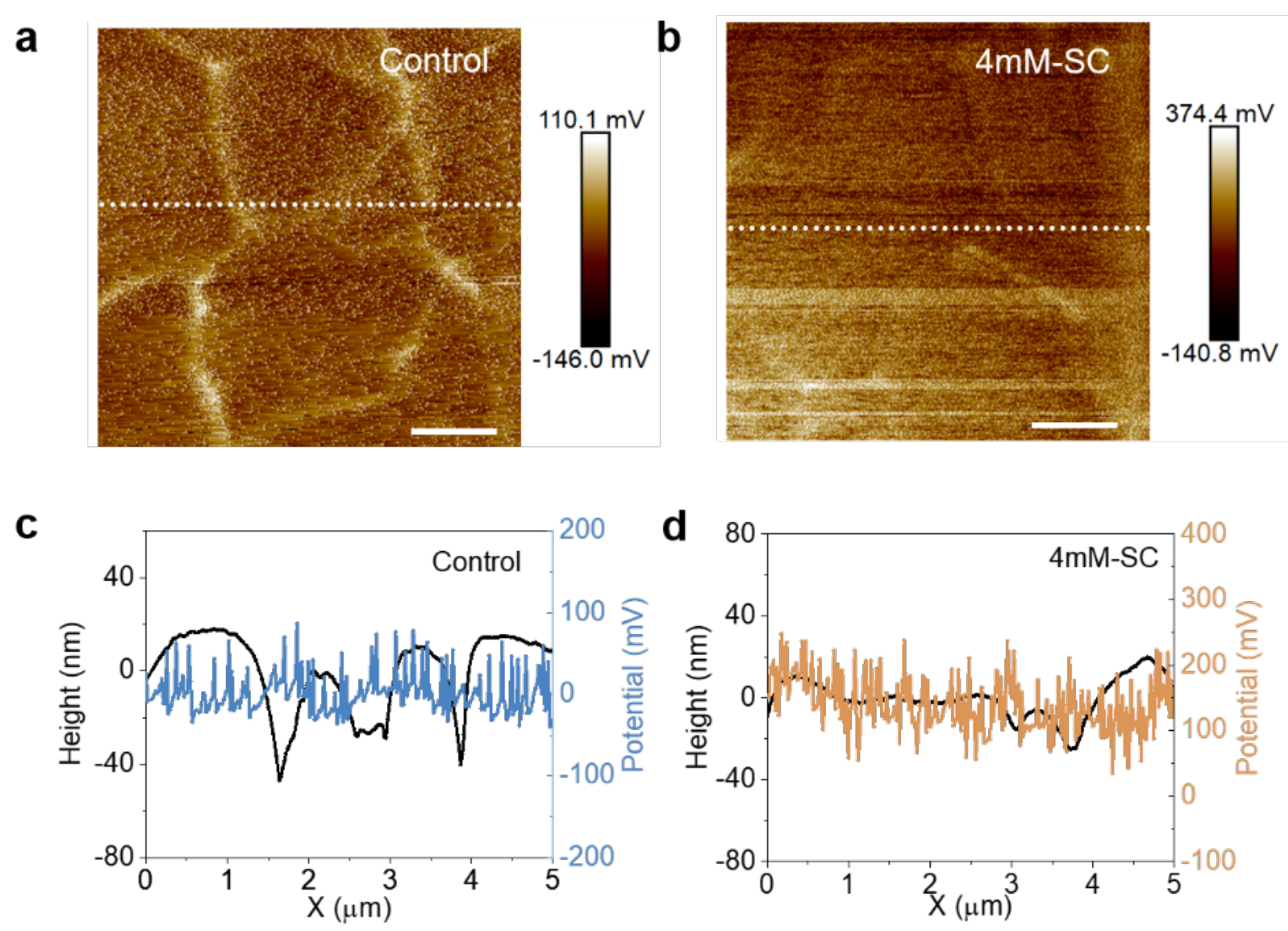

Supplementary Fig. 12 | Kelvin probe force microscopy (KPFM) to map the surface potential of Sb_2S_3 films. a, b KPFM images and **c, d** height profile and surface potentials of the control Sb_2S_3 and 4mM-SC Sb_2S_3 film sample. According to the testing principle of KPFM, the work function (WF) of sample (φ_{sample}) corresponds to the difference between the WF of tip (φ_{tip}) and the surface potential of sample (V_{surf}), i.e., $\varphi_{sample} = \varphi_{tip} - eV_{surf}$. Scale bar, 1 μm.

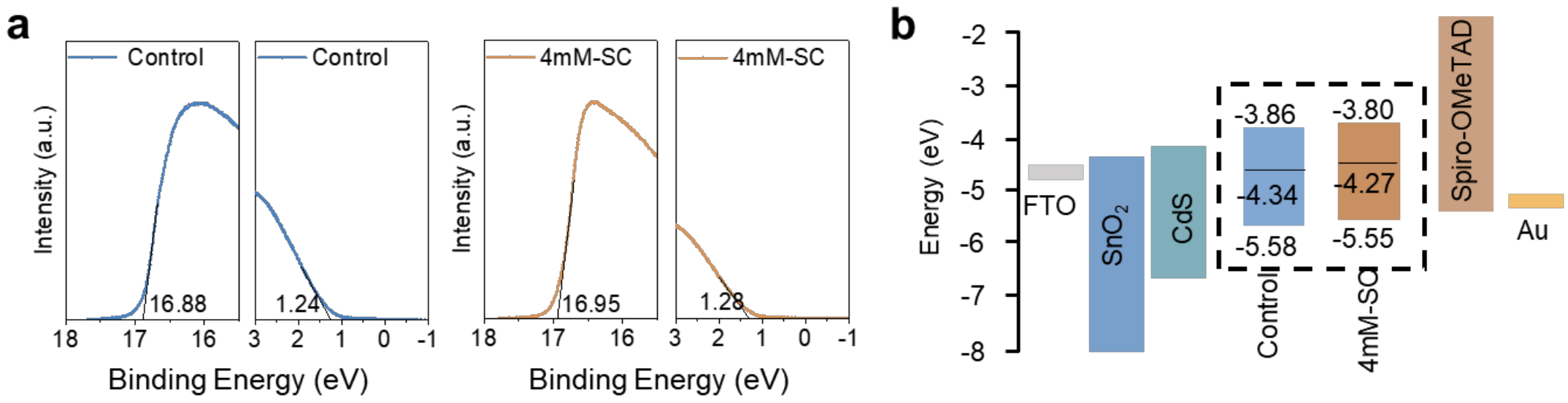

Supplementary Fig. 13 | UPS spectra of Sb_2S_3 films and the band structure of Sb_2S_3 solar cells. **a** UPS spectra of the control and 4mM-SC Sb_2S_3 film samples at the secondary electron cut-off and near the Fermi edge. **b** Schematic diagram of band structures of functional layers in the devices. The energy level of Sb_2S_3 were obtained from ultraviolet photoelectron spectroscopy (UPS) measurements. The VBM and CBM of Sb_2S_3 and 4mM-SC Sb_2S_3 films can be derived from the intersection of the tangent line and the coordinate axis at the secondary electron cut-off and near the Fermi edge. The cut-off binding energy (E_{cutoff}) values of Sb_2S_3 and 4mM-SC Sb_2S_3 are 16.88 and 16.95 eV, respectively. According to the following equation, the work function (WF) can be determined by $WF = E_{\mathrm{cutoff}} - 21.22$. The WF values relative to the Fermi level of Sb_2S_3 and 4mM-SC Sb_2S_3 are calculated to be −4.34 eV and −4.27 eV, respectively. The VBM relative to the Fermi level of control Sb_2S_3 and 4mM-SC Sb_2S_3 are −1.24 eV and −1.28 eV, respectively. Therefore, the VB positions (E_{VB}) relative to the vacuum level of control Sb_2S_3 and 4mM-SC Sb_2S_3 are calculated to be −5.58 and −5.55 eV, respectively, according to $E_{\mathrm{VB}} = WF - E_{\mathrm{F,edge}}$. Combined with the band gap value, the CB positions relative to the vacuum level of the control Sb_2S_3 and 4mM-SC Sb_2S_3 are −3.86 eV and −3.80 eV, respectively. The energy levels of other functional layers are compiled from the literature[16]. Here, the bandgap values of the control and SC-Sb_2S_3 films are determined to be 1.72 and 1.75 eV, respectively, based on the Tauc plots of UV-vis-NIR absorption spectra (Supplementary Fig. 14). As shown, the energy levels of Sb_2S_3 match well with the ETL (CdS) and HTL (Spiro-OMeTAD), favoring for the efficient charge transport from absorber layer towards two electrodes. Moreover, compared to the control Sb_2S_3 film, the Fermi level of SC-Sb_2S_3 is shifted up towards CBM, indicating an increased electron carrier concentration for n-type Sb_2S_3. The upward shift of CBM for the 4mM-SC film facilitates the photogenerated carrier transport from the absorber layer to the ETL.

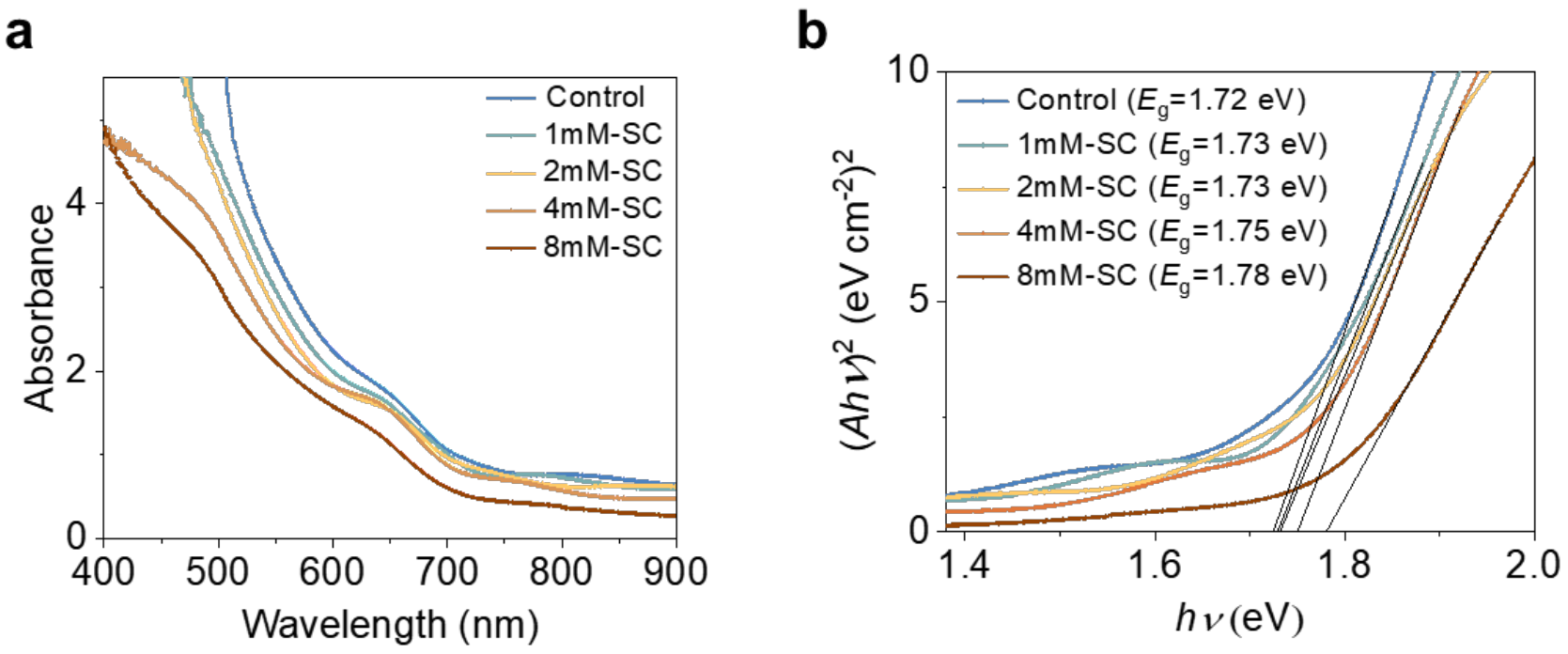

Supplementary Fig. 14 | Absorption spectra and corresponding Tauc plots of Sb_2S_3 films. a UV-vis-NIR absorption spectra and **b** corresponding Tauc plots of the control and SC-Sb_2S_3 films obtained with the addition of different SC concentrations. The bandgaps of Sb_2S_3 films are determined by the Tauc plots of UV-vis-NIR absorption spectra. The bandgap values of the control, 1mM, 2mM, 4mM, 8mM SC-Sb_2S_3 films are estimated to be 1.72, 1.73, 1.73, 1.75, and 1.78 eV, respectively.

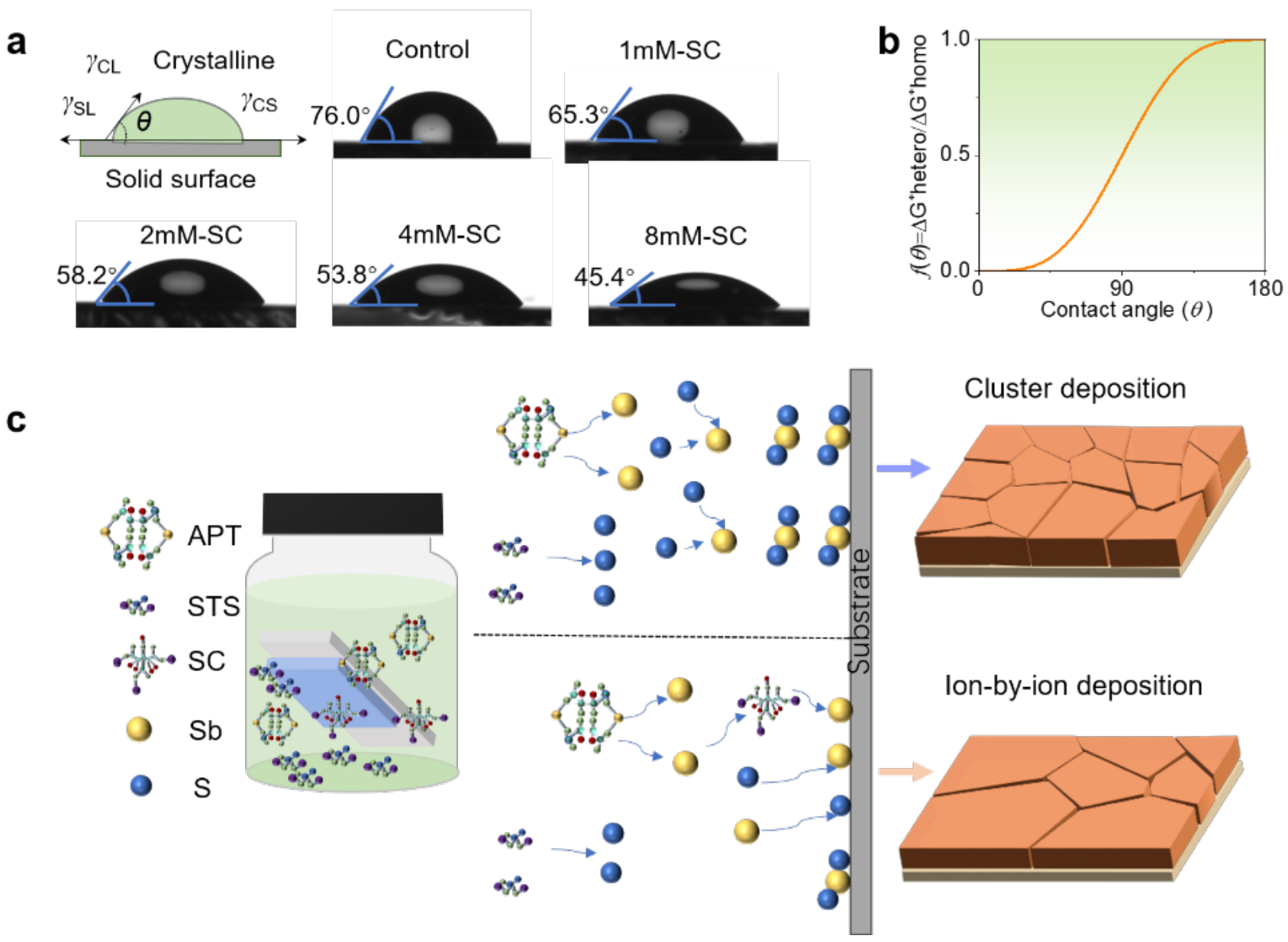

Supplementary Fig. 15 | Deposition mechanism of Sb_2S_3 solar cells with the incorporation of SC. a Schematic of the contact angle (θ) for the heterogeneous nucleation of films, and the water contact angles of the pristine CdS substrate, as well as the CdS substrate treated by precursor solutions with different SC concentrations for 45 min CBD. **b** Ratio of the free energies of homogeneous and heterogeneous nucleation as a function of the contact angle. **c** Illustration of the cluster deposition and SC-mediated ion-by-ion deposition of Sb_2S_3 thin films.

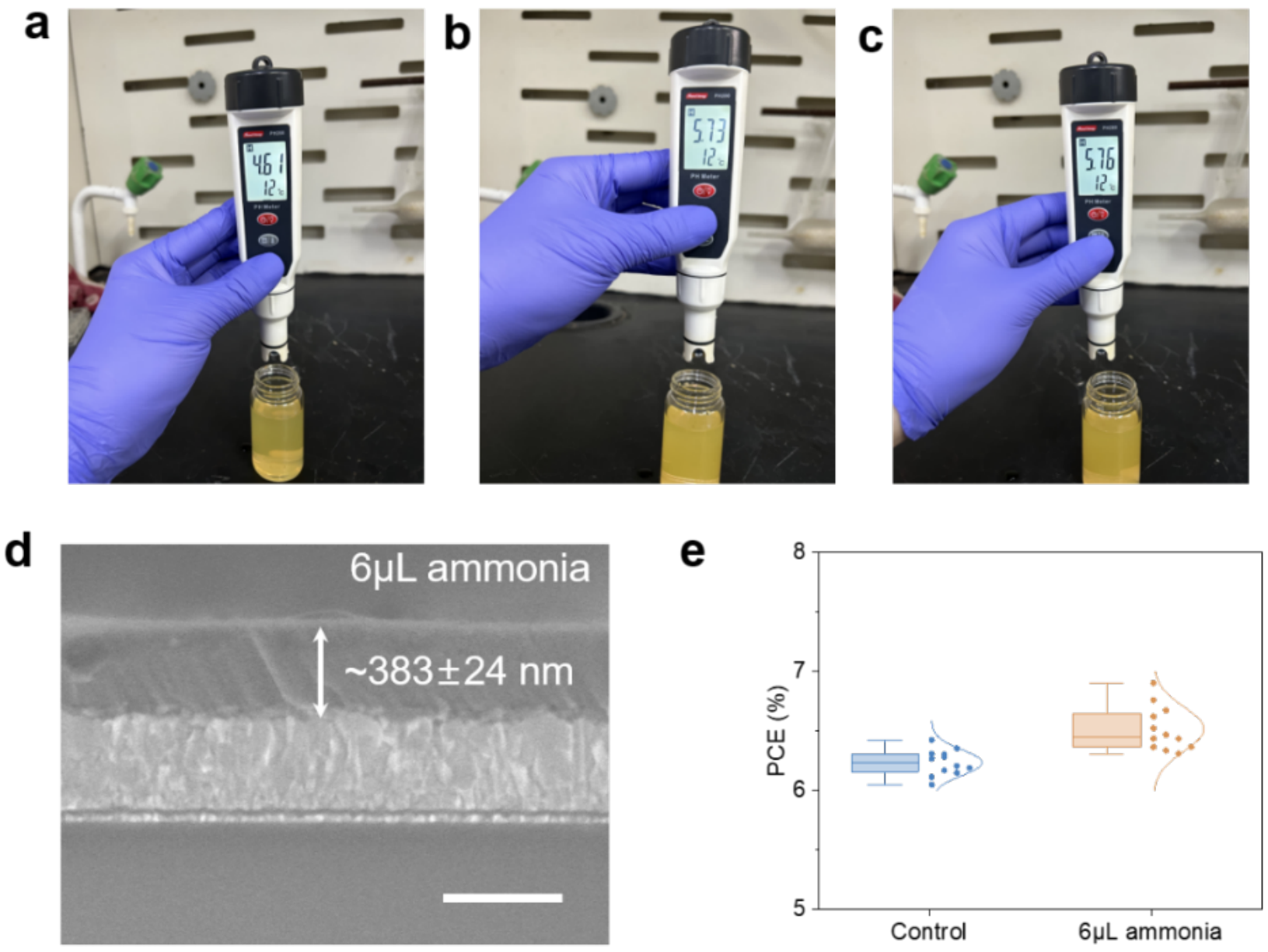

Supplementary Fig. 16 | The influence of precursor pH on the film deposition and device performance. pH of precursor solutions **a** without the additive, **b** with the addition of 4mM-SC, and **c** with the addition of 6μL ammonia solution. **d** Cross-sectional SEM image of Sb_2S_3 thin film prepared with 6 μL ammonia solution as an additive. Scale bar, 500 nm. The format of film thickness: average ± standard deviation. **e** The statistics of PCEs of the control Sb_2S_3 solar cells and the devices prepared with the use of ammonia additive. 12 devices were measured for each type of device, and the performance metrics of each device are shown as individual data points. The statistical distribution for the PCE, V_{OC}, J_{SC}, and FF were obtained from 12 devices, and the lower whisker, lower box edge, middle line, upper box edge, and upper whisker refer to minimum, 25th percentile, median, 75th percentile, and maximum of the dataset, respectively.

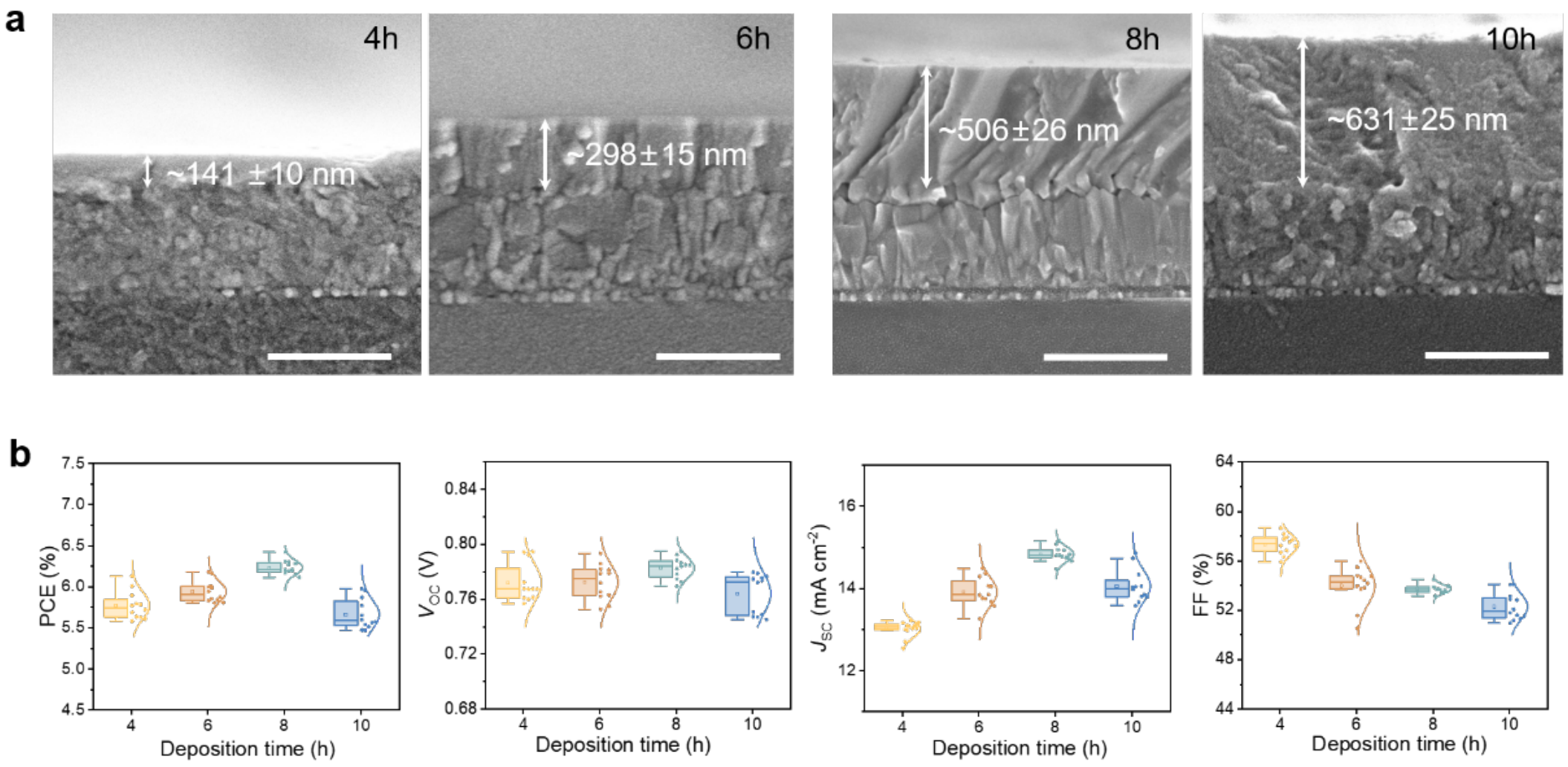

Supplementary Fig. 17 | The influence of CBD time on the film thickness and device performance. a Cross-sectional SEM images of the control Sb_2S_3 films obtained with different CBD time (4 h, 6 h, 8 h and 10 h). Scale bar, 500 nm. The format of film thickness: average ± standard deviation. **b** The statistics of the device performance of control Sb_2S_3 solar cells obtained with different CBD time. 12 devices were measured for each type of device, and the performance metrics of each device are shown as individual data points. The statistical distribution for the PCE, V_{OC}, J_{SC}, and FF were obtained from 12 devices, and the lower whisker, lower box edge, middle line, upper box edge, and upper whisker refer to minimum, 25th percentile, median, 75th percentile, and maximum of the dataset, respectively. The results show that the control sample obtained with the 8 h CBD delivers the highest PCE, implying that the optimal device performance of the control sample is much lower than that of the SC-Sb_2S_3 samples.

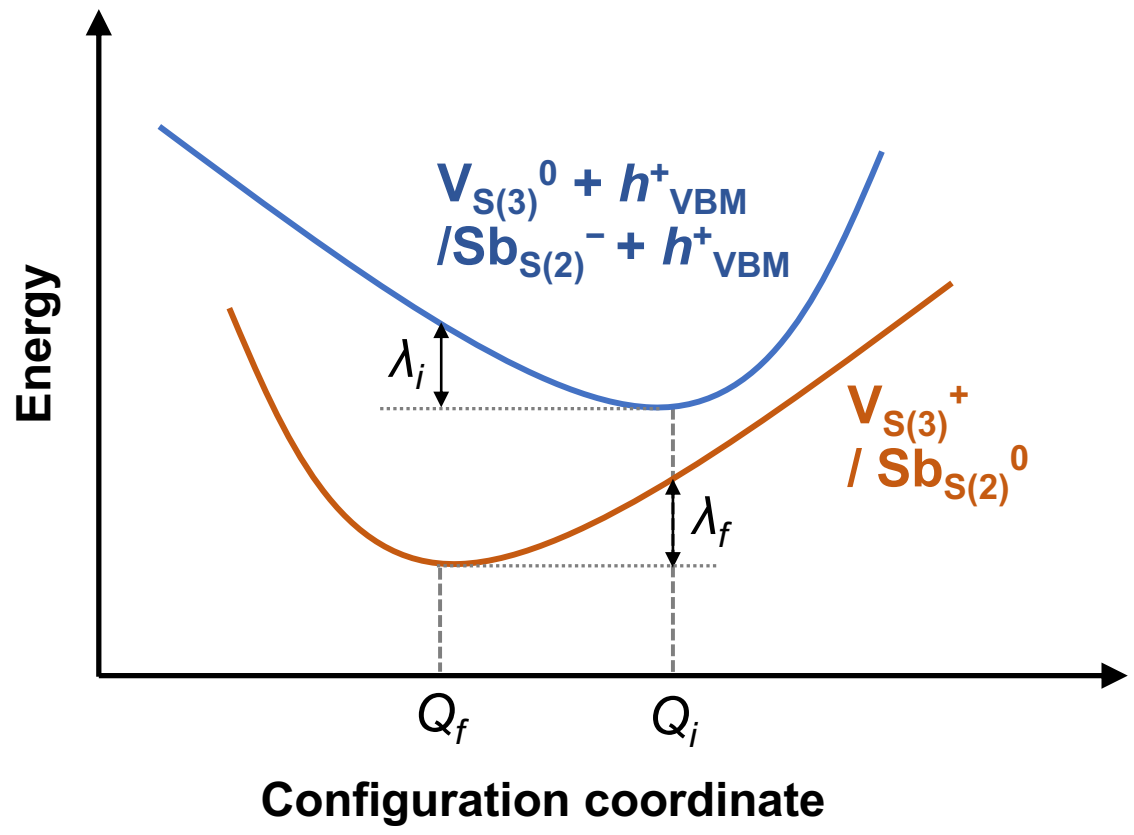

Supplementary Fig. 18 | Schematic defining the Franck-Condon relaxation energies. The Franck-Condon relaxation energies λ_i and λ_f for the hole-capture transitions $V_{S(3)}{}^{0} + h^{+}{}_{VBM} \rightarrow V_{S(3)}{}^{+}$ and $Sb_{S(2)}{}^{-} + h^{+}{}_{VBM} \rightarrow Sb_{S(2)}{}^{0}$ shown in Figure 5d,e.

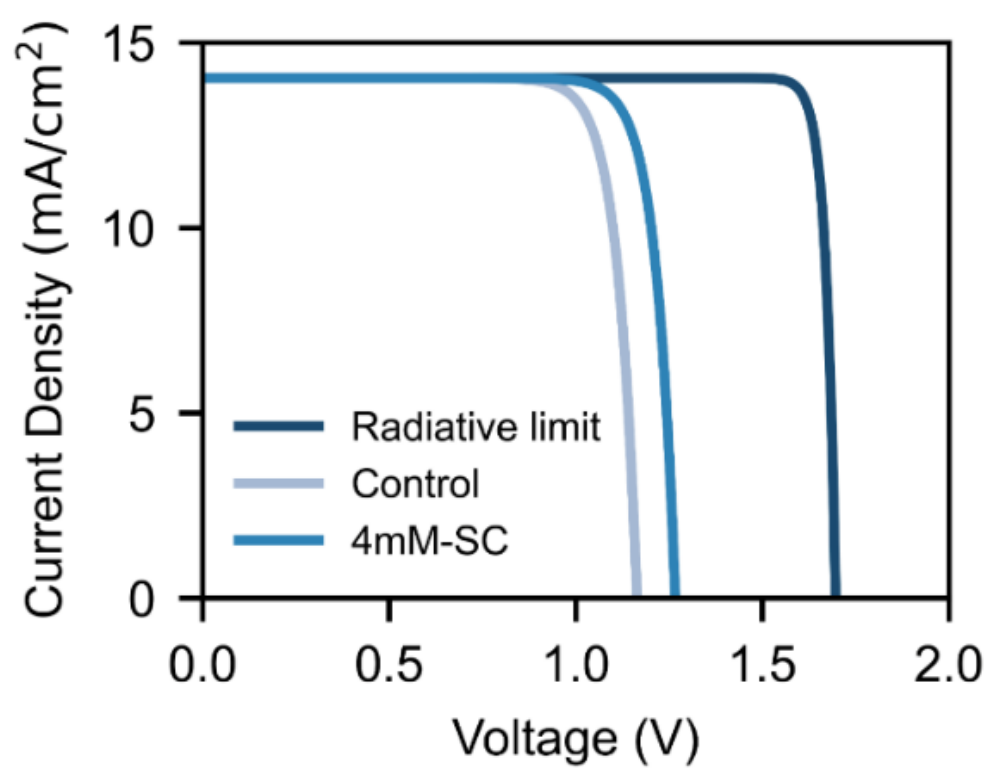

Supplementary Fig. 19 | Calculated *J-V* curves for Sb_2S_3 solar cells under the radiative limit and incorporating defect-mediated non-radiative recombination. The simulations use experimentally measured defect levels, defect densities and capture cross sections of "Control" and "4mM-SC" samples as inputs.

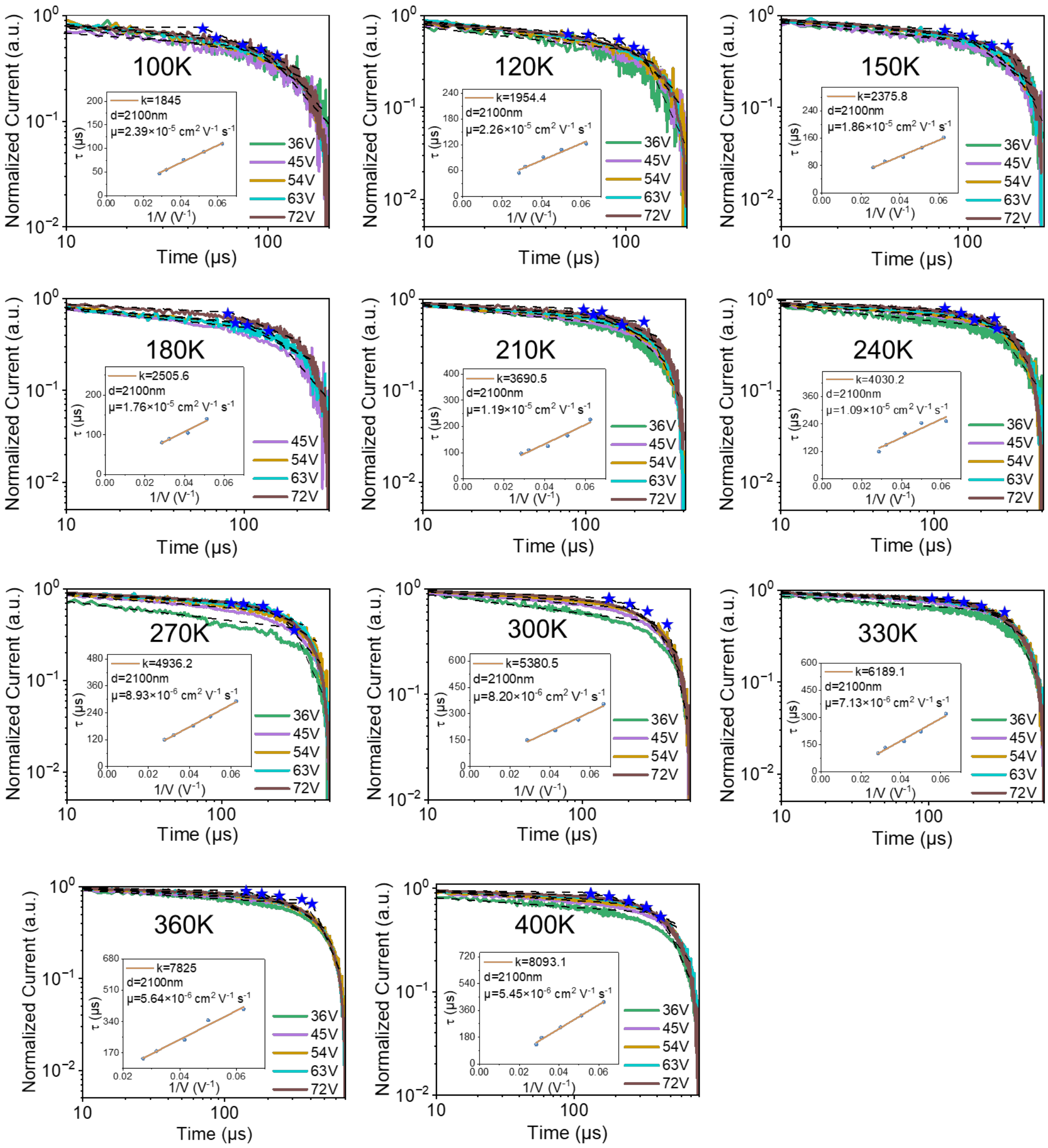

Supplementary Fig. 20 | TOF measurements of Sb_2S_3 films. The transient currents in TOF measurements of Sb_2S_3 films after photoexcitation at time t = 0 in a bilogarithmic plot. The transit time τ_t is defined by the turning point in the bilogarithmic plot of photocurrent versus decay time.

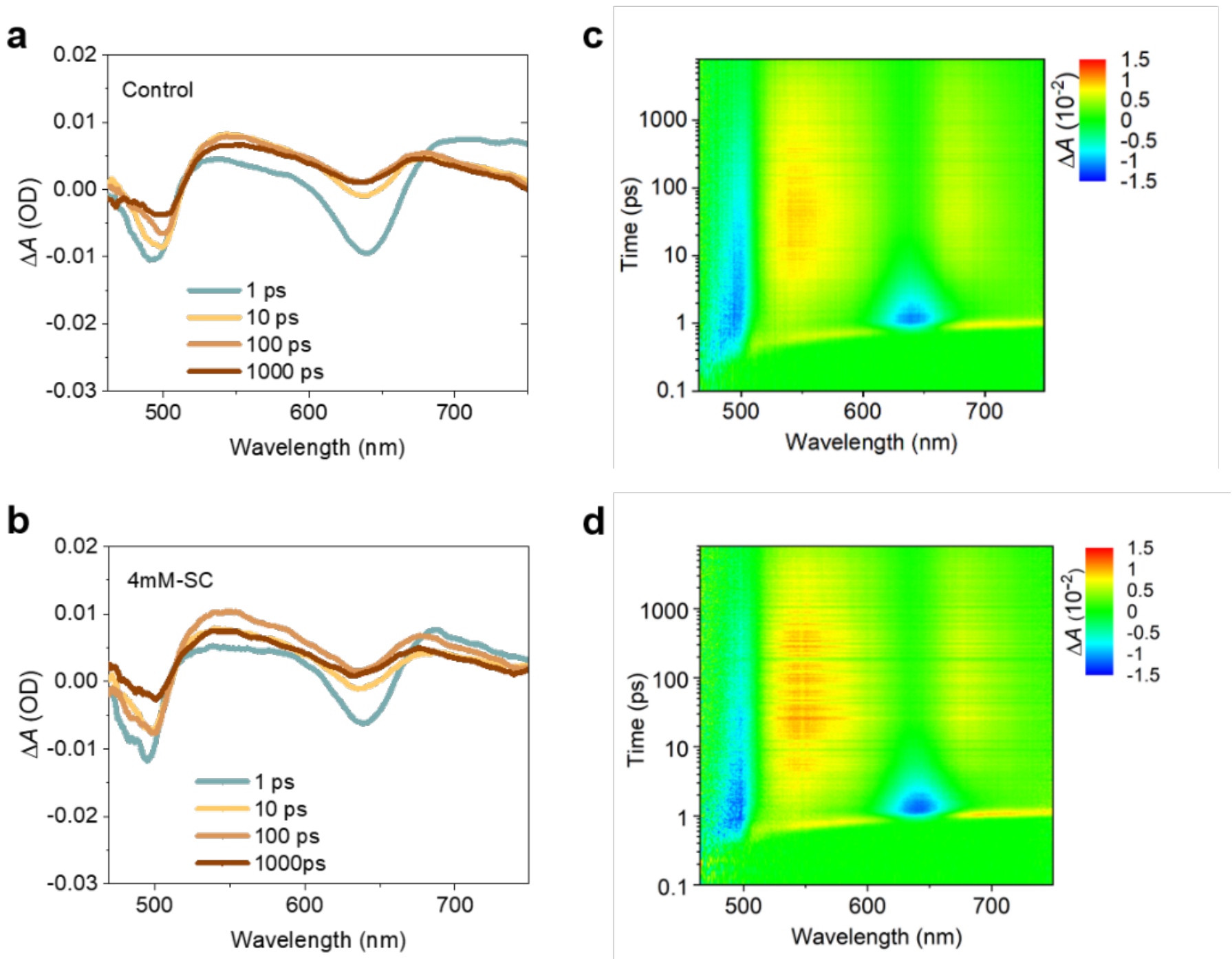

Supplementary Fig. 21 | Transient absorption (TA) spectra and 2D TA spectroscopy pseudo-color images of Sb_2S_3 films. TA spectra obtained at 1, 10, 100, 1000, and 5000 ps pump-probe delay for **a** control and **b** 4mM-SC Sb_2S_3 film samples. Excitation was with a 400 nm wavelength pulsed laser at a fluence of 251 μJ cm^{-2} $pulse^{-1}$ and a repetition rate of 1000 Hz. 2D TA spectroscopy pseudo-color images for time-resolved absorption spectra of **c** control Sb_2S_3 and **d** 4mM-SC Sb_2S_3 film samples.

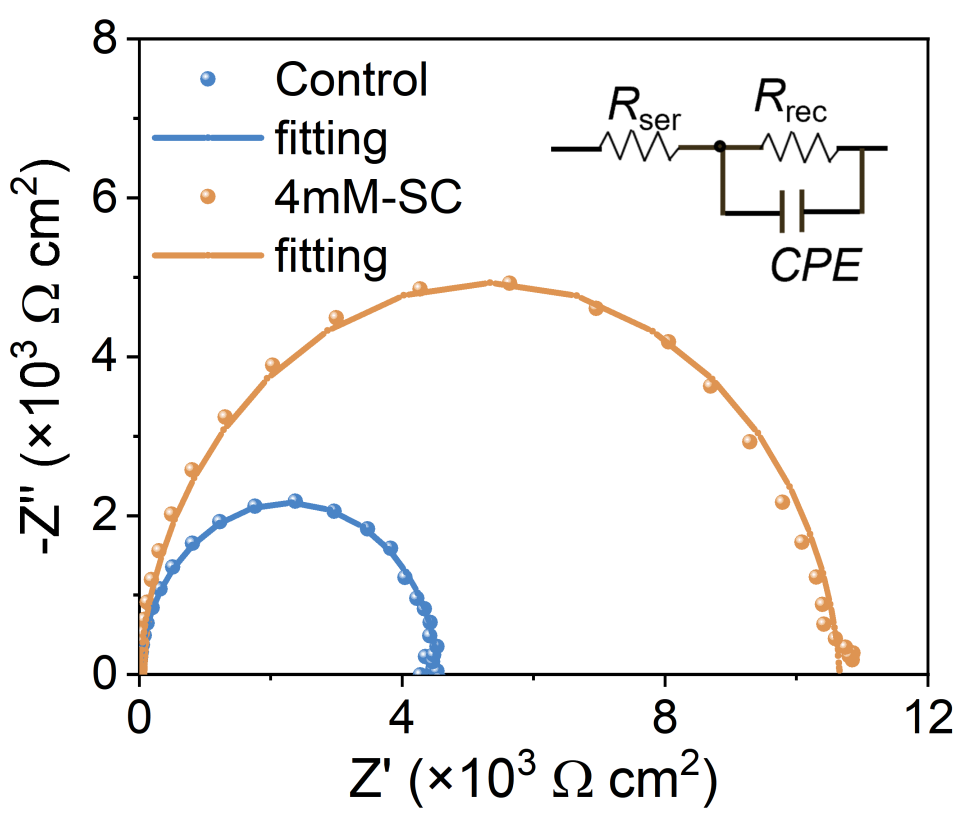

Supplementary Fig. 22 | Nyquist plots of Sb_2S_3 films. Nyquist plots for the control and 4mM-SC devices measured in the dark, with the equivalent circuit used for fitting shown in the inset.

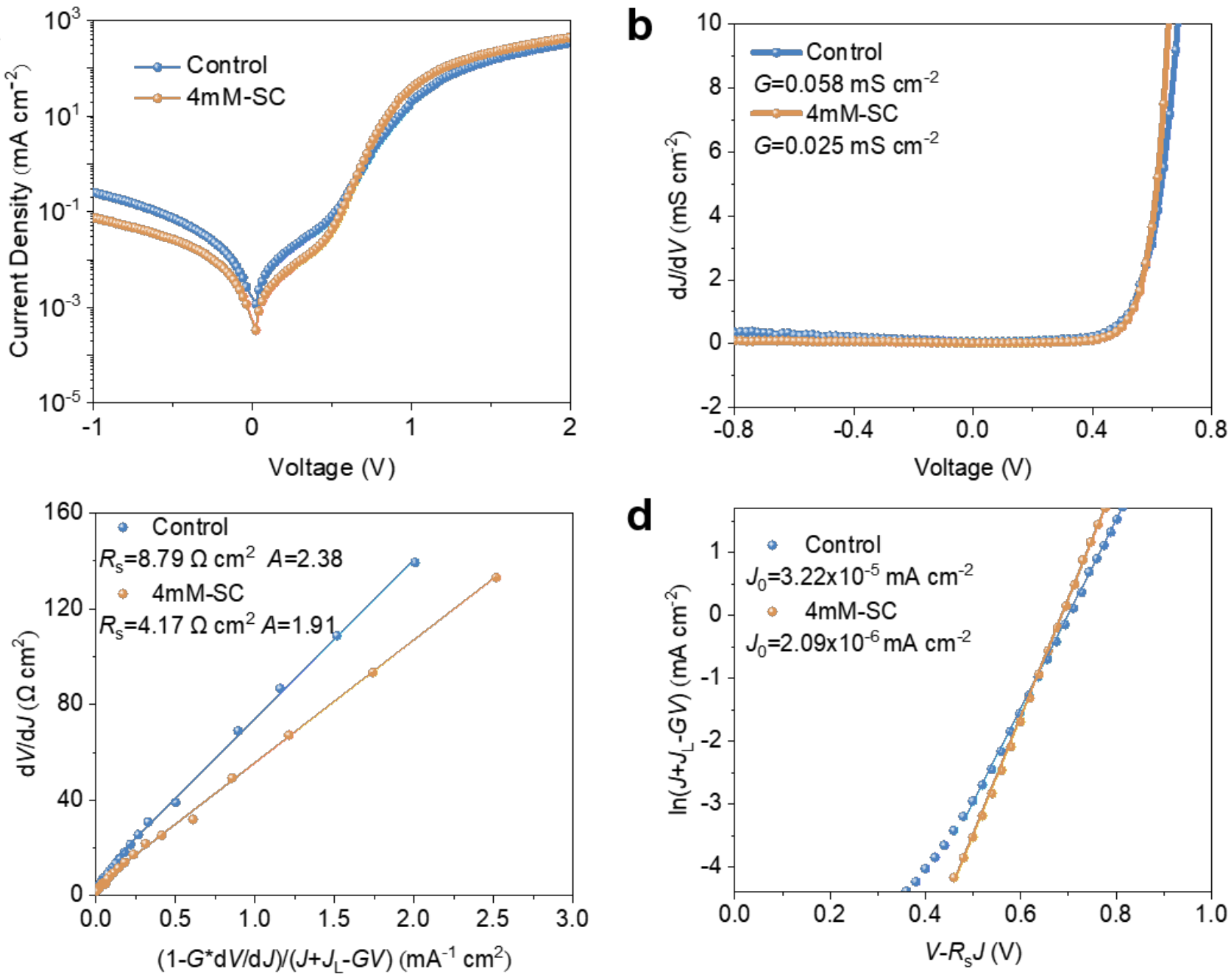

Supplementary Fig. 23 | The analysis of *J-V* curves of Sb_2S_3 solar cells measured under dark. **a** The *J-V* curves measured under dark for the control and 4mM-SC Sb_2S_3 solar cells, and corresponding fitting results of **b** the shunt conductance G, **c** dV/dJ versus $(1-G*dV/dJ)/(J+J_L-GV)$ relationship curves for determining R and A, and **d** $\ln(J+J_L-GV)$ versus $V-R_SJ$ relationship curves employed to determine J_0.

Supplementary Table 1. Summary of performance parameters of previously reported Sb_2S_3 solar cells with competitive PCEs and well-developed V_{OC}.

**Device structure**	**Fabrication method**	**V_{OC} (mV)**	**J_{SC} (mA cm^{-2})**	**FF (%)**	**PCE (%)**	**Year/ Ref.**
FTO/[a]TiO_2/Sb_2S_3/CuSCN/Au (Sensitized)	[b]CBD	490	14.10	48.80	3.37	2009[17]
FTO/TiO_2/Sb_2S_3/Spiro/Au (Sensitized)	CBD	556	12.30	69.90	5.06	2010[18]
FTO/TiO_2/Sb_2S_3/PCDTBT/PTAA/Au (Sensitized)	CBD	616	15.30	65.70	6.18	2011[19]
FTO/TiO_2/Sb_2S_3/P3HT/PCBM/Au (Sensitized)	CBD	595	16.00	65.50	6.30	2012[20]
FTO/[c]TiO_2/Sb_2S_3/P3HT/Au (Planar)	CBD	560	7.15	35.00	1.43	2012[21]
FTO/TiO_2/Sb_2S_3/CuSCN/Au (Sensitized)	CBD	700	15.00	53.00	5.66	2013[22]
ITO/TiO_2/Sb_2S_3/P3HT/HIL/Au (Planar)	CBD	670	9.80	39.00	2.56	2013[23]

FTO/TiO_2/Sb_2S_3/PCPDTBT/PEDOT:PSS/Au (Sensitized)	CBD	711	16.10	65.00	7.50	2014[24]
FTO/TiO_2/Sb_2S_3/P3HT/Au (Planar)	[d]ALD	667	14.92	58.04	5.77	2014[25]
FTO/TiO_2/Sb_2S_3/P3HT/Au (Sensitized)	[e]SC	616	8.12	45.90	2.30	2015[26]
ITO/TiO_2/Sb_2S_3/PCPDTBT/Ag (Planar)	CBD	726	11.00	50.5	4.06	2015[27]
FTO/TiO_2/Sb_2S_3/P3HT/PEDOT:PSS/Ag (Sensitized)	CBD	500	18.90	40.00	3.80	2016[28]
ITO/SnO_2/CdS/Sb_2S_3/Au (Planar)	[f]RTE	710	10.80	47.50	3.50	2016[29]
ITO/CdS/Sb_2S_3/Au (Planar)	RTE	714	11.39	51.22	4.17	2017[30]
FTO/TiO_2/Sb_2S_3/Spiro/Au (Sensitized)	[g]VASP	671	18.43	54.80	6.78	2018[31]
FTO/TiO_2/Sb_2S_3/Spiro/Au (Planar)	CBD	690	13.37	49.72	4.61	2018[32]
FTO/TiO_2/Sb_2S_3/Spiro/Au (Sensitized)	RTE	710	16.11	50.72	5.80	2019[33]
FTO/TiO_2/Sb_2S_3/Spiro/Au (Planar)	SC	690	17.30	55.18	6.56	2019[34]
FTO/TiO_2/Sb_2S_3/SbSI/PCPDTBT/PEDOT:PSS/Au (Sensitized)	CBD	620	14.92	66	6.08	2020[35]
FTO/TiO_2/CdS/Sb_2S_3/Spiro/Au (Planar)	[h]HT	760	15.11	56.06	6.40	2020[36]
FTO/TiO_2/Sb_2S_3/Spiro/Au (Sensitized)	SC	530	18.06	60.28	5.77	2021[37]
FTO/TiO_2/CdS/Sb_2S_3/Spiro/Au (Planar)	HT	748	15.29	57.07	6.53	2021[38]
FTO/CdS/Sb_2S_3/Spiro/Au (Planar)	CBD	757	17.41	60.48	8.00	2022[7]
FTO/CdS/Sb_2S_3/Spiro-OMeTAD/Au (Planar)	HT	770	14.38	58.53	6.51	2023[8]
FTO/SnO_2/CdS/Sb_2S_3/Spiro-OMeTAD/Au (Planar)	HT	805	12.79	63.71	6.56	2023[39]
FTO/SnO_2/CdS/Sb_2S_3/Spiro-OMeTAD/Au (Planar)	HT	796	17.18	58.28	7.66	2024[16]
FTO/SnO_2/CdS/Sb_2S_3/PbS-EDT /Au (Planar)	HT	712	17.91	64.81	8.26	2025[40]
FTO/TiO_2/CdS/Sb_2S_3/Spiro-OMeTAD/MoO_x/Ag (Planar)	HT	811.5	15.25	55.17	6.83	2025[41]
FTO/SnO_2/CdS/Sb_2S_3/Spiro-OMeTAD/Au	CBD	817	16.44	57.07	7.67	This work
(Planar)		824	16.36	56.52	7.62	This work

Note:

[a] TiO_2 in sensitized device represent compact-TiO_2/mesoporous-TiO_2;

[b] CBD: chemical bath deposition;

[c] TiO_2 in planar device represent compact-TiO_2;

[d] ALD: atomic layer deposition;

[e]SC: spin-coating;

[f]RTE: rapid thermal evaporation;

[g]VASP: vacuum assisted solution process

[h]HT: hydrothermal deposition

Supplementary Table 2. Summary of PL characteristics of kinds of Sb_2S_3 samples obtained from different methods in the literature.

No.	Samples	Deposition methods	PL peak (eV)	E_g (eV)	Red shift (eV)	Excitation	Ref.
1	FTO/CdS/Sb_2S_3 films	HT[a]	1.15	1.65	~0.5	CW 532 nm laser	42
1	Single-crystal flake	CVT[b]	1.1	1.7	~0.6	CW 532 nm laser	42
2	glass/Sb_2S_3 films	SC[c]	~ 1.71, Symmetrical	1.73	~ 0.02	CW 405 nm laser	43
3	FTO/CdS/Sb_2S_3 films	RTE[d]	~ 1.63, Symmetrical	1.64	~ 0.01	532 nm pumped laser	44
4	FTO/CdS/Sb_2S_3 films	HT[a]	~ 1.63, Symmetrical	1.69	~ 0.06	532 nm laser	45
5	glass/FTO/CdS/ Sb_2S_3 films	CSS	1.72, unsymmetrical	1.8	0.08	266 nm laser	46
6	FTO/CdS/Sb_2S_3 films	HT[a]	~ 1.64, Symmetrical	1.77	~ 0.13	532 nm laser	47
7	FTO/TiO_2/CdS/ Sb_2S_3 films	SC[c]	~ 1.71, Unsymmetrical	1.87	~ 0.16	unmentioned	48
8	FTO/SnO_2/CdS/ Sb_2S_3 films	HT[a]	1.64, Symmetrical	1.63	~ 0	532 nm laser	49

9	FTO/CdS/Sb_2S_3 films	HT[a]	~ 1.70, Symmetrical	1.72	~ 0.02	unmentioned	41
10	FTO/TiO_2/Sb_2S_3 films	CBD[e]	~ 1.69, Unsymmetrical	1.86	~ 0.17	unmentioned	50
11	SLG/Mo/Sb_2S_3 films	VTD[f]	1.64, Symmetrical	1.72	0.08	unmentioned	51
12	glass/ITO/Sb_2S_3 films	Sol-gel	~ 1.68, Symmetrical	1.68	~ 0	638 nm laser	52
13	Glass/Mo/Sb_2S_3 films	Sputtering	1.664, Symmetrical	1.778	0.114	532 nm laser	53
14	ITO/Sb_2S_3 films	SC[c]	1.78, Symmetrical	1.78	0	266 nm laser	54
15	Mo/Sb_2S_3 films	EBE[g]	~ 1.44 eV Unsymmetrical	1.70	~ 0.26	510 nm laser, 50 K	55
16	Au-coated ITO/ Sb_2S_3 films	TE[g]	~ 1.73 eV Symmetrical Weak	1.76	~ 0.03	unmentioned	56
17	quartz/Sb_2S_3 films	SC[c]	1.682 eV Symmetrical Weak	1.71	0.028	510 nm pulsed laser	57
18	Sb_2S_3 nanoparticles (~10 nm)	Hot-injection	1.88, Unsymmetrical	1.93	0.05	633 nm laser	58
19	Si/SiO_2/ single-crystalline Sb_2S_3 rods	HT[a]	1.67, Symmetrical	1.61	~ 0	532 nm laser	59
20	FTO/Sb_2S_3 nanorod arrays	SC[c]	1.65, Symmetrical	1.65	0	371 nm laser	60

Note:

[a] HT: hydrothermal deposition;

[b] CVT: Chemical vapor transport;

[c] SC: Spin-coating;

[d] RTE: rapid thermal evaporation;

[e] CBD: Chemical bath deposition;

[f] VTD: vapor transport deposition;

[g] EBE: Electron-beam evaporation

[h] TE: Thermal evaporation

Supplementary Table 3. Indoor photovoltaic performance of 4mM-SC Sb_2S_3 solar cells measured under WLED (3000 K) at 1000, 500, and 200 lux.

4mM-SC device	V_{OC} (mV)	J_{SC} (mA cm^{-2})	FF (%)	PCE (%)	R_S (kΩ cm^2)	R_{SH} (kΩ cm^2)
1-sun	817	16.44	57.07	7.67	0.008	0.28
1000 lux LED	626	0.137	63.17	18.56	0.41	46.51
500 lux LED	599	0.067	62.94	17.40	0.91	88.47
200 lux LED	565	0.029	62.69	16.57	2.05	208.05

Supplementary Table 4. Raw data of defect levels for Sb_2S_3 obtained from DFT calculations. Note: Charge states with * refer to thermodynamically metastable states. The unit of values is eV.

	**$V_{Sb(1)}$**	**$V_{Sb(2)}$**
3 –> 2*	0.413	0.070
2* –> 1	−0.037	−0.033
1 –> 0*	1.387	1.488
0* –> −1*	0.433	0.073
−1* –> −2*	1.205	1.205
−2* –> −3	−0.026	0.227

	**$S_{Sb(1)}$**		**$S_{Sb(2)}$**
3 –> 2*	0.604	3 –> 2	−0.054

2* −> 1	−0.030	2 −> 1	−0.019
1 −> 0*	0.697	1 −> 0*	0.803
0* −> −1	0.372	0* −> −1	0.490
−1 −> −2	1.732	−1 −> −2	1.597

	$\mathbf{V_{S(1)}}$	$\mathbf{V_{S(3)}}$		$\mathbf{V_{S(2)}}$
2 −> 1*	1.231	1.432	2 −> 1*	1.180
1* −> 0	0.597	0.594	1* −> 0*	1.069
0 −> −1*	1.709	1.903	0* −> −1*	1.359
−1* −> −2	0.621	0.451	−1* −> −2	0.727

	$\mathbf{Sb_{S(1)}}$		$\mathbf{Sb_{S(2)}}$	$\mathbf{Sb_{S(3)}}$
3 −> 2*	1.254	3 −> 2*	0.482	0.756
2* −> 1 *	0.604	2* −> 1	0.207	0.388
1* −> 0*	0.451	1 −> 0*	1.138	1.007
0* −> −1	0.564	0* −> −1	0.607	0.409
−1 −> −2	1.748	−1 −> −2	1.748	1.840

Supplementary Table 5. Defect identification of DLTS experiment results using DFT calculations. According to first-principles calculations, those defects that do not act as traps for either electrons or holes are labeled using "--".

	**Trap**	$\mathbf{E_T}$ **(eV)**	**Trap type**	**σ (cm²)**
EXP-H1	H1	$E_V + 0.483$	Hole	2.08×10^{-17}
EXP-H2	H2	$E_V + 0.635$	Hole	6.62×10^{-16}
DFT	$V_{Sb(1)}^{\ 0\leftrightarrow-1}$	$E_V + 0.433$	--	--
	$V_{S(3)}^{\ -2\leftrightarrow-1}$	$E_V + 0.451$	--	--
	$Sb_{S(1)}^{\ +1\leftrightarrow0}$	$E_V + 0.451$	--	--
	$Sb_{S(2)}^{\ +3\leftrightarrow+2}$	$E_V + 0.482$	--	--
	$Sb_{S(3)}^{\ +2\leftrightarrow+1}$	$E_V + 0.388$	--	--
	$Sb_{S(3)}^{\ 0\leftrightarrow-1}$	$E_V + 0.409$	--	--

	$S_{Sb(2)}{}^{0\leftrightarrow-1}$	$E_V + 0.490$	--	--
	$V_{S(1)}{}^{-2\leftrightarrow-1}$	$E_V + 0.621$	--	--
	$V_{S(1)}{}^{+1\leftrightarrow0}$	$E_V + 0.597$	--	--
	$V_{S(3)}{}^{+1\leftrightarrow0}$	$E_V + 0.594$	Hole	3.77×10^{-16}
	$Sb_{S(1)}{}^{0\leftrightarrow-1}$	$E_V + 0.564$	--	--
	$Sb_{S(1)}{}^{+2\leftrightarrow+1}$	$E_V + 0.604$	--	--
	$Sb_{S(2)}{}^{0\leftrightarrow-1}$	$E_V + 0.607$	Hole	4.00×10^{-17}
	$Sb_{S(3)}{}^{+3\leftrightarrow+2}$	$E_V + 0.756$	--	--
	$S_{Sb(1)}{}^{+\leftrightarrow0}$	$E_V + 0.697$	Hole	9.91×10^{-18}

Supplementary Table 6. Franck–Condon relaxation energies and internal Stokes shifts from the CC diagrams.

Defect	λ_i (eV)	λ_f (eV)	ΔE_{Stokes} (eV)
$V_{S(3)}$	0.67	0.59	1.26
$Sb_{S(2)}$	0.32	0.36	0.68

Supplementary Table 7. Defect-related radiative emission energies and red-shifts for the hole capture.

Defect	E_t (eV)	λ_f (eV)	$E_{PL}{}^{h}$ (eV)	$E_g - E_{PL}{}^{h}$ (eV)
$V_{S(3)}$	0.59	0.59	~0	1.75
$Sb_{S(2)}$	0.61	0.36	0.25	1.50

Supplementary Table 8. Fitting results for impedance spectra of the control and 4mM-SC Sb_2S_3 solar cells by using the Z-view software, measured under dark at the bias voltage of 0.7 V.

Device	R_s (Ω cm^2)	R_{rec} (KΩ cm^2)	CPE-T (F)	CPE-P (F)
Control	3.09	4.51	5.39×10^{-9}	0.98
4mM-SC	2.19	10.66	8.73×10^{-9}	0.95

Supplementary References

1. Sengupta, S., Aggarwal, R. & Golan, Y. The effect of complexing agents in chemical solution deposition of metal chalcogenide thin films. *Mater. Chem. Front.* **5**, 2035-2050 (2021).
2. Maghraoui-Meherzi, H., Ben Nasr, T., Kamoun, N. & Dachraoui, M. Structural, morphology and optical properties of chemically deposited Sb_2S_3 thin films. *Physica B* **405**, 3101-3105 (2010).
3. Dunlap-Shohl, W. A., Zhou, Y., Padture, N. P. & Mitzi, D. B. Synthetic approaches for halide perovskite thin films. *Chem. Rev.* **119**, 3193-3295 (2019).
4. Jung, M., Ji, S. G., Kim, G. & Seok, S. I. Perovskite precursor solution chemistry: from fundamentals to photovoltaic applications. *Chem. Soc. Rev.* **48**, 2011-2038 (2019).
5. Si, H. *et al.* Deciphering the NH_4PbI_3 intermediate phase for simultaneous improvement on nucleation and crystal growth of perovskite. *Adv. Funct. Mater.* **27**, 1701804 (2017).
6. Diliegros-Godines, C., Cruz, J. S., Mathews, N. R. & Pal, M. Effect of Ag doping on structural, optical and electrical properties of antimony sulfide thin films. *J. Mater. Sci.* **53**, 11562-11573 (2018).
7. Wang, S. *et al.* A novel multi-sulfur source collaborative chemical bath deposition technology enables 8%-efficiency Sb_2S_3 planar solar cells. *Adv. Mater.* **34**, 2206242 (2022).
8. Zhang, L. *et al.* Mechanistic study of the transition from antimony oxide to antimony sulfide in the hydrothermal process to obtain highly efficient solar cells. *ChemSusChem* **16**, 202202049 (2023).
9. Wang, X. *et al.* Manipulating the electrical properties of $Sb_2(S,Se)_3$ film for high-efficiency solar cell. *Adv. Energy Mater.* **10**, 2002341 (2020).
10. Agawane, G. L. *et al.* Non-toxic complexing agent Tri-sodium citrate's effect on chemical bath deposited ZnS thin films and its growth mechanism. *J. Alloy Compd.* **535**, 53-61 (2012).
11. Wang, X. W., Li, Z. Z., Kavanagh, S. R., Ganose, A. M., & Walsh, A. Lone pair driven anisotropy in antimony chalcogenide semiconductors. *Phys. Chem. Chem. Phys.* **24**, 7195-7202 (2022).
12. Lang, D. V. Deep-level transient spectroscopy: a new method to characterize traps in semiconductors. *J. Appl. Phys.* **45**, 3023–3032 (1974).
13. Jung, J., Bae, D., Kim, S. & Kim, H-D. Self-rectifying resistive switching phenomena observed in Ti/ZrN/Pt/p-Si structures for crossbar array memory applications. *Appl. Phys. Lett.* **118**, 112106 (2021).
14. Kim, S., Márquez, J. A., Unold, T. & Walsh, A. Upper limit to the photovoltaic efficiency of imperfect crystals from first principles. *Energ. Environ. Sci.*,2020,**13**, 1481-1491 (2020).
15. Qi, Y., Li, Y. & Lin, Q. Engineering the charge extraction and trap states of Sb_2S_3 solar cells. *Appl. Phys. Lett.* **120**, 221102 (2022).
16. Liu, X. N. *et al.* Grain Engineering of Sb_2S_3 thin films to enable efficient planar solar cells with high open-circuit voltage. *Adv. Mater.* **36**, 2305841 (2024).
17. Itzhaik, Y., Niitsoo, O., Page, M., & Hodes, G. Sb_2S_3-sensitized nanoporous TiO_2 solar cells. *Phys. Chem. C* **113**, 4254-4256 (2009).
18. Chang, J. A., *et al.* High-performance nanostructured inorganic-organic heterojunction solar cells. *Nano Lett.* **10**, 2609-2612 (2010).
19. Im, S. H. *et al.* Toward interaction of sensitizer and functional moieties in hole-transporting materials for efficient semiconductor-sensitized solar cells. *Nano Lett.* **11**, 4789-4793 (2011).

20. Chang, J. A. *et al.* Panchromatic photon-harvesting by hole-conducting materials in inorganic-organic heterojunction sensitized-solar cell through the formation of nanostructured electron channels. *Nano Lett.* **12**, 1863-1867 (2012).
21. Boix, P. P. *et al.* From flat to nanostructured photovoltaics: balance between thickness of the absorber and charge screening in sensitized solar cells. *ACS Nano* **6**, 873-880 (2012).
22. Ito, S., Tsujimoto, K., Nguyen, D-C., Manabe, K. & Nishino, H. Doping effects in Sb_2S_3 absorber for full-inorganic printed solar cells with 5.7% conversion efficiency. *Int. J. Hydrogen Energ.* **38**, 16749-16754 (2013).
23. Muto, T., Larramona, G. & Dennler, G. Unexpected performances of flat Sb_2S_3-based hybrid extremely thin absorber solar cells. *Appl. Phys. Express* **6**, 072301 (2013).
24. Choi, Y. C., Lee, D. U., Noh, J. H., Kim, E. K. & Seok, S. I. Highly improved Sb_2S_3 sensitized-inorganic-organic heterojunction solar cells and quantification of traps by deep-level transient spectroscopy. *Adv. Funct. Mater.* **24**, 3587-3592 (2014).
25. Kim, D. H. *et al.* Highly reproducible planar Sb_2S_3-sensitized solar cells based on atomic layer deposition. *Nanoscale* **6**, 14549-14554 (2014).
26. You, M. S. *et al*. Oxide-free Sb_2S_3 sensitized solar cells fabricated by spin and heat-treatment of Sb(III)(thioacetamide)$_2$Cl$_3$. *Org. Electron.* **21**, 155-159 (2015).
27. Zimmermann, E. *et al.* Toward high-efficiency solution-processed planar heterojunction Sb_2S_3 solar cells. *Adv. Sci.* **2**, 1500059 (2015).
28. Raptis, D *et al*. Investigation of efficient protocols for the construction of solution-processed antimony sulphide solid-state solar cells. *RSC Adv.* **6**, 49537-49542 (2016).
29. Yuan, S. *et al.* Efficient planar antimony sulfide thin film photovoltaics with large grain and preferential growth. *Sol. Energ. Mater. Sol. C.* **157**, 887-893 (2016).
30. Yuan, S. *et al.* Postsurface selenization for high performance Sb_2S_3 planar thin film solar cells. *ACS Photonics* **4**, 2862-2870 (2017).
31. Tang, R. *et al.* Vacuum assisted solution processing for highly efficient Sb_2S_3 solar cells. *J. Mater. Chem. A* **6**, 16322-16327 (2018).
32. Zhang, Y. *et al.* Phosphotungstic acid regulated chemical bath deposition of Sb_2S_3 for high-efficiency planar heterojunction solar cell. *Energy Technol.* **6**, 2126-2131 (2018).
33. Yin, Y. *et al.* Composition engineering of Sb_2S_3 film enabling high performance solar cells. *Sci. Bull.* **64**, 136-141 (2019).
34. Jiang, C. *et al.* Alkali metals doping for high-performance planar heterojunction Sb_2S_3 solar cells. *Sol. RRL* **3**, 1800272 (2019).
35. Nie, R. & Seok, S. I. Efficient antimony-based solar cells by enhanced charge transfer. *Small Methods* **4**, 1900698 (2019).
36. Jin, X. *et al.* In situ growth of [hk1]-oriented Sb_2S_3 for solution-processed planar heterojunction solar cell with 6.4% efficiency. *Adv. Funct. Mater.* **30**, 2002887 (2020).
37. Ying, C., Guo, F., Cui, X., Sun, X. & Shi, C. Influence of N-butyldithiocarbamic acid content on the properties of Sb_2S_3 sensitized TiO_2 nanorod arrays and photovoltaic performance of the corresponding solar cells. *Chem. Select* **6**, 6507-6511 (2021).
38. Ning, H. *et al.* Enhancing the efficiency of Sb_2S_3 solar cells using dual-functional potassium doping. *Sol. Energ. Mater. Sol. C.* **221**, 110816 (2021).
39. Peng, X. Q. *et al.* Negative-pressure sulfurization of antimony sulfide thin films for generating a record open-circuit voltage of 805 mV in solar cell applications. *J. Mater. Chem. A* **11**, 19298-

19307 (2023).
40. Shen, G. H. *et al.* Strong chelating additive and modified electron transport layer for 8.26%-Efficient Sb_2S_3 solar cells. *Adv. Energy Mater.* **15**, 2406051 (2025).
41. Yu, F. C. *et al.* In-situ sulfur passivation enabling high open-circuit voltage in hydrothermally Sb_2S_3 solar cells. *Mater. Today Energy* **54**, 102083 (2025).
42. Yang, Z. L. *et al.* Ultrafast self-trapping of photoexcited carriers sets the upper limit on antimony trisulfide photovoltaic devices. *Nat. Commun.* **10**, 4540 (2019).
43. Jia, Z. L. *et al.* Charge-carrier dynamics of solution-processed antimony- and bismuth-based chalcogenide thin films. *ACS Energy Lett.* **8**, 1485-1492 (2023).
44. Zeng, Y. Y. *et al.* Quasi-vertically-orientated antimony sulfide inorganic thin-film solar cells achieved by vapor transport deposition. *ACS Appl. Mater. Interfaces* **12**, 22825-22834 (2020).
45. Mao, Y. *et al.* Molten salts assisted interfacial engineering for efficient and low-cost full-inorganic antimony sulfide solar cells. *Adv. Funct. Mater.* **32**, 2208409 (2022).
46. Krautmann, R. *et al.* Low processing temperatures explored in Sb_2S_3 solar cells by close-spaced sublimation and analysis of bulk and interface related defects. *Sol. Energ. Mater. Sol. C.* **251**, 112139 (2023).
47. Lin, L. M. *et al.* Ambient confined-space annealing for crystallization enhancement and defect passivation in Sb_2S_3 thin-film solar cells. *Nano-Micro Lett.* **18**, 359 (2026).
48. Tao, Y. F. *et al.* Cs doping engineering effectively improves photovoltaic performance of Sb_2S_3 solar cells. *J. Phys. Chem. C.* **129**, 17946-17954 (2025).
49. Yu, Z. *et al.* Copper-based semiconductor nanocrystal hole-transport layers enable efficient and stable Sb_2S_3 solar cells. *ACS Appl. Mater. Interfaces* **17**, 42118-42127 (2025).
50. Tao, Y. *et al.* Cd source optimization enhances solution-processed Sb_2S_3 solar cell efficiency. J. Alloy. Compd. **1039**, 182943 (2025).
51. Sharma, I. *et al.* Vapor-transport-deposited Sb_2S_3 thin-film solar cells: Tailoring photovoltaic properties through deposition temperature. *J. Power Source. Adv.* **26**, 100143 (2024).
52. Li, Y. *et al.* Precursor engineering of solution-processed Sb_2S_3 solar cells. *Small* **20**, 2308895 (2024).
53. Wang, Z. *et al.* Self-assembled growth of quasi-vertically oriented Sb_2S_3 thin films for substrate structured solar cells. *Ceram. Int.* **50**, 4729-4736 (2024).
54. Chihi, A. Gamma-irradiated stibnite thin films set a remarkable benchmark performance for photoelectrochemical water splitting. *RSC Adv.* **14**, 12475-12495 (2024).
55. Yang, M. J. *et al.* Tailoring the crystallographic orientation of a Sb_2S_3 thin film for efficient photoelectrochemical water reduction. *ACS Catal.* **12**, 8175-8184 (2022).
56. Wang, L. *et al.* A transparent, high-performance, and stable Sb_2S_3 photoanode enabled by heterojunction engineering with conjugated polycarbazole frameworks for unbiased photoelectrochemical overall water splitting devices. *Adv. Mater.* **34**, 2200723 (2022).
57. Han, J. *et al.* Multidentate anchoring through additive engineering for highly efficient Sb_2S_3 planar thin film solar cells. *J. Mater. Sci. Technol.* **89**, 36-44 (2021).
58. Ilic, N., Zatsepin, D. A., Biryukov, D. Y., Zatsepin, A. F., Validzic, I. Temperature-dependent photoluminescence of the synthesized Sb_2S_3 particles: From nanoparticles to crystalline samples. *Opt. Mater.* **162**, 116803 (2025).
59. Liu, X. H. *et al.* Improving the optoelectronic properties of single-crystalline antimony sulfide rods through simultaneous defect suppression and surface cleaning. *J. Mater. Chem. A* **11**, 8826-

8835 (2023).
60. Park, Y. S. *et al.* Surface-passivated vertically oriented Sb_2S_3 nanorods photoanode enabling efficient unbiased solar fuel production. *Adv. Energy Mater.* **13**, 2301166 (2023).
