# Supplementary material for: Antimony sulfide photovoltaics with high open-circuit voltage not limited by self-trapped excitons
Source: Nat Commun. 2026 Aug 19;17:9914. doi: 10.1038/s41467-026-76845-1 (PMC13583045; doi:10.1038/s41467-026-76845-1)
Supplement: Supplementary file 2 [file 41467_2026_76845_MOESM2_ESM.pdf]

nature portfolio

Corresponding author(s): Aron Walsh, Robert L. Z. Hoye, Ru Zhou

Solar Cells Reporting Summary

Nature Portfolio wishes to improve the reproducibility of the work that we publish. This form is intended for publication with all accepted papers reporting the characterization of photovoltaic devices and provides structure for consistency and transparency in reporting. Some list items might not apply to an individual manuscript, but all fields must be completed for clarity.

For further information on Nature Research policies, including our data availability policy, see Authors & Referees.

Experimental design

Please check the following details are reported in the manuscript, and provide a brief description or explanation where applicable.

1. Dimensions

Item	Yes/No	Description
Area of the tested solar cells	☒ Yes ☐ No	0.06 cm2. Described in the Methods section.<br>*Explain why this information is not reported/not relevant.*
Method used to determine the device area	☒ Yes ☐ No	The device area was defined using a 0.06 cm2 mask, stated in the Methods part.<br>*Explain why this information is not reported/not relevant.*

2. Current-voltage characterization

Item	Yes/No	Description
Current density-voltage (J-V) plots in both forward and backward direction	☐ Yes ☒ No	Negligible hysteresis observed for Sb2S3 solar cells. Refer to our previous work (Light: Sci. & Appl. 2024, 13,281).
Voltage scan conditions	☒ Yes ☐ No	Backward direction with a scanning rate of 370 mV s–1 (voltage step of 11.1 mV and delay time of 30 ms).<br>*Explain why this information is not reported/not relevant.*
Test environment	☒ Yes ☐ No	25 oC, air environment, 40% relative humidity.<br>*Explain why this information is not reported/not relevant.*
Protocol for preconditioning of the device before its characterization	☒ Yes ☐ No	No preconditioning before testing.<br>*Explain why this information is not reported/not relevant.*
Stability of the J-V characteristic	☐ Yes ☒ No	*Provide a description of the method used. The stability of the J-V characteristic can be verified with time evolution of the maximum power point or with the photocurrent at maximum power point; see ref. 5 for details.*<br>*Explain why this information is not reported/not relevant.*

3. Hysteresis or any other unusual behaviour

Item	Yes/No	Description
Description of the unusual behaviour observed during the characterization	☐ Yes ☒ No	*Provide a description of hysteresis or any other unusual behaviour observed during the characterization.*<br>*Explain why this information is not reported/not relevant.*
Related experimental data	☐ Yes ☒ No	*Provide a description of the related experimental data.*<br>*Explain why this information is not reported/not relevant.*

4. Efficiency

Item	Yes/No	Description
External quantum efficiency (EQE) or incident photons to current efficiency (IPCE)	☒ Yes ☐ No	QTest Station 1000 ADI system under the DC mode, with details specified in the Methods part.<br>*Explain why this information is not reported/not relevant.*
A comparison between the integrated response under the standard reference spectrum and the response measure under the simulator	☒ Yes ☐ No	Fig. 2d.<br>*Explain why this information is not reported/not relevant.*

For tandem solar cells, the bias illumination and bias voltage used for each subcell

☐ Yes ☒ No

Provide a description of the measurement conditions.

Single-junction solar cells reported in this work.

5. Calibration

Light source and reference cell or sensor used for the characterization

☒ Yes ☐ No

Newport Oriel Sol 3A Solar Simulator (AM 1.5, 100 mW cm-2), calibrated with a standard cell (91150V).

Explain why this information is not reported/not relevant.

Confirmation that the reference cell was calibrated and certified

☒ Yes ☐ No

National institute of standards and technology (NIST).

Explain why this information is not reported/not relevant.

Calculation of spectral mismatch between the reference cell and the devices under test

☐ Yes ☒ No

Provide a value of the spectral mismatch and/or a description of how it has been taken into account in the measurements.

No spectral mismatch was performed.

6. Mask/aperture

Size of the mask/aperture used during testing

☒ Yes ☐ No

Shadow mask with an aperture area of 0.06 cm2.

Explain why this information is not reported/not relevant.

Variation of the measured short-circuit current density with the mask/aperture area

☐ Yes ☒ No

Report the difference in the short-circuit current density values measured with the mask and aperture area.

No obvious variation.

7. Performance certification

Identity of the independent certification laboratory that confirmed the photovoltaic performance

☐ Yes ☒ No

Identify the independent certification laboratory.

Explain why this information is not reported/not relevant.

A copy of any certificate(s)

☐ Yes ☒ No

Certificate copies should be provided in the Supplementary information. Please state the supplementary item number.

Explain why this information is not reported/not relevant.

8. Statistics

Number of solar cells tested

☒ Yes ☐ No

12 cells tested for each condition.

Explain why this information is not reported/not relevant.

Statistical analysis of the device performance

☒ Yes ☐ No

Figure 2b.

Explain why this information is not reported/not relevant.

9. Long-term stability analysis

Type of analysis, bias conditions and environmental conditions

☒ Yes ☐ No

Supplementary Fig. 2. Unencapsulated devices measured after 30 days of storage in the dark under ambient temperature in a cabinet with 15% relative humidity.

Explain why this information is not reported/not relevant.
