# Supplementary material for: Antimony sulfide photovoltaics with high open-circuit voltage not limited by self-trapped excitons
Source: Nat Commun. 2026 Aug 19;17:9914. doi: 10.1038/s41467-026-76845-1 (PMC13583045; doi:10.1038/s41467-026-76845-1)
Supplement: Supplementary file 3 [file 41467_2026_76845_MOESM3_ESM.pdf]

nature portfolio

Peer Review File

Antimony sulfide photovoltaics with high open-circuit voltage not limited by self-trapped excitons

Corresponding Author: Professor Robert Hoye

This file contains all reviewer reports in order by version, followed by all author rebuttals in order by version.

Version 0:

Reviewer comments:

Reviewer #1

(Remarks to the Author)
Summary / Key results
This manuscript addresses the nature of the open-circuit voltage (Voc) limitations in Sb2S3 solar cells insists on the external defect nature rather than Voc limitation due to self-trapping. The authors propose the chelating-agent-assisted CBD growth of Sb2S3, which increases crystallite size reduces grain boundaries, thereby reducing extrinsic defect density suppressing recombination. They report improved Voc interpret DLTS/impedance/light-intensity measurements, as well as ultrafast TAS as evidence for reduced trap-assisted recombination in the treated films. The chosen material is timely due to its earth-abundance, non-toxicity pronounced optical light absorption. The experimental techniques methodologies are up to date; the experimental dataset is generally of good quality. The fabrication procedure is clearly described.
Comments:
1. The research question 'whether it is possible to overcome the Voc limit set by the self-trapping in the paper (Yang et al. 2019)' is rather a comment/response to (Yang et al. 2019) does not seem to be critical for the emerging PV field. Self-trapping does not necessarily put the intrinsic limit on Sb2S3 performance in solar cells, since self-trapping can be mitigated itself (Grad et al. 2021).

2. The validity of the conclusions (trap-assisted Voc loss) based on the presented results is weak.

How does the paper rule out the self-trapping in the material? Could DLTS fail in presenting the self-trapping states, which might be close to the band edges (Liu et al. 2023) ?
Is the combination of DLTS first-principle calculations sufficient to prove the extrinsic defect nature of the Voc limit? What about the CCD coordinate shift upon the charge transition (Figure 5d,e), smooth SCLC curve of the control sample the formation of sulfide radicals (S^(-)) with ns-lifetime as a result of the localisation of photogenerated holes on the S atom within the Sb2S3 lattice – these features might be the signatures of the polarons.
It would be beneficial to include additional evidence that can discriminate between extrinsic defect trapping intrinsic self-trapping, like the temperature-dependent TA/PIA signatures of self-trapped states, temperature dependence of recombination kinetics consistent with polaronic capture, a clear argument why this chelation approach would not change lattice relaxation/self-trapping probabilities.
Will it be useful to investigate Sb2S3 with no midgap states obtained by the incorporation of a few atomic% of isovalent cation into Sb2S3 reported recently (Miliaieva et al. 2025) to check if the polaronic signatures still persist even without midgap defects? mainly, what effect does the absence of Sb2S3 midgap states have on the solar cell performance parameters?

3. The work shows only a modest improvement in Sb2S3 Voc, 1.6% higher than the recently reported Voc of 0.811 V (Yu et al. 2025). Solar cell maximum efficiency of 7.67% is comparable to the reported before. There is no novelty in the fabrication procedure, characterisation methods used, or the problematics. These put the work among incremental works.

4. The absorber thickness varies strongly across samples (e.g., ~506 nm control vs ~318 nm for 4mM-SC; down to ~116 nm for 8mM-SC). This affects optical generation, electric-field distribution, series resistance, any thickness-scaled transport analysis. It would be beneficial for all thickness-sensitive electrical comparisons (e.g. SCLC) to use appropriate normalisation and/or a control sample thickness-matched to the test samples, as demonstrated in literature (e.g., Kotadiya et al., 2019) or by preparing thickness-matched films via growth time adjustment.

With respect appreciation for the work performed,
Daria Miliaieva

References

Grad, Lisa, Fabian O. Von Rohr, Matthias Hengsberger, Jürg Osterwalder. 2021. “Charge Carrier Dynamics Self-Trapping on Sb 2 S 3 (100).” Physical Review Materials 5 (7): 075401. https://doi.org/10.1103/PhysRevMaterials.5.075401.

Liu, Yun, Julia Wiktor, Bartomeu Monserrat. 2023. “Strong Electron-Phonon Coupling Carrier Self-Trapping in Sb$_2$S$_3$.” Physical Review Materials 7 (8): 085401. https://doi.org/10.1103/PhysRevMaterials.7.085401.

Miliaieva, Daria, Vojtech Nadazdy, Mykhailo Koltsov, et al. 2025. “Electronic Structure Defect States in Bismuth Antimony Sulphides Identified by Energy-Resolved Electrochemical Impedance Spectroscopy.” Journal of Physics: Energy 7 (3): 035012. https://doi.org/10.1088/2515-7655/add59f.

Yang, Zhaoliang, Xiaomin Wang, Yuzhong Chen, et al. 2019. “Ultrafast Self-Trapping of Photoexcited Carriers Sets the Upper Limit on Antimony Trisulfide Photovoltaic Devices.” Nature Communications 10 (1): 4540. https://doi.org/10.1038/s41467-019-12445-6.

Yu, Fucheng, Zehao Wu, Cheng Qian, et al. 2025. “In-Situ Sulfur Passivation Enabling High Open-Circuit Voltage in Hydrothermally Sb2S3 Solar Cells.” Materials Today Energy 54 (December): 102083. https://doi.org/10.1016/j.mtener.2025.102083.

Reviewer #2

(Remarks to the Author)
The manuscript reports a record V_{OC} of 824 mV in Sb_2S_3 solar cells, surpassing the ~800 mV limit previously proposed by Yang et al. (Nature Commun. 2019). While the experimental achievement is commendable, the theoretical discussion regarding the origin of this "limit" the distinction between self-trapping (polarons) defect-trapping is logically flawed incomplete.

1. The authors frame their work as "breaking" the 0.8 V limit derived by Yang et al. It is crucial to acknowledge that Yang’s 0.8 V limit was calculated based on the assumption that the observed ~0.6 eV Stokes shift originates from intrinsic self-trapping (polarons), which would represent a fundamental physical limit. By achieving 0.824 V, the authors experimentally demonstrate that the voltage loss is not fundamental but technological (i.e., defect-mediated). This implies that Yang's initial assignment of the Stokes shift to intrinsic polarons was likely incorrect. Therefore, the authors are not "breaking a fundamental barrier" but rather disproving a hypothesis. The manuscript must be rewritten to explicitly discuss the origin of Yang's 0.8 V value state that the current results invalidate the assumption that intrinsic self-trapping is the dominant recombination pathway.

2. The authors perform DFT (HSE) calculations to identify point defects (V_S, Sb_S) assign them to the DLTS signals. However, both defect states self-trapped polaron states can be calculated using hybrid functionals. To conclusively claim that they have overcome self-trapping by reducing defects, the authors must theoretically compare the two. The authors should perform calculations for the self-trapped hole/electron states (polarons) in Sb_2S_3. By comparing the formation energies capture cross-sections of the Polaron state vs. the Defect state, the authors can theoretically demonstrate which mechanism is energetically favorable. Without calculating the polaron state, the conclusion that defects dominate is theoretically incomplete.

3. The authors attribute the energy discrepancy (approx. 0.1-0.2 eV) between the calculated defect levels the experimental DLTS signals (H1/H2) to "temperature effects." This explanation appears speculative is cited without specific computational support for this material system. Given the high precision claimed in the rest of the work, attributing a significant energy shift to thermal effects without calculation is scientifically loose. Furthermore, if the previous "0.8 V limit" was indeed due to defects (misidentified as polarons by Yang), the theory must explain the spectroscopic origin. The authors should discuss whether the calculated relaxation energy (Frank-Condon shift) of their identified defects matches the large ~0.6 eV Stokes shift observed in previous literature, which would provide a solid link between their defect theory the historical spectral data.

Reviewer #3

(Remarks to the Author)
In “Breaking the 800 mV open-circuit voltage barrier in antimony sulfide photovoltaics”, a new record Voc is reported for Sb2S3, following optimization of the chemical bath deposition process by addition of a complexing agent.

Breaking the 800 mV Voc barrier is an important milestone. The paper is full of interesting data from (advanced) characterization (advanced) first-principles calculations. It will be very valuable for the Sb2(S,Se)3 community.

May main criticism is related to some overstatements to some potentially erroneous conclusions.

• The authors repeatedly emphasize that the demonstration of a 824 mV Voc is a sign that Sb2S3 cells are not intrinsically limited by self-trapped excitons. This is based on a Voc limit of 0.8 V for a device limited by self-trapped excitons. Going back to the original source, this 0.8 V limit was just a rough estimate rather than an exact value (it is also given with just one significant digit, making it equal to the new record Voc). While a new record Voc is a great achievement it shows that progress on Sb2S3 has not stalled, it does not prove that Sb2S3 are not limited by self-trapped excitons. These statements are misleading should be removed.
• Page 10. "In polycrystalline films, recombination losses are largely caused by GBs etc.". This is a very hand-waving argument that is often not true. There are many published examples of CIGS solar cells where smaller grains gave more efficient devices because the dominant recombination mechanism is not at the grain boundaries. I cannot find any evidence in this paper to link the improved performance to grain boundaries, even from the kelvin probe measurements. On the contrary, the grain boundaries are already large in the control film (much larger than the film thickness) transport is in the out-of-plane direction. Yes, there is a correlation between GB density efficiency as stated on page 22, line 546, but there does seem to be a cause-effect relationship.
• It is stated that film thickness changes quite a lot between the control sample (500 nm) the samples where SC was used (down to 116 nm). The PV efficiency differences between these samples are not massive (Fig. 2). So, there is a possibility that the efficiency changes are heavily influenced by the different thicknesses. The authors should present convincing evidence (such as a thickness study on the control sample or on the 4mM-SC sample) showing that the variable film thickness is not the main contributor to the efficiency trends.
• Page 12. "The reduced fluctuation of the current intensity for the 4mM-SC Sb2S3 film should be conducive to the local photocurrent generation collection, which might effectively suppress charge-carrier recombination at GBs". I just don't understand this statement. Please clarify.
• Page 20. The authors give a calculated HSE band gap of 1.79 eV a maximum Voc of 1.70 V in the radiative limit. This Voc seems to violate physics (higher than expected from Shockley-Queisser limit). Please clarify.
• C-V measurements are presented values of built in voltage are given. However, the films are relatively thin low carrier concentrations are expected from the UPS data in the SI (and for Sb2S3 in general). For 300 nm film thickness, the carrier concentration would need to be above E16 cm-3 for the analysis to be valid (avoiding geometric capacitance effects charge injection). Reference: Figure 1c in 10.1126/science.abd8014. It seems likely that the present Sb2S3 films have lower carrier concentration. In that case, the analysis would not be valid. Please clarify.

Minor comments:

• Page 6. "SC was used as a complexing additive etc.". Already at this point, I suggest making a reference to the section in the SI where an (interesting) interpretation of the CBD deposition mechanism in the presence of SC is given.
• The whole preliminary description of DLTS (page 14 15) is useful but very long it's more like textbook information. I suggest moving it to the SI

Reviewer #4

(Remarks to the Author)

This research article by Zhou et al. reports a study of modified chemical bath deposition (CBD) of Sb2S3, resulting in solar cells with an open-circuit voltage (Voc) exceeding 800 mV. The authors introduce the sodium citrate (SC) complex agent to regulate the CBD of Sb2S3, thereby reducing grain boundary (GB) density suppressing mid-gap defect states. As a result, they achieved Sb2S3 solar cells with a record high Voc of 824 mV. The authors further investigate defects by combining deep-level transient spectroscopy (DLTS) measurements with density-functional theory (DFT) defect calculations, providing a comprehensive understanding of defect properties in Sb2S3. Overall, this is a well-organized study with relatively high significance novelty. I would consider recommending this manuscript for publication if the remaining issues are addressed. Detailed comments are provided below.

[1] In the introduction, the authors claim, 'At present, the VOC of Sb2S3 solar cells has stagnated in the range of 550-770 mV[SZ1] for decades.' However, in Fig. 2e the corresponding Supplementary table, at least two papers report Voc values of ~800 815 mV. The authors should correct this statement to be accurate about the establishment in the field.

[2] Could the authors provide references to support the statement, 'This mechanism is linked to their experimental observation of a 600 meV red-shift in photoluminescence (PL) compared to the optical bandgap, along with polarized light emission from Sb2S3 single crystals?' The PL redshift could also be related to the emission from defect/impurity levels. Why is it claimed as evidence for self-trapped exciton states, which typically are not as deep as 600 meV?

[3] In the statement, 'Fig. 3a b shows top-view scanning electron microscopy (SEM) images of Sb2S3 thin films prepared without the SC additive (i.e., the control sample) with different concentrations of 4 mM SC,' the words "with different

concentrations of 4 mM SC" are typos since the concentration is at 4 mM.

[4] There are typos in the figure numbers.

In 'Fig. 3a gives the dependence of GB density on the SC concentration for different Sb_2S_3 films,' it should be Fig. 3c rather than 3a.

In 'Fig. 3c shows the high-resolution TEM (HRTEM) images of three arbitrarily selected points A1, A2, A3, as labeled in Fig. 3b.' The correct figure numbers should be Fig. 3e Fig. 3d.

[5] In the authors' hypothesis of SC's working mechanism, additional evidence is needed to determine whether PH affects grain size GB density. Though PH does not significantly affect device performance, it may change the nucleation growth. If this is true, then the passivation effect of SC complexation to S vacancies Sb antisites could be the driving force for the enhanced Voc.

[6] Parameters variables in Eq. 2 need to be further explained.

[7] For Eqs. 3 4, could emission time be different for electrons holes?

[8] Since C-V measurements were performed, the depletion width apparent carrier density should be estimated discussed.

Version 1:

Reviewer comments:

Reviewer #1

(Remarks to the Author)
I thank for the additional work on the manuscript. After presenting the temperature-dependent mobility data, the mesurements on thickness-comparable samples, authors' clarification on the theory the novelty importance of the data, the manuscript is ready for publishing as it is with no further comments from my side. All the best!

Reviewer #2

(Remarks to the Author)
The Reviewer's main concern is still only partially resolved. The manuscript does not quantitatively connect the proposed defect-based picture to the previously reported ~0.6 eV Stokes-shifted emission that motivated the self-trapping hypothesis. The response only states that the defect relaxations are "qualitatively consistent," but no explicit calculated relaxation energies or direct comparison to the ~0.6 eV shift are provided. This remains insufficient.

I also do not find the argument based on temperature-induced band-edge shifts convincing in its present form. The cited ~0.2 eV value from Liu et al. (2023) refers to the temperature dependence of the bulk Sb_2S_3 bandgap, not to the temperature dependence of defect transition levels. The manuscript effectively extrapolates from a bulk band-edge shift to a defect-level shift of similar magnitude without demonstrating that this connection holds for the specific defects under discussion. These are not generally equivalent quantities. The effect of electron-phonon coupling on the band edges is not the same as its effect on deep defect levels, so a similar numerical scale does not by itself establish a physical explanation for the 0.1-0.2 eV discrepancy.

More importantly, this temperature-effect argument does not actually resolve Reviewer's central spectroscopic point. Reviewer asked whether the calculated defect relaxation energies (Franck-Condon shifts) can explain the previously reported ~0.6 eV Stokes shift. Instead, the response discusses possible temperature-induced shifts in defect level positions. These are different physical quantities. The former concerns relaxation energy associated with optical emission, whereas the latter concerns the absolute energetic position of defect levels. As written, the manuscript substitutes an argument about level shifts for a question about relaxation energies, therefore does not directly address the key issue.

Reviewer #3

(Remarks to the Author)
The authors have provided reasonable evidence of large polarons rather than small polarons in their Sb_2S_3. They have also reconsidered claims that were not fully backed by experimental evidence, making the paper more convincing without loss of impact. Most of their responses changes are well reasoned appreciated.

There are four aspects that are still not convincing:

1. The title is "Exceeding the 800 mV open-circuit voltage threshold in antimony sulfide photovoltaics ", but it appears that there is a published report of 811 mV Voc for Sb2S3 solar cells (I was not aware of it in the first review round). Thus, the title is not quite truthful should be changed, so that it is not implied that this is the first study to surpass the 800 mV threshold, which is not true. Still, this study reports the highest Voc to date, so it should not be a problem to highlight the achievement.

2. The relationship between efficiency grain boundary density is not convincing. There is no evidence of causation, only correlation. The authors argue that "the overall reduction in trap state density that we measured is correlated with the reduction in GB density [correct]. It is reasonable to deduce that the recombination losses in Sb2S3 are closely associated with GBs [incorrect], where dangling or wrong bonds proliferate, including the GBs located at the film interface film interior [The film surface is a giant grain boundary even for a single-crystalline film, so at the surface it doesn't matter what the grain size is. In fact, surface passivation is essential even in single-crystalline PV technology such as c-Si or III-V. At the film interior it is highly questionable whether GBs matter with grains of this size. Yes, they could, but there is no evidence to support this.]. The authors also state "the Sb2S3-based devices would also inevitably [incorrect] involve the negative effects of GBs with dangling bonds at the surface interior of polycrystalline films, which would be detrimental to device performance [not necessarily correct]." Finally, the authors state "from the c-AFM measurements, it can be seen that the GBs generally have lower conductivity than the grain interiors, suggesting inefficient charge-carrier separation [why would lower conductivity in the dark imply inefficient carrier separation? I do not see the connection] transport [possibly, but only if the conductivity decrease is due to a mobility decrease. If it's due to a decrease in dark carrier concentration, carrier transport under illumination is more likely to be unaffected] as a result of defects at the GBs39. None of these statements makes a convincing case. Insisting on this connection really has no impact on the overall quality of the paper. What if recombination at GBs is not the reason for improved performance, but instead an improved growth process results in lower bulk defect density, which in turn causes the grains to grow bigger? This hypothesis is entirely plausible, it would imply that there is no cause-effect relationship between GB density Voc – only a correlation.

Thus, I think the revised text "It is generally accepted that, in polycrystalline films, dangling or wrong bonds proliferate at GBs, which would result in increased non-radiative recombination losses be detrimental for device performance. Hence, increased device performance with polycrystalline absorbers would often be correlated with reduced GB density" is still too speculative should be removed.

3. Some of the revised text reads. "The reduced surface roughness of the absorber films would favor the formation of an improved heterojunction between Sb2S3 solution-processed HTL on top". Why should this be the case? This seems to be speculative, there are no references to support this claim. Si solar cells are intentionally roughened to enhance light scattering, this can still give > 27% efficient cells.

4. About the 1.70 V open-circuit voltage limit. I understand that using a non-stepwise absorption profile can lead to a different, possibly higher Voc limit than the SQ prediction. However: (i) Figure 1(a) makes it quite confusing for the reader to see the Voc of many PV materials lined up against their band gap their SQ Voc limit, to later be told that in reality the real radiative Voc limit is much higher than what Figure 1(a) says. (ii) Radiative Voc limits that are based on other formalisms than the SQ one are not as established, so they require a higher standard of proof. I do not think it's sufficient for the authors to claim that they used package XYZ obtained a Voc limit of XYZ. The authors should explicitly show the equations assumptions used to derive this limit.

Reviewer #4

(Remarks to the Author)
The authors have adequately addressed the previous round of comments from reviewers. The newly added temperature-dependent mobility measurements support their conclusions.

The reviewer agrees that this work is novel in its experimental demonstration that the performance of Sb2S3 photovoltaics is limited by defects rather than by self-trapping, disproving a previous hypothesis published in the same journal. The study is sound supported by experimental evidence theory.

Therefore, the reviewer recommends this study for publication as is.

Version 2:

Reviewer comments:

Reviewer #2

(Remarks to the Author)
I am satisfied that the temperature-related concern has been adequately addressed.

My principal concern, however, remains unresolved, because the authors' response addresses a different question from the

one I raised. I asked for a quantity intrinsic to the authors' own model: the calculated Franck–Condon relaxation energies, the predicted Stokes shifts, of the proposed dominant defects V_S(3) Sb_S(2). The authors instead argue that the previously reported ~0.6 eV red-shift is atypical in the literature may be an artefact.

These are logically independent. Even if Yang et al.'s value were entirely artefactual, the Stokes shift predicted by the authors' own defect model remains a well-defined quantity that has not been reported, its evaluation does not depend on that measurement at all. An argument about non-universality therefore cannot substitute for the calculation, the reported transition levels capture cross sections are not the relaxation energy in question.

The authors need not explain the ~0.6 eV shift, but they must close the loop on their own mechanism: for at least V_S(3) Sb_S(2), they should report the configuration-coordinate diagrams calculated relaxation energies—which, if the capture cross sections were obtained from non-radiative multiphonon theory, are already available—and state the Stokes shift their model predicts, comparing it with both the ~0.6 eV value the ~0.1 eV shift measured here. A quantitative prediction, whatever its magnitude, would resolve this; reaffirming that the ~0.6 eV shift is atypical would not. This need to be addressed.

Reviewer #3

(Remarks to the Author)
The authors have convincingly addressed my remaining concerns. I recommend the manuscript for publication.

Version 3:

Reviewer comments:

Reviewer #2

(Remarks to the Author)
The authors have addressed all my concerns, I support the publication of this paper.

Open Access This Peer Review File is licensed under a Creative Commons Attribution 4.0 International License, which permits use, sharing, adaptation, distribution and reproduction in any medium or format, as long as you give appropriate credit to the original author(s) and the source, provide a link to the Creative Commons license, and indicate if changes were made.
In cases where reviewers are anonymous, credit should be given to 'Anonymous Referee' and the source.
The images or other third party material in this Peer Review File are included in the article's Creative Commons license, unless indicated otherwise in a credit line to the material. If material is not included in the article's Creative Commons license and your intended use is not permitted by statutory regulation or exceeds the permitted use, you will need to obtain permission directly from the copyright holder.
To view a copy of this license, visit https://creativecommons.org/licenses/by/4.0/

Reviewer comments copied out *ad verbatim* in black, and our responses are in blue. All changes to the main text and supplementary information are in blue.

Reviewer 1:

This manuscript addresses the nature of the open-circuit voltage (V_{OC}) limitations in Sb_2S_3 solar cells and insists on the external defect nature rather than V_{OC} limitation due to self-trapping. The authors propose the chelating-agent-assisted CBD growth of Sb_2S_3, which increases crystallite size and reduces grain boundaries, thereby reducing extrinsic defect density and suppressing recombination. They report improved V_{OC} and interpret DLTS/impedance/light-intensity measurements, as well as ultrafast TAS as evidence for reduced trap-assisted recombination in the treated films. The chosen material is timely due to its earth-abundance, non-toxicity and pronounced optical light absorption. The experimental techniques and methodologies are up to date; the experimental dataset is generally of good quality. The fabrication procedure is clearly described.

Response: We thank the reviewer for her time in going over our paper, and for recognizing the timeliness and importance of this work, as well as the good quality. We have addressed all comments raised, which we believe have strengthened our paper, as detailed below.

Comments:

1. The research question 'whether it is possible to overcome the V_{OC} limit set by the self-trapping in the paper (Yang et al. 2019)' is rather a comment/response to (Yang et al. 2019) and does not seem to be critical for the emerging PV field. Self-trapping does not necessarily put the intrinsic limit on Sb_2S_3 performance in solar cells, since self-trapping can be mitigated itself (Grad et al. 2021).

Response: We would like to thank the reviewer for raising this point, and read with interest the work from Grad *et al.* (*Phys. Rev. Mater.* 2021, 5, 075401). We would like to clarify that self-trapping is indeed an intrinsic process, in the sense that it takes place whether or not a material is defect-free. Self-trapping does indeed place an intrinsic limit on the achievable performance of a material, and has been found to be a severe limitation across many classes of pnictogen-based semiconductors (see for example *Nat. Commun.* 2025, 16, 65; *Appl. Phys. Lett.* 2021, 119, 220501; *ACS Energy Lett.* 2021, 6, 1729-1739; *Adv. Mater.* 2021, 33, 2007057;

Sci. Adv. 2021, 7, eabd3160). Yang *et al.* proposed that self-trapping also takes place in Sb_2S_3, which, if true, would prevent this material from reaching its radiative limits in photovoltaic performance (*Nat. Commun.* 2019, 10, 4540). We would like to elaborate on each of these points (self-trapping being intrinsic, and self-trapping inherently limiting solar absorber performance) separately below.

Self-trapping taking place inherently within a material, rather than depending on whether or not defects are present, is precisely what Grad *et al.* wrote about. Their recommendations on how self-trapping may be avoided in Sb_2S_3 revolved around adjusting the elastic constant. This observation may be generalized by saying that small polaron formation takes place due to strong electron-phonon coupling, either with longitudinal optical (LO) phonons (*i.e.*, Fröhlich coupling) or acoustic phonons. The acoustic coupling constant (g_{ac}) is given by Equation R1:

$$g_{\mathrm{ac}} = \frac{E_{\mathrm{d}}^2}{Ca_0}\frac{m_{\mathrm{eff}}}{3\pi\hbar^2} \tag{R1}$$

In Equation R1, C is the elastic constant, and so increasing the elastic constant would indeed lower g_{ac} and therefore reduce the likelihood of forming small polarons. But more importantly, the deformation potential (E_d) needs to be low, as should the effective mass (m_{eff}). As discussed in the paper from Grad *et al.*, the electronic dimensionality should be high, since an electronic dimensionality of 1D or lower leads to self-trapping being inevitably thermodynamically favoured, regardless of how low g_{ac} is.

The Fröhlich coupling constant (α_{OP}) is given by Equation R2.

$$\alpha_{\mathrm{OP}} = \frac{1}{2}\frac{1}{4\pi\epsilon_0}\left(\frac{1}{\epsilon_{\mathrm{optical}}} - \frac{1}{\epsilon_{\mathrm{static}}}\right)\frac{q^2}{\hbar}\sqrt{\frac{2m_{\mathrm{eff}}}{\hbar\omega_{\mathrm{LO}}}} \tag{R2}$$

In Equation R2, ϵ_0 is the permittivity of free space, $\epsilon_{\mathrm{optical}}$ and $\epsilon_{\mathrm{static}}$ are the optical and static contributions to the dielectric constant, respectively, q is the elementary charge and ω_{LO} the frequency of the dominant LO phonon mode. It can be seen that lowering α_{OP} requires a smaller ionic contribution to the dielectric constant, such that $\epsilon_{\mathrm{optical}}$ and $\epsilon_{\mathrm{static}}$ are closer together, having higher band dispersion, and increasing ω_{LO}. A small ionic contribution to the dielectric constant can be obtained by having elements with a lower Born effective charge and/or having a small bandgap (which increases $\epsilon_{\mathrm{optical}}$). A larger ω_{LO} can be obtained by having lighter elements or stiffer bonds.

Therefore, it can be seen that lowering the acoustic or Fröhlich coupling constant requires fundamental changes to the material, for example, through changes in composition and structure; they cannot be achieved simply through doping, lowering defect density, or changing how the material is grown.

If self-trapping takes place, a material is inherently prevented from reaching the radiative limit in performance. This is because when the material is thermodynamically favoured to relax into a self-trapped state, there is a reduction in the energy separation between the relaxed excited state and ground state, thus reducing the quasi-Fermi level splitting to below what would be expected from the radiative limit. This is what Yang *et al.* discussed in detail (*Nat. Commun.* 2019, 10, 4540), where they observed the photoluminescence peak of Sb_2S_3 to be 0.6 eV below the optical gap, implying that the V_{OC} would be 0.6 V below the radiative limit (1.4 V for a 1.7 eV bandgap). A V_{OC} of 0.8 V is indeed the value that the field has asymptotically approached with Sb_2S_3 solar cells, as we detail in our paper. Self-trapping also causes a severe reduction in charge-carrier mobility because the charge-carrier wavefunction is now restricted to within a unit cell. Thus, rather than band-like transport with mobilities capable of reaching values expected from the effective mass, mobilities are much lower and carrier transport takes place through hopping. This severely restricts diffusion lengths, limiting the achievable external quantum efficiency and fill factor of a photovoltaic device.

It is because of these significant consequences of self-trapping that the hypothesis put forward by Yang *et al.* was so concerning to the field. If this hypothesis were true, then Sb_2S_3 photovoltaics will not be able to progress towards their radiative limit, inhibiting the future progress that could be expected from this material. That is, even making the perfect, defect-free Sb_2S_3 solar cell would not be able to surpass 800 mV V_{OC}; a material with a different composition and structure would have to be used to lower electron-phonon coupling.

Our work disproves this hypothesis by showing that large polarons in fact form in Sb_2S_3. We obtained new temperature-dependent mobility measurements to provide direct experimental proof of this, as detailed in our answer to the next question below. We prove that it is possible to surpass the 800 mV V_{OC} threshold that would have been in place if the hypothesis by Yang *et al.* were correct. The critical implication of our findings is that it is possible to improve Sb_2S_3 toward its fundamental limit in performance. This is important not only for the community working on Sb_2S_3, but also to the wider community working on lead-free perovskite-inspired

materials, where self-trapping has been widely found to limit the photovoltaic performance of these materials.

2. The validity of the conclusions (trap-assisted *V*oc loss) based on the presented results is weak.

How does the paper rule out the self-trapping in the material? Could DLTS fail in presenting the self-trapping states, which might be close to the band edges (Liu et al. 2023)?

Response: We thank the reviewer for raising these important points.

For the first point on ruling out self-trapping, we have collected new data to strengthen our experimental evidence. That is, we measured the temperature-dependence of the charge-carrier mobility in the Sb_2S_3 films (see new Fig. 6a, replicated below). If small polarons formed, charge-carriers would be transported via a hopping mechanism, meaning that the mobility would increase with increasing temperature. By contrast, if large polarons instead formed, the delocalized nature of the wavefunction means that transport is more band-like, and mobility would decrease with increasing temperature. This clear difference in the temperature-dependence of mobility allows us to differentiate between large and small polarons.

As can be seen in Fig. 6a, the mobility decreases with increasing temperature. The dominant charge-carriers in Sb_2S_3 therefore are clearly large polarons, which disproves the hypothesis made in the work from Yang *et al.*

We have provided detailed discussions in the revised manuscript (Page 20, highlighted in blue):

"To verify the formation of delocalized large polarons instead of small polarons in Sb_2S_3, we determined the temperature dependence of the mobility through time of fight (TOF) measurements[62]. It is known that large polarons show a reduction in the overall mobility with increasing temperature because of increased phonon scattering. On the other hand, if small polarons dominate charge-carrier transport, the mobility should increase as the temperature increases because small polaron formation changes the transport mechanism to hopping-based. The device structure for TOF measurements was FTO/Sb_2S_3/Au, and the measurement details can be found in Methods. Note that CBD was unable to grow compact films directly on the FTO substrates, and so we used closed-space sublimation (CSS) to form continuous thin films

that avoid shunting; we would not expect any differences in the trends in mobility with temperature for CBD vs. CSS-grown Sb_2S_3. Fig. 6a shows the obtained mobilities of Sb_2S_3 at different temperatures ranging from 100 to 400 K based on the bilogarithmic plots of transient current vs. transit time (Supplementary Fig. 19). The observation of an obvious decrease in mobility with an increase in temperature is consistent with the behavior of large polarons."

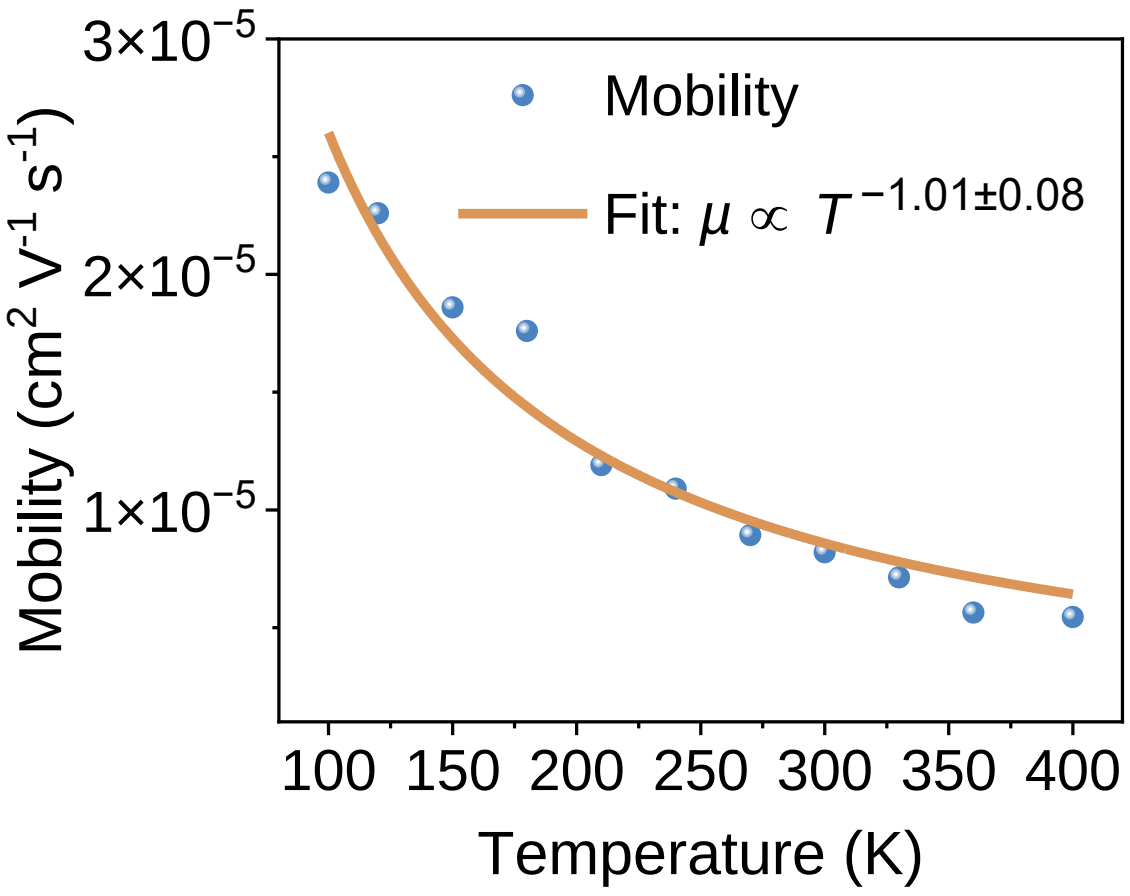

Fig. 6a Temperature-dependent mobility of Sb_2S_3 films determined using time of flight (TOF) measurements, fitted with a power law model that indicates $\mu \propto T^{-1.01\pm0.08}$. Here ±0.08 is the uncertainty of the exponent obtained by fitting the data across all temperatures.

We would like to thank the reviewer for asking about whether we could use DLTS to identify self-trapped states. As the reviewer would be aware, DLTS utilizes the transient capacitance of a p-n junction at varying temperatures as a probe to reflect the changes in the charge state of deep defect centres (greater than 0.1 eV from the band edges). Based on the working principle of DLTS, the detection of transient capacitance involves the trapping and emission of carriers in deep defects (such as vacancies, anti-sites, and interstitials), and the time scale to collect the transient capacitance is typically on the microsecond level (*J. Chem. Phys.* 2020, *153* (12), 124703; *Adv. Funct. Mater.* 2025, e18624). A period width of 100 ms (i.e., a fixed time window fixed by t_1 and t_2) was used in our work, as given in the Supplementary Note 3. In contrast, when there is small polaron formation, decays in the transient capacitance are typically on the picosecond timescale (*Nat. Commun.* 2019, 10, 4540), which we could not resolve by DLTS. Therefore, we believe that DLTS is not capable of probing self-trapped states. The temperature-dependent mobility measurements provide clearer evidence of large polaron formation.

Is the combination of DLTS and first-principle calculations sufficient to prove the extrinsic defect nature of the V_{OC} limit? What about the CCD coordinate shift upon the charge transition (Figure 5d,e), smooth SCLC curve of the control sample and the formation of sulfide radicals (S^(-Ÿ)) with ns-lifetime as a result of the localisation of photogenerated holes on the S atom within the Sb_2S_3 lattice – these features might be the signatures of the polarons.

Response: We thank the reviewer for these valuable comments and helpful suggestions.

(1) We appreciate the reviewer's question regarding the nature of the V_{OC} limit, which relates to the previous question. As explained in detail above, we obtained new temperature-dependent mobility results to directly prove that polarons are large rather than small in Sb_2S_3. This strongly supports our previous computational findings in our previous work (*ACS Energy Lett.* 2022, 7, 2954-2960). In these computations, we introduced an electron or hole to the pristine Sb_2S_3 supercell, distorted the structure, and let it relax to equilibrium with the extra charge. In these calculations, we found that the electron or hole wavefunction remained delocalized, indicating large rather than small polarons. The new temperature-dependent mobility we obtained in our current revised work now provides direct experimental evidence for this, which is valuable for the field.

(2) We thank the reviewer for the suggestion on analyzing the configuration coordinate diagram (CCD) shift. We note that a configuration coordinate shift upon charge transition indicates lattice relaxation associated with a change in charge state. This behavior is not specific to polaron formation, and any defect that captures a carrier will lead to a change in the local bonding environment.

(3) In this work, we used SCLC to estimate trap state densities in Sb_2S_3 solar cells based on the trap-filled limiting voltage (V_{TFL}) values. Both the control and 4 mM-SC samples exhibit typical SCLC curves for electron-only devices, despite the SCLC curve being smoother for the control sample. Similar curve shapes have been widely demonstrated for various material systems, such as organic semiconductors (*Nat. Photonics* 2019, 13, 765-769; *Nat. Mater.* 2018, 17, 329-334; *Front. Electron. Mater.* 2024, 4, 1396521) and inorganic semiconductors (*J. Am. Chem. Soc.* 2019, 141, 17610-17616; *ACS Appl. Energy Mater.* 2024, 7, 1421-1432). We therefore do not believe we could reach conclusions on whether large or small polarons form in Sb_2S_3 from the shape of the SCLC curves, but we would like to thank the reviewer for suggesting this in any case.

(4) As for the origin of sulfide radicals ($S^{-\bullet}$), Christians *et al.* comprehensively investigated hole diffusion in the Sb_2S_3 absorber and hole transfer across the Sb_2S_3/CuSCN interface in the TiO_2/Sb_2S_3/CuSCN system using femtosecond TA spectroscopy, and attributed the photoinduced absorption to the formation $S^{-\bullet}$ species, which arises from hole trapping (*Energy Environ. Sci.* 2014, 7, 1148-1158). They attributed the decay of trapped holes (i.e., the $S^{-\bullet}$ species) to non-radiative electron-hole recombination. We followed this interpretation in our work, where we considered the kinetics of the photoinduced absorption to be associated with non-radiative recombination of electrons by trapped holes. More generally, it is very difficult to disentangle polaronic effects from defect states in TA spectroscopy, and certainly we do not believe it could unambiguously be done with the data we have.

Overall, we believe the new measurements took of the temperature dependence of the mobility of Sb_2S_3 is the best approach to experimentally prove the band-like transport of charge-carriers. We thank the reviewer for their suggestions of alternative approaches to do this.

It would be beneficial to include additional evidence that can discriminate between extrinsic defect trapping and intrinsic self-trapping, like the temperature-dependent TA/PIA signatures of self-trapped states, temperature dependence of recombination kinetics consistent with polaronic capture, and a clear argument why this chelation approach would not change lattice relaxation/self-trapping probabilities.

Response: We thank the reviewer for these comments, and for her continued support with suggestions to help us provide clearer evidence for proving the absence of self-trapping.

(1) This question overlaps with the second question, and our new temperature-dependent mobility measurements are detailed there. If there were strong defect-mediated trapping of charge-carriers, we could also expect to see hopping-based transport. The mobility would then also increase with increasing temperature. That we saw the opposite trend confirms that this did not take place, and that small polarons also did not form.

(2) We thank the reviewer for the suggestion to perform temperature-dependent TA spectroscopy measurements. We considered this carefully, and also thought about performing temperature-dependent optical pump terahertz probe (OPTP) spectroscopy. For the TA

spectroscopy, we explained in our answer to the previous question that it is very difficult to unambiguously disentangle the optical signatures of polarons with $S^{-\bullet}$ species from trapped holes. We do not believe that taking further measurements will help us gain further clarity. For OPTP, this is a powerful technique that measures changes in photoconductivity over time after optical photoexcitation, and can measure with sub-ps time resolution. OPTP on Sb_2S_3 was performed previously at room temperature by Jia *et al* (*ACS Energy Lett.*, **8**, 1485-1492 (2023)). The photoconductivity signal was found to decay over several ps, which is in the ambiguous intermediate range between self-trapping and defect trapping, and indeed Jia *et al.* did not distinguish between these two possibilities. Temperature-dependent mobility provides much clearer evidence that allowed us to unambiguously conclude that large polarons formed. The only way we could improve upon this would be to perform temperature-dependent OPTP measurements, but this is a highly time-intensive measurement that is also very specialized. We would have to arrange time with a collaborator, and it would take months to obtain the measurements with sufficient quality to reach definitive conclusions. Overall, the new temperature-dependent mobility measurements we took are adequate.

To address these points, we have added the following to the discussion section (Pages 24-25, highlighted in blue):

"Although Yang et al. observed PL that was red-shifted to the optical gap, and used this as part of their proposition of self-trapping in Sb_2S_3, other studies reported broad PL emission near the band-edge, with significant temperature-dependence of the PL intensity, which is consistent with defect-dominated emission[65, 75]. Optical pump terahertz probe (OPTP) measurements are also often used to probe whether self-trapping occurs. But previous room-temperature OPTP measurements of solution-processed Sb_2S_3 films were inconclusive on whether the initial fast decay in photoconductivity arose from self-trapping or defect-mediated recombination[65]. In this regard, the temperature-dependent mobility measurements we obtained here provide a clearer indication of large polarons being present. Furthermore, extensive investigations into Sb_2S_3 films have shown high defect densities > 10^{15} cm^{-3} to be common, with strong correlation between the concentration of defects and device performance[6, 67, 76]. These experimental findings support our findings made here, where lowering defect densities in the Sb_2S_3 films was critical to achieve the high V_{OC}s obtained. Moreover, our previous first-principles analyses have also challenged the hypothesis of self-trapping in Sb_2S_3, where delocalized electrons and holes were found to occur in a perfect supercell[31]. Taken together, our new experimental and

computational analyses into defects, coupled with prior polaron calculations, disprove the hypothesis of self-trapping in Sb_2S_3, showing that it is possible to improve the performance of this material towards its radiative limit through defect engineering."

(3) For the last part of the question on why chelation would not affect self-trapping: as detailed in our answer to the first question, self-trapping takes place due to strong electron-phonon coupling. Given that we have established that Sb_2S_3 exhibits band-like transport, achieving self-trapping would then require substantial changes to the material, such as increases in Born effective charge, increases in deformation potential or reductions in Young's modulus in order to increase the Fröhlich and/or acoustic coupling constants. These could not be realized simply through changing the defect density or grain size. XRD measurements showed that adding SC to the precursor solution had no obvious change on the crystal structure or preferred orientation.

Will it be useful to investigate Sb_2S_3 with no midgap states obtained by the incorporation of a few atomic% of isovalent cation into Sb_2S_3 reported recently (Miliaieva et al. 2025) to check if the polaronic signatures still persist even without midgap defects? And mainly, what effect does the absence of Sb_2S_3 midgap states have on the solar cell performance parameters?

Response: We thank the reviewer for these comments, and for pointing out the suggested works.

(1) We read with interest the work from Miliaieva et al., which used DFT calculations to show that the midgap defect states in Sb_2S_3 can be eliminated by incorporating the isovalent cation Bi^{3+} into Sb_2S_3. This provides a novel strategy for healing deep defects, thus possibly mitigating non-radiative recombination and tuning electrical conductivity in Sb_2S_3 (*J. Phys. Energy* 2025, 7, 035012). It is generally accepted that the absence of midgap states would reduce charge-carrier recombination and further enhance solar cell performance.

(2) That being said, the above-mentioned paper (*J. Phys. Energy* 2025, 7, 035012) used computational methods to suggest that mid-gap states in Sb_2S_3 could be suppressed through Bi alloying. It would certainly be interesting to experimentally demonstrate this and investigate the effect on the nature of polarons formed in Sb_2S_3. We are open to exploring this, but we believe this to be outside the scope of the present work. Experimentally, it is well known that

it is very difficult to obtain defect-free Sb_2S_3 absorber films (*Chem. Eng. J.* 2022, 446, 137400). Even if mid-gap traps are predicted to be eliminated by Bi alloying, we may introduce other types of defects, or we could completely change the growth mode, requiring many cycles of optimization before we can achieve working devices again. Investigating Bi-alloyed Sb_2S_3 therefore is a separate project and paper in its own right. Moreover, our new temperature-dependent TOF mobility data provides strong evidence in support of large polaron formation, and we believe that our current Sb_2S_3 films are adequate for this work.

3. The work shows only a modest improvement in Sb_2S_3 V_{OC}, 1.6% higher than the recently reported V_{OC} of 0.811 V (Yu et al. 2025). Solar cell maximum efficiency of 7.67% is comparable to the reported before. There is no novelty in the fabrication procedure, characterisation methods used, or the problematics. These put the work among incremental works.

Response: We are grateful for the opportunity to clarify the novelty and impact of our work.

The key conceptual breakthrough in our work is the experimental demonstration that the performance of Sb_2S_3 photovoltaics is limited by defects rather than by self-trapping. We explained already in our answers to the earlier questions that there has been significant debate in the community about whether or not the performance of Sb_2S_3 is limited by self-trapping. As we explained in our answer to the first question, if self-trapping did take place, as proposed by Yang *et al.*, the potential to improve the performance of Sb_2S_3 would be significantly limited, since self-trapping is an intrinsic process, and we would not expect a V_{OC} surpassing 0.8 V. The work by Yu *et al.* mentioned (*Materials Today Energy*, 2025, 54, 102083) is focussed on defect engineering and does not discuss the effects of self-trapping. In our work, we not only achieve a record V_{OC}, but much more importantly, we provide direct experimental evidence of band-like transport in Sb_2S_3. The new temperature-dependent mobility data strengthens our experimental evidence, in addition to the defect measurements and calculations in our paper that show the strong correlation between V_{OC} and reduced trap density. The results from our work are critical for the wider field by showing that it is possible to improve the performance of Sb_2S_3 towards the radiative limit through careful defect engineering, and that progress has not stalled. This fundamentally changes the future direction for this material, and we hope will reignite interest from the wider photovoltaics community, since having many groups working

on a technology is critical for accelerating progress. Experimentally proof of band-like transport is also critical for the wider community working on developing heavy pnictogen-based semiconductors as nontoxic, stable alternatives to lead-halide perovskites. Carrier localization has been widely found with these materials, and identifying Sb_2S_3 as a compound that deviates from this common feature makes it an important material to study further to identify design principles for achieving band-like transport in novel solar absorbers.

To clarify these points, we have changed the start of our discussion section (Page 24, highlighted in blue).

"There have been substantial efforts over the past decade to develop light absorbers that can replicate the exceptional optoelectronic properties of lead-halide perovskites and overcome their toxicity and stability limitations[72, 73]*. A critical limitation of many of these perovskite-inspired materials (especially with Sb- and Bi-based compounds) is carrier localization, which has been so widely found that it was being labelled a hallmark of these materials*[74]*. This prevents their performance from approaching that of lead-halide perovskite solar cells, despite exhibiting improved stability*[72]*. Sb_2S_3 was thought to be an example of a material with this limitation*[29]*. Despite continuous improvements in performance of Sb_2S_3 solar cells over the past decade, device optimization of these materials has mostly been based on trial-error materials engineering approaches. A more general understanding of the physics behind charge-carrier generation, recombination and transport is an indispensable prerequisite for revealing fundamental limits and prospects of Sb_2S_3 for optoelectronic applications, as well as offering essential feedback for further device improvements. Our experimental and computational results here demonstrate that the previously-proposed hypothesis of self-trapping in Sb_2S_3 is not present, showing that Sb_2S_3 avoids the carrier localization limitations prevalent among other Sb- and Bi-based solar absorbers, and that this material has potential to reach significantly higher efficiencies. This motivates detailed investigations to understand the underlying principles behind its band-like transport, which can be generalized to other classes of perovskite-inspired materials."*

4. The absorber thickness varies strongly across samples (e.g., ~506 nm control vs ~318 nm for 4mM-SC; and down to ~116 nm for 8mM-SC). This affects optical generation, electric-field distribution, series resistance, and any thickness-scaled transport analysis. It would be

beneficial for all thickness-sensitive electrical comparisons (e.g. SCLC) to use appropriate normalisation and/or a control sample thickness-matched to the test samples, as demonstrated in literature (e.g., Kotadiya et al., 2019) or by preparing thickness-matched films via growth time adjustment.

Response: We thank the reviewer for these suggestions. In our work, the optimized absorber thicknesses for the control group of devices and the SC group of devices are different. Aiming to explore the underlying mechanisms explaining the performance difference for real devices, we directly employed the best-performing control device and 4mM-SC device to investigate device physics, although they do have different absorber thicknesses. We took care to account for the influence of film thickness on the electrical properties (such as SCLC and mobility measurements), elaborated as follows.

(1) In the above-mentioned literature (*Nat. Photonics* 2019, 13, 765-769), Kotadiya et al. investigated the layer-thickness dependent mobility and trapping parameters for organic materials based on SCLC measurements, and obtained the same charge-transport parameters for different layer thicknesses. In another work from Kotadiya *et al.* (*Nat. Mater*. 2018, 17, 329-334), they also showed that the SCLC curves are independent of absorber thickness based on four different organic materials with varying device thickness. Even so, according to the reviewer's suggestions, we prepared thickness-matched films for SCLC measurements and updated the SCLC curves as shown in Figure 6d. It is revealed the trap state density in the control devices is higher than that of the 4mM-SC device, consistent with the previous results.

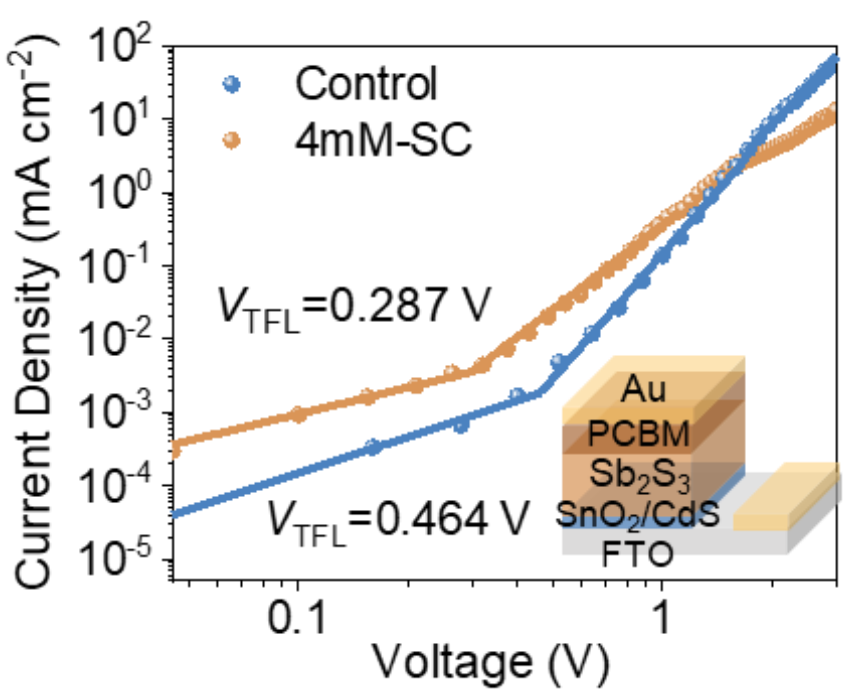

Figure 6d. Space-charge-limited current density (SCLC) measurements of the control and 4mM-SC devices based on the electron-only device, with the configuration: FTO/SnO_2/CdS/Sb_2S_3/PCBM/Au.

(2) We confirm that we accounted for film thicknesses in the new temperature-dependent mobility measurements that we added to the revised manuscript. Sb_2S_3 film thickness is

required to calculate the mobility from time-of-flight measurements, based on the equation $\mu = \frac{d^2}{\tau_{tr} V}$, where μ is hole mobility, V is the driving voltage, and d is film thickness. In this work, the thickness of Sb_2S_3 films was measured using a KLA-Tencor D100 step profilometer based on three measurements for each sample.

Reviewer 2:

The manuscript reports a record V_{OC} of 824 mV in Sb_2S_3 solar cells, surpassing the ~800 mV limit previously proposed by Yang et al. (Nature Commun. 2019). While the experimental achievement is commendable, the theoretical discussion regarding the origin of this "limit" and the distinction between self-trapping (polarons) and defect-trapping is logically flawed and incomplete.

Response: We thank the reviewer for their time in going over our paper, and for noting that the experimental achievement of this work is commendable. We have gone through all comments raised, and collected new experimental evidence to address these points, as detailed below.

1. The authors frame their work as "breaking" the 0.8 V limit derived by Yang et al. It is crucial to acknowledge that Yang's 0.8 V limit was calculated based on the assumption that the observed ~0.6 eV Stokes shift originates from intrinsic self-trapping (polarons), which would represent a fundamental physical limit. By achieving 0.824 V, the authors experimentally demonstrate that the voltage loss is not fundamental but technological (i.e., defect-mediated). This implies that Yang's initial assignment of the Stokes shift to intrinsic polarons was likely incorrect. Therefore, the authors are not "breaking a fundamental barrier" but rather disproving a hypothesis. The manuscript must be rewritten to explicitly discuss the origin of Yang's 0.8 V value and state that the current results invalidate the assumption that intrinsic self-trapping is the dominant recombination pathway.

Response: We thank the reviewer for these valuable comments.

(1) We agree with the reviewer that we are not breaking a fundamental limit but rather exceeding a long-standing threshold of 800 mV for the V_{OC} that Sb_2S_3 devices have approached

over past few decades. To date, most of Sb_2S_3 solar cells show V_{OC} within the range of 550-770 mV, much smaller than other photovoltaic devices based on absorbers with similar or smaller bandgaps (*Science* 2016, 352, 6283; *Prog Photovoltaics* 2023, 31, 651-663). In this work, we set a new record V_{OC} for Sb_2S_3 solar cells, which is important, since the performance of Sb_2S_3 photovoltaics, as well as many other chalcogenide photovoltaics, is primarily limited by the large V_{OC} deficit.

We have accordingly gone back through the entire paper to make this clear. This includes changing the title of paper to "*Exceeding the 800 mV open-circuit voltage threshold in antimony sulfide photovoltaics*".

(2) In the revised manuscript, we added more explanations to explicitly discuss the origin of the 0.8 V value for the V_{OC} threshold proposed by Yang *et al.* (Page 4-5, highlighted in blue):

"This mechanism is linked to the experimental observation of linearly polarized photoluminescence (PL) that was 600 meV red-shifted compared to the optical bandgap of Sb_2S_3[29]. These features were attributed to emission from STE states, and from this data, it was hypothesized that the maximum V_{OC} would be 600 mV below the radiative limit of 1.4 V for a 1.7 eV bandgap semiconductor; that is, the maximum V_{OC} would only be 800 mV for Sb_2S_3 if this hypothesis were true[29]. Similar observations were made for Sb_2Se_3, where it was also proposed that self-trapping fundamentally limits the V_{OC}[30]. Given that self-trapping is an intrinsic process, materials engineering to lower defect densities would not allow the V_{OC} and efficiency to exceed the limits set by this process, thus inhibiting future progress with these materials. Indeed, a V_{OC} of 800 mV is the value most Sb_2S_3 photovoltaics have asymptotically approached over the past decade of efforts made with this material (Supplementary Table 1)"

(3) As part of this revision, we have collected further experimental evidence to disprove the hypothesis of self-trapping in Sb_2S_3. That is, we measured the temperature dependence of the charge-carrier mobility in the Sb_2S_3 films (see Fig. 6a below). If small polarons formed, charge-carriers would be transported via a hopping mechanism, meaning that the mobility would increase with increasing temperature. By contrast, if large polarons instead formed, the delocalized nature of the wavefunction means that transport is more band-like, and mobility would decrease with increasing temperature. This clear difference in the temperature-dependence of mobility allows us to differentiate between large and small polarons. As can be seen in the new Fig. 6a we added to this revision (copied below), the mobility of Sb_2S_3

decreases with increasing temperature. The dominant charge-carriers in Sb_2S_3 therefore are clearly large polarons.

In the revised manuscript, we have provided a detailed discussion on the mobility data (Page 20, highlighted in blue):

"To verify the formation of delocalized large polarons instead of small polarons in Sb_2S_3, we determined the temperature dependence of the mobility through time of fight (TOF) measurements[62]. It is known that large polarons show a reduction in the overall mobility with increasing temperature because of increased phonon scattering. On the other hand, if small polarons dominate charge-carrier transport, the mobility should increase as the temperature increases because small polaron formation changes the transport mechanism to hopping-based. The device structure for TOF measurements was FTO/Sb_2S_3/Au, and the measurement details can be found in Methods. Note that CBD was unable to grow compact films directly on the FTO substrates, and so we used closed-space sublimation (CSS) to form continuous thin films that avoid shunting; we would not expect any differences in the trends in mobility with temperature for CBD vs. CSS-grown Sb_2S_3. Fig. 6a shows the obtained mobilities of Sb_2S_3 at different temperatures ranging from 100 to 400 K based on the bilogarithmic plots of transient current vs. transit time (Supplementary Fig. 19). The observation of an obvious decrease in mobility with an increase in temperature is consistent with the behavior of large polarons."

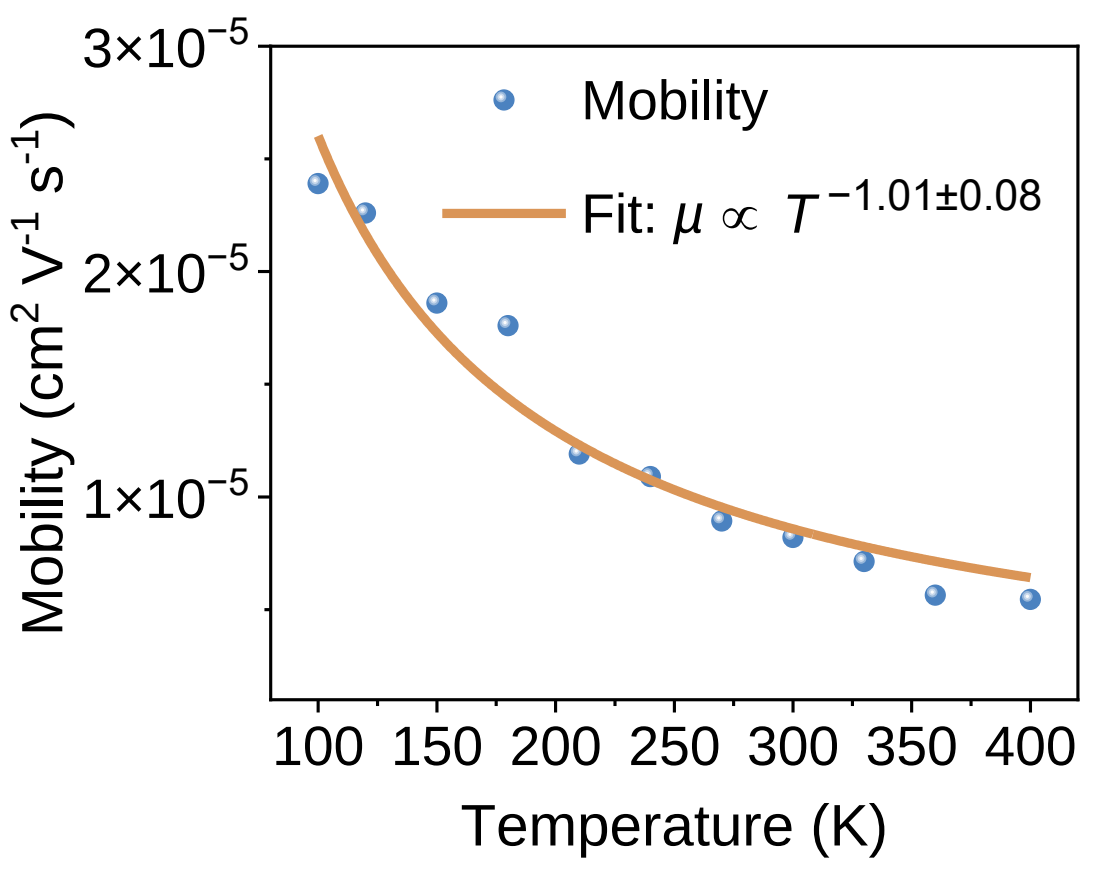

Fig. 6a Temperature-dependent mobility of Sb_2S_3 films determined using time of flight (TOF) measurements, fitted with a power law model that indicates $\mu \propto T^{-1.01\pm0.08}$. Here ±0.08 is the uncertainty of the exponent obtained by fitting the data across all temperatures.

2. The authors perform DFT (HSE) calculations to identify point defects (V_S, Sb_S) and assign them to the DLTS signals. However, both defect states and self-trapped polaron states can be calculated using hybrid functionals. To conclusively claim that they have overcome self-trapping by reducing defects, the authors must theoretically compare the two. The authors should perform calculations for the self-trapped hole/electron states (polarons) in Sb_2S_3. By comparing the formation energies and capture cross-sections of the Polaron state *vs.* the Defect state, the authors can theoretically demonstrate which mechanism is energetically favorable. Without calculating the polaron state, the conclusion that defects dominate is theoretically incomplete.

Response: We thank the reviewer for these helpful suggestions. We would like to clarify that our conclusion is not that defect states are energetically favourable compared to self-trapped polarons. Rather, our result shows that stable self-trapped excitons or small polarons do not form in Sb_2S_3. A comparison between defect states and self-trapped excitons is only meaningful if both co-exist as stable states. In our previous first-principles study (*ACS Energy Lett.* 2022, 7, 2954-2960), we explicitly attempted to construct small electron and hole polaron configurations in large supercell hybrid-DFT calculations by introducing symmetry breaking and localized initial distortions. In all cases, the carriers relaxed back to delocalized states, and no stable small polarons were obtained. As a result, their formation energies and capture cross sections cannot be meaningfully defined.

3. The authors attribute the energy discrepancy (approx. 0.1-0.2 eV) between the calculated defect levels and the experimental DLTS signals (H1/H2) to "temperature effects." This explanation appears speculative and is cited without specific computational support for this material system. Given the high precision claimed in the rest of the work, attributing a significant energy shift to thermal effects without calculation is scientifically loose. Furthermore, if the previous "0.8 V limit" was indeed due to defects (misidentified as polarons by Yang), the theory must explain the spectroscopic origin. The authors should discuss whether the calculated relaxation energy (Frank-Condon shift) of their identified defects matches the large ~0.6 eV Stokes shift observed in previous literature, which would provide a solid link between their defect theory and the historical spectral data.

Response: We thank the reviewer for raising this point. While explicit calculations of finite-temperature defect thermodynamics are computationally prohibitive for large hybrid-DFT supercells, we note that bulk Sb_2S_3 has pronounced temperature-dependent changes in bandgap. Theoretical studies (Liu Y, *et al. Phys. Rev. Mater.* 2023, 7(8), 085401) report a temperature-induced bandgap shift of ~0.2 eV in Sb_2S_3. The resulting shift in transition level positions is therefore not unexpected. Recent studies have shown that accurately capturing finite-temperature effects on defect energetics requires statistical sampling of vibrational and configurational contributions, which is currently only tractable using machine-learned force fields (Mosquera-Lois *et al.*, *Chem. Sci.* 2025, 16, 8878-8888; *PRX Energy* 2025, 4, 043008). These corrections, while beyond the scope of the present work, are not expected to affect the relative level ordering or the assignment of defect species.

Moreover, our calculated defect configurations exhibit substantial lattice relaxation upon charge transition, yielding sizable relaxation (Franck–Condon) energies that are qualitatively consistent with the large Stokes shifts reported previously, providing a coherent defect-based explanation of the spectroscopic signatures.

We have modified the discussion in the revised manuscript (Page 20, highlighted in blue):

"*One possible origin of the ~0.1-0.2 eV discrepancy between theoretical predictions and experimental observations is the temperature dependence of the host band structure, which is not considered in our electronic structure calculations. Theoretical studies have shown that the bandgap of Sb_2S_3 shifts by ~0.2 eV between 0 K and room temperature due to lattice expansion and electron–phonon renormalization. Such deviations are not expected to affect the relative level ordering or defect assignments.*"

Reviewer 3:

In "Breaking the 800 mV open-circuit voltage barrier in antimony sulfide photovoltaics", a new record *V*oc is reported for Sb_2S_3, following optimization of the chemical bath deposition process by addition of a complexing agent.

Breaking the 800 mV Voc barrier is an important milestone. The paper is full of interesting data from (advanced) characterization and (advanced) first-principles calculations. It will be very valuable for the $Sb_2(S,Se)_3$ community.

Response: We thank the reviewer for their time in going over our paper, and for recognizing the significance of this work in exceeding the 800 mV V_{OC} threshold for Sb_2S_3 solar cells, driven by the advances we have made in suppressing defect-induced recombination.

May main criticism is related to some overstatements and to some potentially erroneous conclusions.

• The authors repeatedly emphasize that the demonstration of a 824 mV *V*oc is a sign that Sb_2S_3 cells are not intrinsically limited by self-trapped excitons. This is based on a *V*oc limit of 0.8 V for a device limited by self-trapped excitons. Going back to the original source, this 0.8 V limit was just a rough estimate rather than an exact value (it is also given with just one significant digit, making it equal to the new record *V*oc). While a new record Voc is a great achievement and it shows that progress on Sb_2S_3 has not stalled, it does not prove that Sb_2S_3 are not limited by self-trapped excitons. These statements are misleading and should be removed.

Response: We thank the reviewer for pointing this out, and agree that demonstrating a V_{OC} exceeding the threshold proposed by Yang *et al.* is not direct proof that self-trapping does not occur in Sb_2S_3. We have therefore measured the temperature dependence of the charge-carrier mobility of the Sb_2S_3 films (Fig. 6a and Supplementary Fig. 19). These new data are copied out below for convenience, and clearly show a decrease in mobility with increasing temperature. This is fully consistent with the behaviour of large polarons, rather than small polarons (which would increase in mobility with temperature, due to a hopping-based transport mechanism).

We have modified the discussion in the main text (Page 20, highlighted in blue):

"To verify the formation of delocalized large polarons instead of small polarons in Sb_2S_3, we determined the temperature dependence of the mobility through time of fight (TOF) measurements[62]. It is known that large polarons show a reduction in the overall mobility with increasing temperature because of increased phonon scattering. On the other hand, if small

polarons dominate charge-carrier transport, the mobility should increase as the temperature increases because small polaron formation changes the transport mechanism to hopping-based. The device structure for TOF measurements was FTO/Sb_2S_3/Au, and the measurement details can be found in Methods. Note that CBD was unable to grow compact films directly on the FTO substrates, and so we used closed-space sublimation (CSS) to form continuous thin films that avoid shunting; we would not expect any differences in the trends in mobility with temperature for CBD vs. CSS-grown Sb_2S_3. Fig. 6a shows the obtained mobilities of Sb_2S_3 at different temperatures ranging from 100 to 400 K based on the bilogarithmic plots of transient current vs. transit time (Supplementary Fig. 19). The observation of an obvious decrease in mobility with an increase in temperature is consistent with the behavior of large polarons."

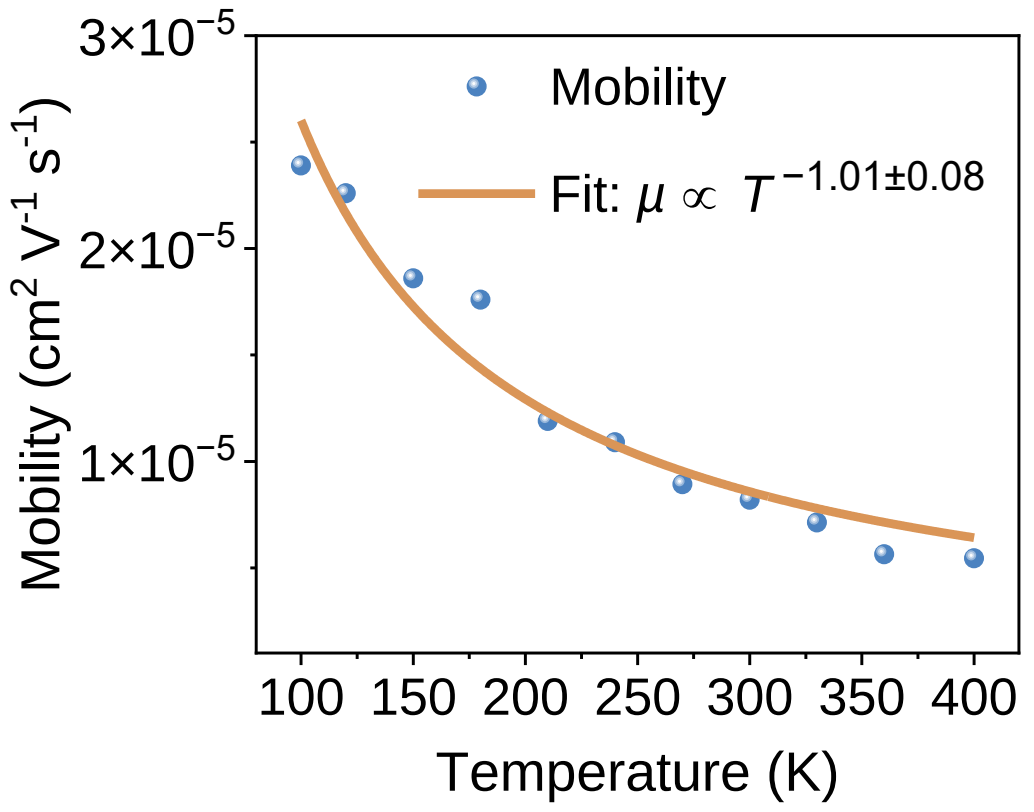

Fig. 6a Temperature-dependent mobility of Sb_2S_3 films determined using time of flight (TOF) measurements, fitted with a power law model that indicates $\mu \propto T^{-1.01\pm0.08}$. Here ±0.08 is the uncertainty of the exponent obtained by fitting the data across all temperatures.

In addition, we have gone back over the entire paper to ensure that we do not imply that exceeding 800 mV is proof of avoiding self-trapping in this system, rather is simply correlative.

• Page 10. "In polycrystalline films, recombination losses are largely caused by GBs etc.". This is a very hand-waving argument that is often not true. There are many published examples of CIGS solar cells where smaller grains gave more efficient devices because the dominant recombination mechanism is not at the grain boundaries. I cannot find any evidence in this paper to link the improved performance to grain boundaries, even from the kelvin probe measurements. On the contrary, the grain boundaries are already large in the control film (much larger than the film thickness) and transport is in the out-of-plane direction. Yes, there is a

correlation between GB density and efficiency as stated on page 22, line 546, but there does seem to be a cause-effect relationship.

Response: We agree with the reviewer that the exact roles of GBs on device performance for material systems, such as CIGS, still remains controversial.

(1) GBs play a critical role on the performance of solar cells based on polycrystalline absorber layers, especially materials with small grains. Many studies have shown that GBs are detrimental for device performance due to increased non-radiative recombination compared to the bulk of the grain. This has been found in a variety of materials, including multi-crystalline silicon (*Phys. Status Solidi A* 2013, *210*, 641; *J. Appl. Phys.* 1975, *46*, 5247), halide perovskites (*Adv. Mater*. 2016, 28, 917-922; *Joule* 2024, 8, 913-921), selenium (*Nat. Energy* 2026, https://doi.org/10.1038/s41560-025-01939-x), and Sb_2S_3 (*Adv. Mater*. 2024, 36, 2305841). However, the roles of GBs in some thin film solar cells such as CIGS remain controversial. Some of these studies (*Thin Solid Films* 2009, *517*, 2545; *J. Appl. Phys.* 2014, *115*, 014504; *Chem. Eng. J*. 2025, 522, 167532) indicate that the GBs are characterized by enhanced non-radiative recombination and are therefore detrimental to the performance of CIGS solar cells. On the contrary, other studies (*Thin Solid Films* 2011, *519*, 7341; *Appl. Phys. Lett.* 2004, *85*, 2625), indicate that the GBs in the absorber layer have a benign effect on the CIGS cell efficiency, attributed to type inversion at GBs (downward band bending) (*ACS Appl. Mater. Interfaces* 2018, *10*, 14759). A recent investigation from Raghuwanshi et al reveals GBs with all the three traits (benign, detrimental and neutral) to co-exist in CIGS (*Adv. Funct. Mater.* 2020, *30*, 2001046).

(2) The underlying mechanisms responsible for the controversial role of GBs might be associated with the complex phase diagram in the multi-component CIGS system. The grain size largely depends on the composition. For example, the higher the ratio of Cu/(In+Ga), the larger the grain size; however, this leads to the formation of Cu-rich inclusions, such as copper selenide, which would cause a reduction in device performance, and could even short circuit devices (*Acta Mater*. 2010, 58, 3468-3476; *Thin Solid Films* 2011, 519, 7228-7231). In addition, specific chemical treatments of CIGS films, such as NaF precursors, often contribute to the enhanced device performance. Here NaF precursors have been demonstrated to modify the growth kinetics of CIGS and result in a reduction in grain size and a slightly enhanced Ga-gradient through the absorber layer (*Thin Solid Films* 2009, 517, 2187-2190; *Appl. Phys. Lett.* 2004, 84, 1129-1131). That is, the increased Ga composition gradient which helps to enhance

the performance of CIGS devices is accompanied by the reduced grain size with increasing Na concentration (*J. Appl. Phys.* 2009, 106, 034908).

Therefore, although it may appear that smaller grains in CIGS led to more efficient devices, a comprehensive consideration of a variety of factors is needed to explain the enhanced performance. To obtain highly efficient CIGS devices, it is necessary to balance the material composition, cascade band structure, film crystallinity and orientation, and even the adhesion to the substrate.

(3) For Sb_2S_3 with a simple binary composition, the obtained Sb_2S_3 films show good crystallinity and film morphology, and we observed each individual grain to be single-crystalline, as confirmed by FIB-TEM (Figure 3). Through comprehensive characterization and analysis, including DLTS, TAS, as well as computations, the 4mM-Sb_2S_3 films with larger grain size are revealed to show reduced defect density and non-radiative recombination compared to the control sample. The overall reduction in trap state density that we measured is correlated with the reduction in GB density. It is reasonable to deduce that the recombination losses in Sb_2S_3 are closely associated with GBs, where dangling or wrong bonds proliferate, including the GBs located at the film interface and film interior. This is consistent with previous work (*Light: Sci. Appl.* 2024, 13, 281; *Results in Surfaces and Interfaces* 2024, 16, 100251).

(4) It is indeed the case that the control Sb_2S_3 film already has grain size larger than the film thickness, as the reviewer mentioned. In spite of this, the Sb_2S_3-based devices would also inevitably involve the negative effects of GBs with dangling bonds at the surface and interior of polycrystalline films, which would be detrimental to device performance. Moreover, the influence of GBs was found to be most detrimental when placed in the space charge region close to the heterointerface, such as the Sb_2S_3/Spiro-OMeTAD heterointerface in our case (*J. Appl. Phys.* 2008, 104, 083706). There are also some studies demonstrating similar negative impacts of GBs in perovskite solar cells, in which grains were also large enough to extend beyond the entire absorber thickness (*ACS Energy Lett.* 2017, 2, 12, 2727-2733; *Adv. Mater.* 2016, 28, 917-922; *Joule* 2024, 8, 913-921).

(5) Based on the above discussion, we suggest a correlation between GB density and device efficiency, and we have accordingly modified our statements (Page 10, highlighted in blue):

"*It is generally accepted that, in polycrystalline films, dangling or wrong bonds proliferate at*

GBs, which would result in increased non-radiative recombination losses and be detrimental for device performance. Hence, increased device performance with polycrystalline absorbers would often be correlated with reduced GB density."

• It is stated that film thickness changes quite a lot between the control sample (500 nm) and the samples where SC was used (down to 116 nm). The PV efficiency differences between these samples are not massive (Fig. 2). So, there is a possibility that the efficiency changes are heavily influenced by the different thicknesses. The authors should present convincing evidence (such as a thickness study on the control sample or on the 4mM-SC sample) showing that the variable film thickness is not the main contributor to the efficiency trends.

Response: According to the reviewer's suggestion, we investigated the effect of film thickness on the performance of Sb_2S_3 solar cells in the control sample, and our new data is shown in Supplementary Fig. 17. The results show that the control sample obtained with the 8 h CBD delivers the highest PCE, implying that the optimal device performance of the control sample is much lower than that of the SC-Sb_2S_3 samples. This confirms that the incorporation of SC in the CBD precursor indeed contributes to the performance enhancement of Sb_2S_3 solar cells, which is proposed to be closely associated with the suppression of defect-induced charge-carrier recombination.

In the revised main text, we have provided corresponding descriptions (Page 14, highlighted in blue):

"Furthermore, since the incorporation of SC leads to a change in Sb_2S_3 film thickness, we also investigate how the performance of Sb_2S_3 solar cells depends on absorber thickness (Supplementary Fig. 17). It is shown that the optimal device PCE of the control sample is still much lower than that of the SC-Sb_2S_3 samples, confirming that the variable film thickness caused by the use of SC is not the dominant contributor to the efficiency enhancements obtained here."

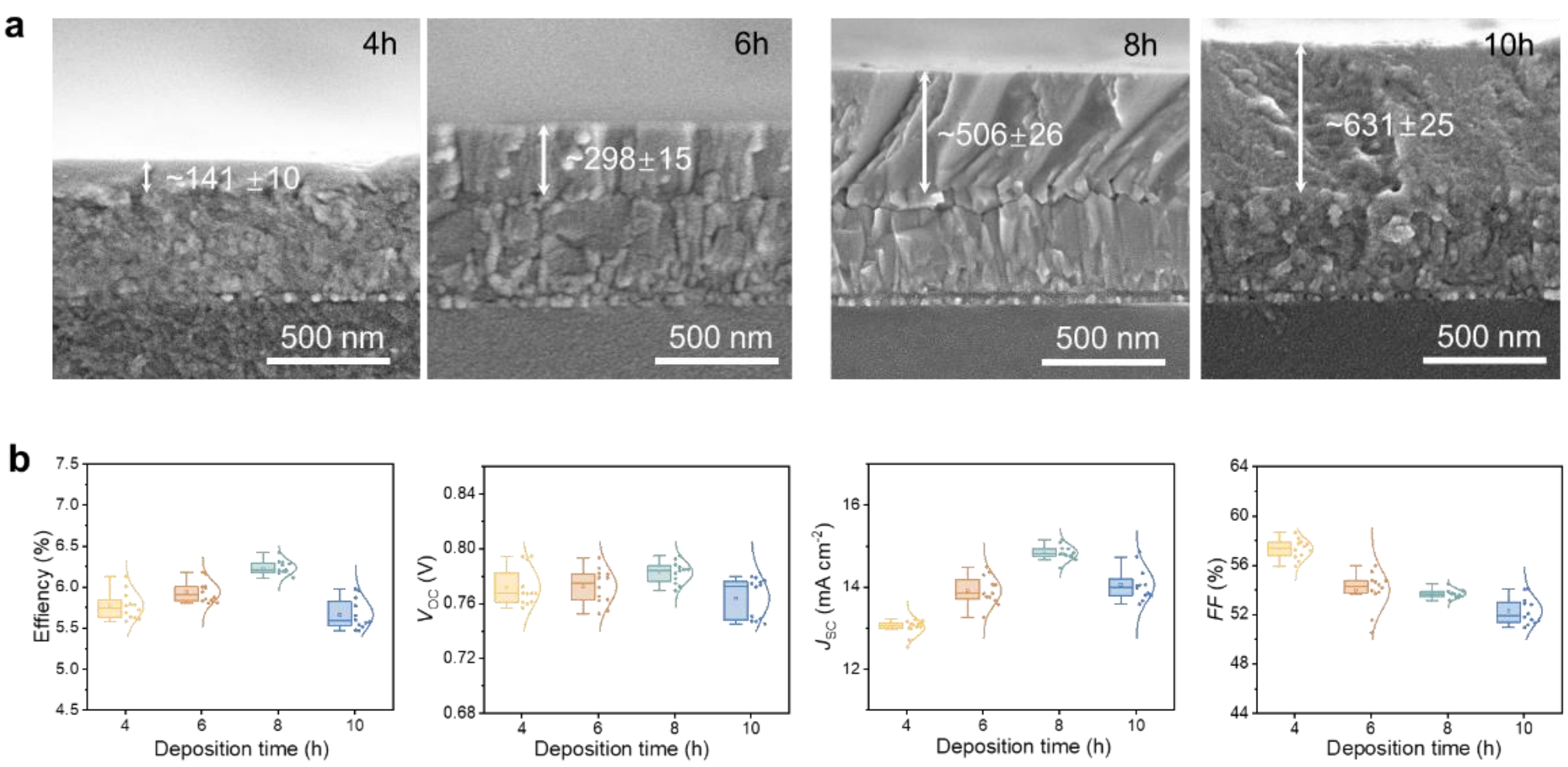

Supplementary Figure 17. a Cross-sectional SEM images of the control Sb_2S_3 films obtained with different CBD time (4 h, 6 h, 8 h and 10 h). **b** The statistics of the device performance of control Sb_2S_3 solar cells obtained with different CBD time. The results show that the control sample obtained with the 8 h CBD delivers the highest PCE, implying that the optimal device performance of the control sample is much lower than that of the SC-Sb_2S_3 samples.

• Page 12. "The reduced fluctuation of the current intensity for the 4mM-SC Sb_2S_3 film should be conducive to the local photocurrent generation and collection, which might effectively suppress charge-carrier recombination at GBs". I just don't understand this statement. Please clarify.

Response: We thank the reviewer for raising this. To explain: here, the current intensity obtained from c-AFM measurements refers to the magnitude of the local current passing through the sample when a bias voltage is applied between the probe and the sample, and corresponds to the conductivity of sample area beneath the probe. The reduced fluctuation in this current is consistent with the lowered surface roughness at the film surface from AFM measurement. We suggest that this reduced film surface roughness could lead to reduced interface recombination in devices due to a reduced interfacial area at the heterojunction. Reading back over the entire passage on AFM again, we believe it best to delete this specific sentence to avoid being speculative; the sentences that we kept in the paper are adequate for conveying our message here (Page 12, highlighted in blue):

"*Atomic force microscopy (AFM) maps of the morphology of the Sb_2S_3 films (Supplementary Fig. 10) confirm that the 4mM-SC film shows larger grain sizes with fewer GBs than the control.*

Furthermore, the addition of SC leads to a reduction in the root-mean-square roughness from 21.9 nm (control sample) to 16.3 nm (4mM-SC sample). The reduced surface roughness of the absorber films would favor the formation of an improved heterojunction between Sb_2S_3 and solution-processed HTL on top. Conductive-atomic force microscopy (c-AFM) images, with the corresponding morphology and current maps (Supplementary Fig. 11) reveal that, compared to the control sample, the 4mM-SC films have more uniform and slightly enhanced electrical conductivity over the 5 × 5 μm² area measured."

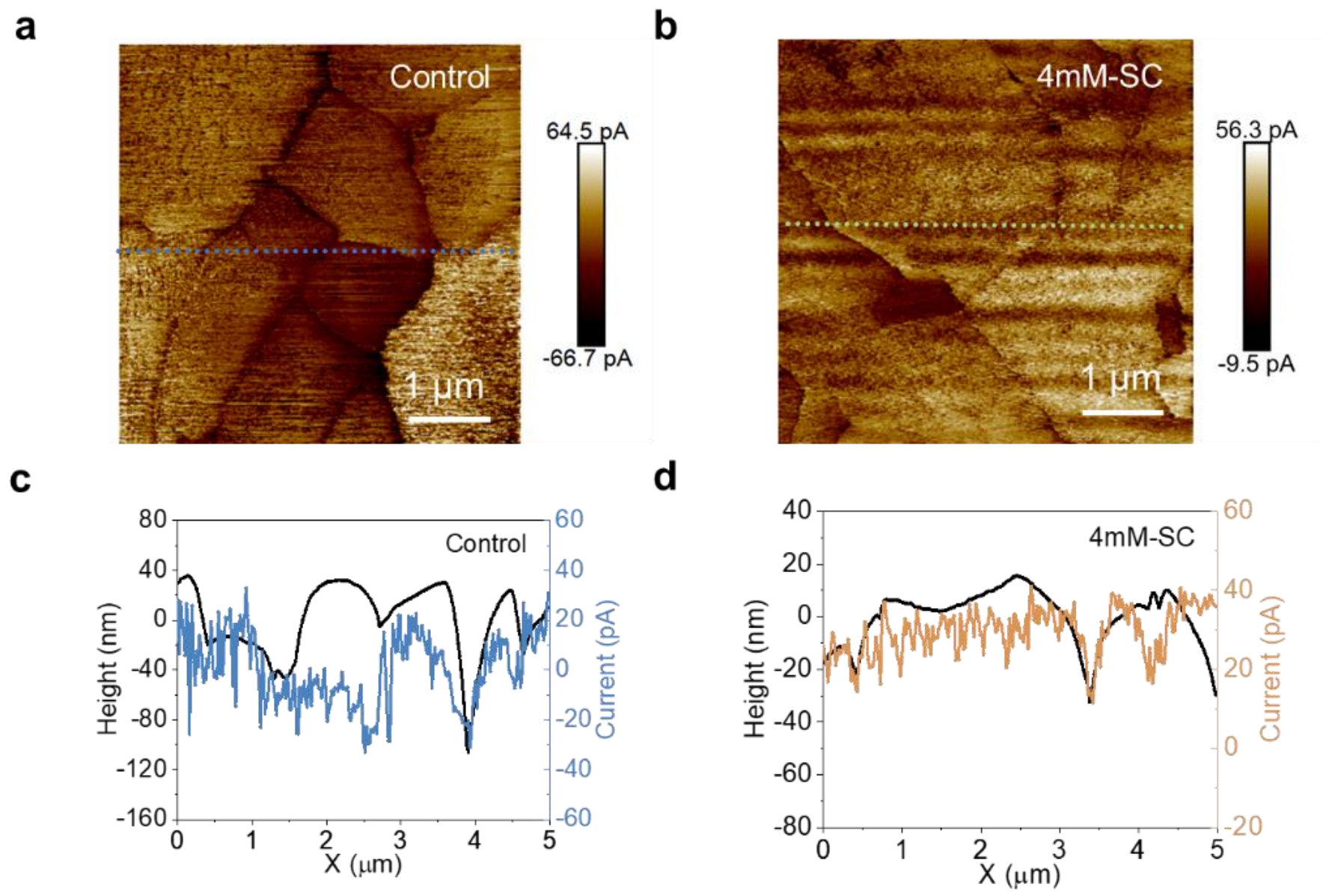

Supplementary Figure 11. a, b c-AFM images and **c, d** corresponding height profile and current analysis of the control film sample and 4mM-SC Sb_2S_3 film sample. The films suffer from evident current fluctuations at the GBs.

• Page 20. The authors give a calculated HSE band gap of 1.79 eV and a maximum *V*oc of 1.70 V in the radiative limit. This *V*oc seems to violate physics (higher than expected from Shockley-Queisser limit). Please clarify.

Response: The experimentally-measured bandgap of Sb_2S_3 is 1.7-1.8 eV, which is very close to the HSE-calculated bandgap of 1.79 eV.

The reported V_{OC}^{rad}=1.70V does not violate physics. It arises from our radiative detailed-balance calculation implemented in TrapLimitedConversion package, which uses the realistic

absorption coefficient of Sb_2S_3, rather than the idealized step-function absorptance assumed in the SQ model.

In practice, semiconductors absorb weakly near the band edge, and the absorption increases gradually with photon energy. This suppresses the radiative dark current J_0 more strongly than the photocurrent J_{sc}. Physically, thermal emission at 300 K is concentrated close to the band edge, where absorption is weakest, so recombination is strongly reduced, whereas the solar spectrum spans higher energies where absorption is already strong. Because V_{OC} depends on the ratio J_{sc}/J_0, this asymmetric suppression naturally leads to a slightly higher radiative-limit voltage compared to the ideal SQ estimate. For a 1.79 eV bandgap, a radiative loss of ~0.09 V is therefore physically reasonable.

We have clarified this point in the Methods (Page 29, highlighted in blue):

"The photon absorptivity $a(E;W)$ describes the probability of absorption as a function of photon energy. In the Shockley-Queisser model, it is idealised as a step-like function at the bandgap. In our calculation, the absorptivity is defined as $a(E;W) = 1 - e^{-2\alpha(E)W}$, where $\alpha(E)$ is the optical absorption coefficient of Sb_2S_3 obtained from first-principles calculations using the HSE06 functional [83]."

That being said, upon reviewing the paper, we decided that this specific point may be distracting to general readers. We have therefore moved this discussion of the calculated trap-limited maximum V_{OC} and PCE to the supplementary information (Supplementary Note 4). The main text now only states our calculated trap-limited maximum V_{OC}, which shows that there is plenty of room for further improvements that are achievable through defect engineering. The modified section on page 20 is quoted below:

"*Based on the identified defects ($V_{S(3)}$ and $Sb_{S(2)}$), and their measured trap densities and capture cross-sections, we predicted the trap-limited V_{OC} accounting for both radiative (band-to-band) and defect-mediated non-radiative recombination processes (Supplementary Note 4 and Supplementary Fig. 18). The predicted V_{OC} reaches 1.15 V for the control sample and 1.26 V for the 4mM-SC sample, both exceeding the 0.8 V threshold. The predicted improvement in performance and V_{OC} by lowering trap density for the 4 mM-SC sample is qualitatively consistent with the improvement observed experimentally. At the same time, these predictions show that other loss processes are present, beyond non-radiative losses from $V_{S(3)}$ and $Sb_{S(2)}$.*"

• C-V measurements are presented and values of built in voltage are given. However, the films are relatively thin and low carrier concentrations are expected from the UPS data in the SI (and for Sb_2S_3 in general). For 300 nm film thickness, the carrier concentration would need to be above E16 cm-3 for the analysis to be valid (avoiding geometric capacitance effects and charge injection). Reference: Figure 1c in 10.1126/science.abd8014. It seems likely that the present Sb_2S_3 films have lower carrier concentration. In that case, the analysis would not be valid. Please clarify.

Response: We thank the reviewer for pointing out this. After carefully checking the applicable condition of C-V measurements, we agree with the reviewer's statement that the C-V analysis might not be valid for Sb_2S_3 with low carrier concentration. Although there are many prior reports that the carrier concentration of Sb_2S_3 is in the 10^{16} cm^{-3} range based on the C-V characterization (*ACS Appl. Mater. Interfaces* 2018, 10, 36, 30314-30321; *Sci. Bull.* 2019, 64, 136-141), the real carrier concentration of Sb_2S_3 is probably much lower than that detected by C-V measurements. This requires further study.

In this work, in order to avoid misleading and make the description more accurate, we have deleted the C-V characterization and corresponding analysis. This does not influence the novelty of this work and the discussion of results.

Minor comments:

• Page 6. "SC was used as a complexing additive etc.". Already at this point, I suggest making a reference to the section in the SI where an (interesting) interpretation of the CBD deposition mechanism in the presence of SC is given.

Response: We thank the reviewer for the reminder. In the revised version, we have made a reference to Supplementary Note 1 in the SI for this statement (Page 6-7, highlighted in blue):

"*An interpretation of the deposition mechanism for CBD in the presence of SC is detailed in Supplementary Note 1*".

• The whole preliminary description of DLTS (page 14 and 15) is useful but very long and it's more like textbook information. I suggest moving it to the SI

Response: According the reviewer's suggestion, we have moved the preliminary description of DLTS to the SI (Supplementary Note 3).

Reviewer 4:

This research article by Zhou et al. reports a study of modified chemical bath deposition (CBD) of Sb_2S_3, resulting in solar cells with an open-circuit voltage (V_{OC}) exceeding 800 mV. The authors introduce the sodium citrate (SC) complex agent to regulate the CBD of Sb_2S_3, thereby reducing grain boundary (GB) density and suppressing mid-gap defect states. As a result, they achieved Sb_2S_3 solar cells with a record high V_{OC} of 824 mV. The authors further investigate defects by combining deep-level transient spectroscopy (DLTS) measurements with density-functional theory (DFT) defect calculations, providing a comprehensive understanding of defect properties in Sb_2S_3. Overall, this is a well-organized study with relatively high significance and novelty. I would consider recommending this manuscript for publication if the remaining issues are addressed. Detailed comments are provided below.

Response: We thank the reviewer for their time in carefully evaluating our paper, and we are glad to see that they recognize the valuable insights into the defect properties and device performance of Sb_2S_3 solar cells in this work.

[1] In the introduction, the authors claim, 'At present, the V_{OC} of Sb_2S_3 solar cells has stagnated in the range of 550-770 mV[SZ1] for decades.' However, in Fig. 2e and the corresponding Supplementary table, at least two papers report V_{OC} values of ~800 and 815 mV. The authors should correct this statement to be accurate about the establishment in the field.

Response: We thank the reviewer for the valuable comment. As shown in Fig. 2e and Supplementary Table 1, which summarize the performance parameters of previously-reported

Sb_2S_3 solar cells with competitive PCEs, the highest V_{OC} for Sb_2S_3 solar cells recently reached 811.5 mV (*Mater. Today Energy* 2025, 54, 102083). Based on the reviewer's suggestion, we have made corresponding revisions for this statement (Page 4, highlighted in blue):

"At present, the highest V_{OC} reported so far for Sb_2S_3 solar cells is 811.5 mV, and was achieved through in-situ passivation of hydrothermal-processed absorber films[26]; however, most Sb_2S_3 devices still show V_{OC}s in the range of 550–770 mV, much smaller than other photovoltaic devices based on absorbers with similar or smaller bandgaps, such as CdTe, GaAs, CIGS, InP, $CH_3NH_3PbI_3$ or crystalline-Si, as summarized in Fig. 1a[1, 27]"

[2] Could the authors provide references to support the statement, 'This mechanism is linked to their experimental observation of a 600 meV red-shift in photoluminescence (PL) compared to the optical bandgap, along with polarized light emission from Sb_2S_3 single crystals?' The PL redshift could also be related to the emission from defect/impurity levels. Why is it claimed as evidence for self-trapped exciton states, which typically are not as deep as 600 meV?

Response: We thank the reviewer for raising this point.

(1) This statement is obtained from the work of Yang *et al.*, as described in the Introduction (*Nat. Commun.* 2019, 10, 4540; ref. 29 in our paper). They performed PL studies on Sb_2S_3 polycrystalline films and single crystals, revealing PL that is 0.6 eV red-shifted compared to the optical bandgap. Furthermore, they show that the emission from this red-shifted state is strongly polarized along the c-axis. Hence, they claimed that this polarized trap emission is unlikely to arise from surface defects states or impurities, which usually would be randomly distributed and lead to isotropic trap emission.

(2) Based on the optical properties and photoexcitation dynamics observed, including the strongly red-shifted trap emission, picosecond carrier trapping dynamics, trapped carrier induced absorption without saturation at 10^{20} cm^{-3} carrier density, and polarized trap emission in single crystals, Yang *et al.* suggested that photoexcited carriers in Sb_2S_3 are intrinsically self-trapped by lattice deformation, instead of by extrinsic defects. Self-trapping through small polaron formation could occur even in a perfect crystal and create a transient defect state in bandgap by lattice deformation, leading to substantial energy loss and a Stokes-shifted PL.

(3) In the revised manuscript, we have modified the statements to make the expression clearer, with reference added (Page 4, highlighted in blue):

"This mechanism is linked to the experimental observation of linearly polarized photoluminescence (PL) that was 600 meV red-shifted compared to the optical bandgap of Sb_2S_3[29]. These features were attributed to emission from STE states, and from this data, it was hypothesized that the maximum V_{OC} would be 600 mV below the radiative limit of 1.4 V for a 1.7 eV bandgap semiconductor; that is, the maximum V_{OC} would be only 800 mV for Sb_2S_3 if this hypothesis were true[29]".

[3] In the statement, 'Fig. 3a and b shows top-view scanning electron microscopy (SEM) images of Sb_2S_3 thin films prepared without the SC additive (i.e., the control sample) and with different concentrations of 4 mM SC,' the words "with different concentrations of 4 mM SC" are typos since the concentration is at 4 mM.

Response: We thank the reviewer for the reminder. We have made corresponding revisions in the revised version (Page 9, highlighted in blue):

"Fig. 3a and b show top-view scanning electron microscopy (SEM) images of Sb_2S_3 thin films prepared without the SC additive (i.e., the control sample) and with 4mM SC in the precursor solution, respectively.".

[4] There are typos in the figure numbers.

In 'Fig. 3a gives the dependence of GB density on the SC concentration for different Sb_2S_3 films,' it should be Fig. 3c rather than 3a.

In 'Fig. 3c shows the high-resolution TEM (HRTEM) images of three arbitrarily selected points A1, A2, and A3, as labeled in Fig. 3b.' The correct figure numbers should be Fig. 3e and Fig. 3d.

Response: We greatly appreciate the reviewer for spotting these typos, which we have now corrected. We have checked through the main text again to identify and correct any other typos.

[5] In the authors' hypothesis of SC's working mechanism, additional evidence is needed to determine whether pH affects grain size and GB density. Though pH does not significantly affect device performance, it may change the nucleation and growth. If this is true, then the passivation effect of SC complexation to S vacancies and Sb antisites could be the driving force for the enhanced V_{OC}.

Response: We thank the reviewer for raising this point.

(1) In our recently-published work (*Small Methods* 2026, 10, e02005), we carefully studied the influence of precursor pH on film properties and device performance. These prior results show that pH does not exert great impacts on the grain size and GB density based on SEM images of Sb_2S_3 films obtained under different precursor pH (Figure 2 and Figure S2 in the paper *Small Methods* 2026, 10, e02005), and the PCE change should be mainly associated with the variation in the film thickness of Sb_2S_3.

(2) Here in this work, aiming to decouple the impacts of precursor pH and SC additive on the film properties and device performance, we also studied the influence of pH on the deposition of Sb_2S_3 films, as detailed in Supplementary Note 2 and Supplementary Fig. 16. The results show that, besides the complexation of Sb^{3+} with citrate, the increase in pH also contributes to a reduction in the deposition rate. However, controlling pH alone does not lead to increases in PCE as much as the addition of SC. It was revealed that the incorporation of ammonia solution does not result in remarkable efficiency increase although this precursor shares a similar pH value as the precursor with the addition of 4 mM SC. The results imply that the increase in PCE for SC-Sb_2S_3 solar cells are mainly associated with the complexation Sb^{3+} with citrate, which causes the evolution in the morphology of Sb_2S_3 absorber films. Defect characterization and computations reveal that the reduced defect activity in large-grained Sb_2S_3 films are correlated with the increase in device V_{OC}.

[6] Parameters and variables in Eq. 2 need to be further explained.

Response: According to the reviewer's suggestion, we have made further clarification for the parameters and variables in Eq. 2 (original version). In the revised manuscript, we have moved this preliminary description of DLTS to the SI (Page S6-S7, Supplementary Note 3).

“where t_1 and t_2 determine a fixed time window used to calculate $\Delta C = C_{t2} - C_{t1}$ (Fig. 4a). In this work, the time window was set to be 100 ms”.

[7] For Eqs. 3 and 4, could emission time be different for electrons and holes?

Response: Thanks for raising this point. The electron emission time constant, τ_e, is determined by a fixed time window between t_1 and t_2, expressed by the Eq. S14:

$$\tau_e = \frac{t_2 - t_1}{\ln(\frac{t_2}{\tau_1})} \qquad \text{(S14)}$$

For the analysis of a specific case, the hole emission time constant τ_p is typically based on an identical fixed time window, i.e., $\tau_p = \tau_e$.

[8] Since C-V measurements were performed, the depletion width and apparent carrier density should be estimated and discussed.

Response: We thank the reviewer for this suggestion. According to the comments from Reviewer 3, we realized that the C-V analysis might not be valid for Sb_2S_3 due to the low carrier concentrations in the films. In order to avoid misleading readers, we have deleted the C-V characterization and corresponding analysis. This does not influence the novelty of this work and the discussion of results.

Reviewer comments are copied out in black. Our responses are in blue.

Reviewer 1:

I thank for the additional work on the manuscript. After presenting the temperature-dependent mobility data, the measurements on thickness-comparable samples, and authors' clarification on the theory and the novelty and importance of the data, the manuscript is ready for publishing as it is with no further comments from my side. All the best!

We would like to thank the reviewer for her careful and thorough evaluation of our paper, and are delighted to see her accept our paper.

Reviewer 2:

The Reviewer's main concern is still only partially resolved. The manuscript does not quantitatively connect the proposed defect-based picture to the previously reported ~0.6 eV Stokes-shifted emission that motivated the self-trapping hypothesis. The response only states that the defect relaxations are "qualitatively consistent," but no explicit calculated relaxation energies or direct comparison to the ~0.6 eV shift are provided. This remains insufficient.

Response: We thank the reviewer for raising this point, and appreciate that it would be satisfying if we were able to provide our explanation for the 0.6 eV red-shift in PL that was previously reported. However, such a change to our paper would only be appropriate if the red-shift were universally observed in Sb_2S_3, and if it were unambiguously a property multiple groups consistently found to occur for this material. The complicating factor is that PL in Sb_2S_3 is typically weak at room temperature, and therefore care needs to be taken that the PL measured originates from the material itself and not any other sources.

We therefore carefully searched through the literature to obtain reliable reports of PL from Sb_2S_3, prepared by a variety of processing methods. These are summarised in Supplementary Table 2 in the revised version, copied below for convenience. As shown through this literature analysis, only Yang *et al.* (Ref. 1 in the table) reported a red-shift in PL as large as 0.5-0.6 eV. In nearly all other reports, the red-shift was only 0-0.1 eV. Moreover, the fact that there is some variation in the PL energy is consistent with this being strongly affected by defects; if it were due to self-trapping, the PL energy should have been more consistent if it were an intrinsic materials property.

We cannot know the exact conditions that may have caused this unusual red-shift reported by Yang *et al.* For example, given the weak PL from Sb_2S_3, extrinsic impurities within the bulk or surface could have been a factor. But without the exact samples at hand, we cannot know for certain. It is therefore inappropriate for us to speculate on this.

That being said, this analysis of literature reports of PL is a useful addition to our paper. This is because part of the justification for self-trapping and V_{OC} loss hypothesised by Yang *et al.* was based on the observed large red-shift in PL. Showing that this is rarely observed in literature provides further motivation to re-examine this hypothesis in detail, which we do so in this work to provide experimental evidence disproving this hypothesis. We have added the following to the introduction of our revised paper (Page 5):

"Moreover, the justification for self-trapping and V_{OC} loss hypothesised by Yang et al. was based on the observed large red-shift in PL compared to the optical bandgap; however, through a careful literature analysis based on reliable reports of PL from Sb_2S_3 prepared by a variety of processing methods, the red-shift of PL for Sb_2S_3 was found to be only 0-0.1 eV in nearly all other reports (Supplementary Table 2). This further motivates the re-examination of this self-trapping hypothesis in detail."

Supplementary Table 2. Summary of PL characteristics of kinds of Sb_2S_3 samples obtained from different methods in the literature.

No.	Samples	Deposition methods	PL peak (eV)	E_g (eV)	Red shift (eV)	Excitation	Ref.
1	FTO/CdS/Sb_2S_3 films	HT[a]	1.15	1.65	~0.5	CW 532 nm laser	1
	Single-crystal flake	CVT[b]	1.1	1.7	~0.6	CW 532 nm laser	
2	glass/Sb_2S_3 films	SC[c]	~ 1.71, Symmetrical	1.73	~ 0.02	CW 405 nm laser	2
3	FTO/CdS/Sb_2S_3 films	RTE[d]	~ 1.63, Symmetrical	1.64	~ 0.01	532 nm pumped laser	3
4	FTO/CdS/Sb_2S_3 films	HT[a]	~ 1.63, Symmetrical	1.69	~ 0.06	532 nm laser	4
5	glass/FTO/CdS/ Sb_2S_3 films	CSS	1.72, unsymmetrical	1.8	0.08	266 nm laser	5
6	FTO/CdS/Sb_2S_3 films	HT[a]	~ 1.64, Symmetrical	1.77	~ 0.13	532 nm laser	6
7	FTO/TiO_2/CdS/ Sb_2S_3 films	SC[c]	~ 1.71, Unsymmetrical	1.87	~ 0.16	unmentioned	7
8	FTO/SnO_2/CdS/ Sb_2S_3 films	HT[a]	1.64, Symmetrical	1.63	~ 0	532 nm laser	8

9	FTO/CdS/Sb_2S_3 films	HT[a]	~ 1.70, Symmetrical	1.72	~ 0.02	unmentioned	9
10	FTO/TiO_2/Sb_2S_3 films	CBD[e]	~ 1.69, Unsymmetrical	1.86	~ 0.17	unmentioned	10
11	SLG/Mo/Sb_2S_3 films	VTD[f]	1.64, Symmetrical	1.72	0.08	unmentioned	11
12	glass/ITO/Sb_2S_3 films	Sol-gel	~ 1.68, Symmetrical	1.68	~ 0	638 nm laser	12
13	Glass/Mo/Sb_2S_3 films	Sputtering	1.664, Symmetrical	1.778	0.114	532 nm laser	13
14	ITO/Sb_2S_3 films	SC[c]	1.78, Symmetrical	1.78	0	266 nm laser	14
15	Mo/Sb_2S_3 films	EBE[g]	~ 1.44 eV Unsymmetrical	1.70	~ 0.26	510 nm laser, 50 K	15
16	Au-coated ITO/ Sb_2S_3 films	TE[g]	~ 1.73 eV Symmetrical Weak	1.76	~ 0.03	unmentioned	16
17	quartz/Sb_2S_3 films	SC[c]	1.682 eV Symmetrical Weak	1.71	0.028	510 nm pulsed laser	17
18	Sb_2S_3 nanoparticles (~10 nm)	Hot-injection	1.88, Unsymmetrical	1.93	0.05	633 nm laser	18
19	Si/SiO_2/ single-crystalline Sb_2S_3 rods	HT[a]	1.67, Symmetrical	1.61	~ 0	532 nm laser	19
20	FTO/Sb_2S_3 nanorod arrays	SC[c]	1.65, Symmetrical	1.65	0	371 nm laser	20

Note

[a] HT: hydrothermal deposition;

[b] CVT: Chemical vapor transport;

[c] SC: Spin-coating;

[d] RTE: rapid thermal evaporation;

[e] CBD: Chemical bath deposition;

[f] VTD: vapor transport deposition;

[g] EBE: Electron-beam evaporation

[h] TE: Thermal evaporation

Furthermore, we checked the PL from our Sb_2S_3 films deposited by chemical bath deposition. Consistent with prior reports, the PL was weak at room temperature, and we were not able to measure PL using conventional spectroscopy setups. We were only able to observe PL using a Raman microscopy setup, where the excitation laser source was concentrated over a small spot to give high fluence. The red-shift in PL compared to the optical bandgap is approximately 0.1 eV, consistent with the majority of reports shown in Supplementary Table 2.

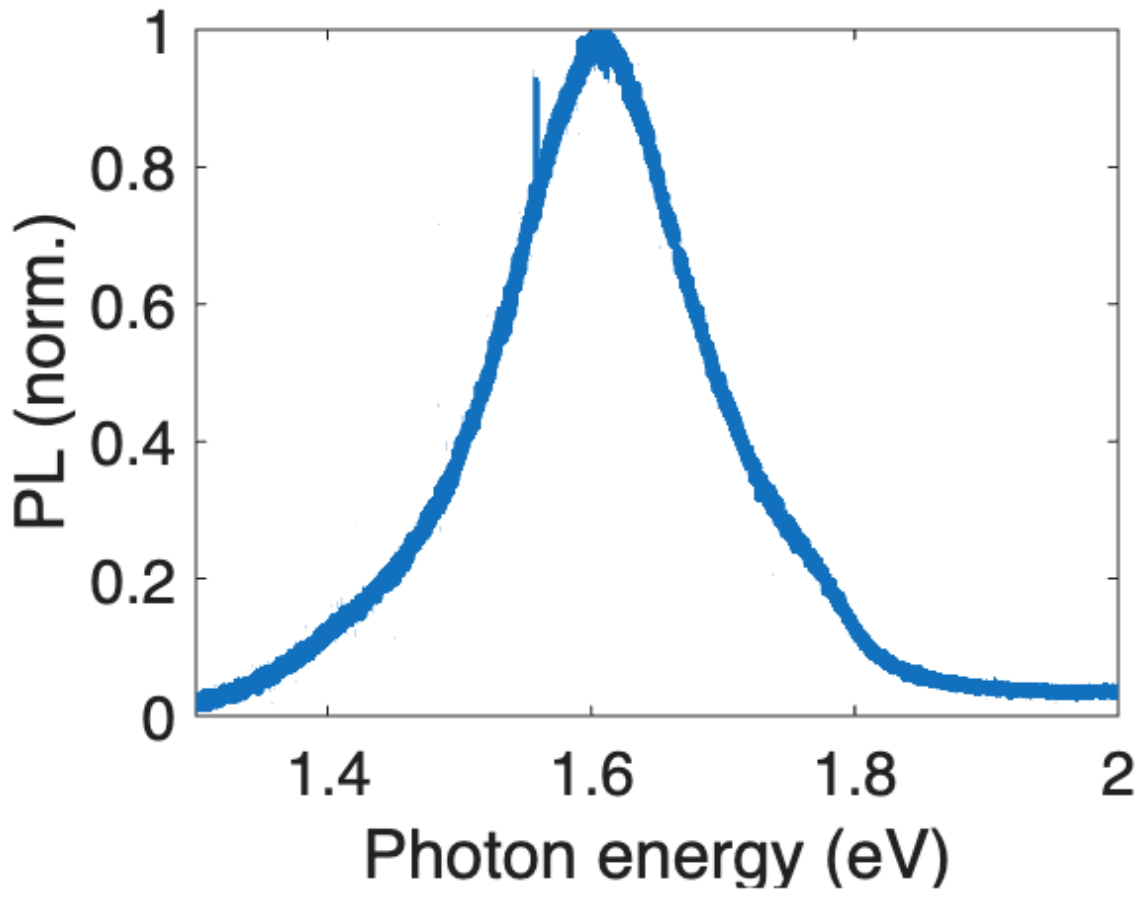

Figure R1. PL measurements of Sb_2S_3 thin films deposited by chemical bath deposition. Measurement collected by Xiaoyu Guo in the group of Prof. Robert Hoye using a Renishaw setup with an excitation wavelength of 532 nm and 5% power. The main peak is at 1.61 eV, with a shoulder centred at approximately 1.74 eV. The latter is coincident with the bandgap we determined from Tauc plots (1.72-1.75 eV; refer to Supplementary Information, page S15). The PL red-shift is therefore approximately 0.1 eV, consistent with the majority of prior reports shown in Supplementary Table 2, and well below the 0.6 eV value reported by Yang *et al.*

I also do not find the argument based on temperature-induced band-edge shifts convincing in its present form. The cited ~0.2 eV value from Liu et al. (2023) refers to the temperature dependence of the bulk Sb2S3 bandgap, not to the temperature dependence of defect transition levels. The manuscript effectively extrapolates from a bulk band-edge shift to a defect-level shift of similar magnitude without demonstrating that this connection holds for the specific defects under discussion. These are not generally equivalent quantities. The effect of electron-phonon coupling on the band edges is not the same as its effect on deep defect levels, so a similar numerical scale does not by itself establish a physical explanation for the 0.1-0.2 eV discrepancy.

Response: We thank the reviewer for this important comment. We agree that the temperature dependence of the bulk bandgap should not be used as a quantitative correction for defect transition levels. We have therefore removed this argument.

Instead, we now clarify that the difference between calculated 0 K thermodynamic transition levels and finite-temperature DLTS activation energies is within the typical uncertainty of state-of-the-art hybrid-functional defect calculations, which is on the order of 0.1 eV (Turiansky M E, et al. MRS Bulletin, 2026: 1-10; Chen W, Pasquarello A. Physical Review B, 2017, 96(2): 020101). Specifically, the calculated $V_{S(3)}$ and $Sb_{S(2)}$ transition levels at 0.59 and 0.61 eV above

the VBM are closest to the experimentally observed H2 level, differing by only 0.05 eV and 0.03 eV, respectively. Their differences from H1 are larger but still on the order of 0.1 eV. This level of agreement supports assigning these defects as deep hole traps, especially when combined with their calculated concentrations and hole capture cross sections.

We have clarified this in the main text (Page 19, highlighted in blue):

"*Based on the combined information from defect levels, concentrations, and capture cross sections, we suggest* $V_{S(3)}$ *and* $Sb_{S(2)}$ *as the defects that are most consistent with the experimental observations, with calculated trap levels located at 0.59 and 0.61 eV above the VBM and calculated capture cross sections of* 3.77×10^{-16} *and* 4.00×10^{-17} cm^{-2}*, respectively. These calculated levels are closest to the experimentally observed H2 levels, differing by approximately 0.05 eV for* $V_{S(3)}$ *and 0.03 eV for* $Sb_{S(2)}$*. Their differences from the H1 levels are larger but remain on the order of 0.1 eV. We note that the calculated transition levels are the thermodynamically stable states at absolute zero temperature, whereas DLTS extracts activation energies from finite temperature carrier emission processes. The small differences between the calculated and experimental trap levels on the order of 0.1 eV or less are consistent with the typical uncertainty in comparing state-of-the-art hybrid functional defect calculations with experimental defect levels. We do, however, acknowledge, that these deviations prevent us from unambiguously assigning the experimental H1 and H2 levels to* $V_{S(3)}$ *and* $Sb_{S(2)}$*. Regardless, this does not affect the conclusion that* $V_{S(3)}$ *and* $Sb_{S(2)}$ *are deep hole traps with efficient nonradiative capture.*"

More importantly, this temperature-effect argument does not actually resolve Reviewer's central spectroscopic point. Reviewer asked whether the calculated defect relaxation energies (Franck-Condon shifts) can explain the previously reported ~0.6 eV Stokes shift. Instead, the response discusses possible temperature-induced shifts in defect level positions. These are different physical quantities. The former concerns relaxation energy associated with optical emission, whereas the latter concerns the absolute energetic position of defect levels. As written, the manuscript substitutes an argument about level shifts for a question about relaxation energies, and therefore does not directly address the key issue.

Response: We believe we have now addressed this question with our answer to the first question. To summarise – we conducted careful analysis of the literature to find that the reported 0.6 eV red-shift in PL to be unusual among reports for Sb_2S_3. We therefore cannot

conclude that this large red-shift is universal in Sb_2S_3, and may, for example, be caused by undetected extrinsic impurities in the particular samples studied, or could be affected by measurement artefacts, owing to the low intensity of the PL from Sb_2S_3. Without the exact samples at hand, it is inappropriate for us to speculate in the paper. But we can say that the lack of universality of the reported 0.6 eV red-shift in PL raises further questions of the self-trapping hypothesis, providing strong motivation for our work re-examining this hypothesis.

Reviewer 3:

The authors have provided reasonable evidence of large polarons rather than small polarons in their Sb2S3. They have also reconsidered claims that were not fully backed by experimental evidence, making the paper more convincing without loss of impact. Most of their responses and changes are well reasoned and appreciated.

We would like to thank the reviewer for their time and careful evaluation of our work. We are delighted to see that they judge our work to be strengthened and more convincing without loss of impact. We have now addressed the comments raised, as detailed below.

There are four aspects that are still not convincing:

1. The title is “Exceeding the 800 mV open-circuit voltage threshold in antimony sulfide photovoltaics “, but it appears that there is a published report of 811 mV Voc for Sb2S3 solar cells (I was not aware of it in the first review round). Thus, the title is not quite truthful and should be changed, so that it is not implied that this is the first study to surpass the 800 mV threshold, which is not true. Still, this study reports the highest Voc to date, so it should not be a problem to highlight the achievement.

Response: We agree with the reviewer, and have now changed the title to “*Antimony sulfide photovoltaics with high open-circuit voltage not limited by self-trapped excitons*”. This captures the core novelty of the paper in disproving the self-trapping hypothesis, showing the potential of Sb_2S_3 is not limited as previously proposed. The title also captures the fact that we achieved a record value for the open-circuit voltage (without using the word “record” in the title), and does not imply we are the first to exceed a 800 mV open-circuit voltage threshold.

2. The relationship between efficiency and grain boundary density is not convincing. There is no evidence of causation, only correlation. The authors argue that "the overall reduction in trap state density that we measured is correlated with the reduction in GB density [correct]. It is reasonable to deduce that the recombination losses in Sb2S3 are closely associated with GBs [incorrect], where dangling or wrong bonds proliferate, including the GBs located at the film interface and film interior [The film surface is a giant grain boundary even for a single-crystalline film, so at the surface it doesn't matter what the grain size is. In fact, surface passivation is essential even in single-crystalline PV technology such as c-Si or III-V. At the film interior it is highly questionable whether GBs matter with grains of this size. Yes, they could, but there is no evidence to support this.]. The authors also state "the Sb2S3-based devices would also inevitably [incorrect] involve the negative effects of GBs with dangling bonds at the surface and interior of polycrystalline films, which would be detrimental to device performance [not necessarily correct]." Finally, the authors state "from the c-AFM measurements, it can be seen that the GBs generally have lower conductivity than the grain interiors, suggesting inefficient charge-carrier separation [why would lower conductivity in the dark imply inefficient carrier separation? I do not see the connection] and transport [possibly, but only if the conductivity decrease is due to a mobility decrease. If it's due to a decrease in dark carrier concentration, carrier transport under illumination is more likely to be unaffected] as a result of defects at the GBs39. None of these statements makes a convincing case. Insisting on this connection really has no impact on the overall quality of the paper. What if recombination at GBs is not the reason for improved performance, but instead an improved growth process results in lower bulk defect density, which in turn causes the grains to grow bigger? This hypothesis is entirely plausible, and it would imply that there is no cause-effect relationship between GB density and Voc – only a correlation.

Thus, I think the revised text "It is generally accepted that, in polycrystalline films, dangling or wrong bonds proliferate at GBs, which would result in increased non-radiative recombination losses and be detrimental for device performance. Hence, increased device performance with polycrystalline absorbers would often be correlated with reduced GB density" is still too speculative and should be removed.

Response: We thank the reviewer for these valuable comments, and for the continued support with suggestions to help us provide clearer expressions on the relationship between GB density

and device performance. According to the reviewer's suggestions, we have removed these problematic statements and made corresponding revisions.

(1) The as-mentioned statement "*It is generally accepted that, in polycrystalline films, dangling or wrong bonds proliferate at GBs, which would result in increased non-radiative recombination losses and be detrimental for device performance. Hence, increased device performance with polycrystalline absorbers would often be correlated with reduced GB density*" has been removed in the revised version.

(2) For the analysis of c-AFM measurements, we have also removed corresponding descriptions, i.e., we have removed the sentence sentence "*Moreover, from the c-AFM measurements, it can be seen that the GBs generally have lower conductivity than the grain interiors, suggesting inefficient charge-carrier separation and transport as a result of defects at the GBs*".

In the revised manuscript, we have retained the following statement: "*Conductive-atomic force microscopy (c-AFM) images, with the corresponding morphology and current maps (Supplementary Fig. 11) reveal that, compared to the control sample, the 4mM-SC films have more uniform electrical conductivity over the 5×5 μm^2 area measured.*"

(3) When discussing the overall reduced trap density from SCLC results, we have added one more sentence to clarify the presentation of different hypotheses for the reduced trap density that is measured (Page 23, highlighted in blue):

"*Here it is worth noting that the reduced trap density in absorber films could arise from lower GB density caused by the incorporation of SC additives, or could be due to lower bulk trap density as a result of improved growth conditions. Future detailed investigations into the role of GB, surface and bulk traps on non-radiative recombination are required to identify the main bottlenecks on performance now.*"

3. Some of the revised text reads. "The reduced surface roughness of the absorber films would favor the formation of an improved heterojunction between Sb2S3 and solution-processed HTL on top". Why should this be the case? This seems to be speculative, and there are no references to support this claim. Si solar cells are intentionally roughened to enhance light scattering, and this can still give > 27% efficient cells.

Response: We thank the reviewer for raising out this. In the revised manuscript, we have deleted the sentence mentioned above, and this would not impact the discussion of results and the novelty of this work.

4. About the 1.70 V open-circuit voltage limit. I understand that using a non-stepwise absorption profile can lead to a different, possibly higher Voc limit than the SQ prediction. However: (i) Figure 1(a) makes it quite confusing for the reader to see the Voc of many PV materials lined up against their band gap and their SQ Voc limit, to later be told that in reality the real radiative Voc limit is much higher than what Figure 1(a) says. (ii) Radiative Voc limits that are based on other formalisms than the SQ one are not as established, so they require a higher standard of proof. I do not think it's sufficient for the authors to claim that they used package XYZ and obtained a Voc limit of XYZ. The authors should explicitly show the equations and assumptions used to derive this limit.

Response: We thank the reviewer for these helpful comments. In the revised manuscript, we now clarify that Fig. 1a uses the ideal Shockley-Queisser limit with a step-function absorptance at the bandgap. This is used only as a common reference for comparing different photovoltaic materials.

By contrast, the 1.70 V value used in our trap-limited calculation is a material-specific radiative V_{OC}, calculated using the finite thickness absorptivity of Sb_2S_3, which provides a more realistic reference for the actual Sb_2S_3 absorber. The underlying formalism is still based on the standard SQ detailed balance framework. The only modification is replacing the ideal step function absorptivity with the finite thickness absorptivity calculated from the real Sb_2S_3 absorption profile.

We have revised the wording in the manuscript and figure caption to make clear that these two quantities are calculated in different ways.

- On page 3-4: "*Here, it is worth noting that this standard detailed balance formalism assumes a step-function absorptance at the bandgap and radiative recombination only*" (Shockley, W.; Queisser, H. J. *Journal of Applied Physics* **1961**, *32*, 510-519; Rühle, S. *Solar Energy* **2016**, *130*, 139-147).
- Caption of Figure 1(a): "…*Theoretical S-Q limit of V_{OC} (calculated based on step-function absorptance)*…"

- On page 4: "*To date, the V_{OC} of 753 mV obtained in the highest-efficiency (i.e., 8.32% PCE) Sb_2S_3 device is only 53.7% of the conventional S-Q V_{OC} limit* (Shockley, W.; Queisser, H. J. *Journal of Applied Physics* **1961**, *32*, 510-519)."
- On page 20: "*It is worth noting that, within the conventional S-Q detailed balance framework, a material-specific radiative reference based on the finite-thickness absorptance of Sb_2S_3 is used for the trap-limited V_{OC} analysis*".
- Supplementary Note 4: "*Within the conventional S-Q detailed balance framework, we replace the ideal step-function absorptance with the finite-thickness absorptance of Sb_2S_3. A representative film thickness of 350 nm was used to ensure a consistent radiative limit for both samples. Using this finite-thickness absorptance, we calculated the radiative V_{OC} limit to be 1.70 V*".

We have also expanded the Methods section to explicitly show the equations and assumptions used in the calculation (Page 30-31, highlighted in blue):

"The maximum power conversion efficiency (η_{max}) of a solar cell is expressed as:

$$\eta_{max} = max_V\left(\frac{JV}{e\int_0^{\infty} E\phi_{sun}(E)dE}\right) \quad (7)$$

where e is the elementary charge. $\phi_{sun}(E)$ is incident spectral photon flux density at the photon energy E. A standard AM1.5 solar spectrum is used in this work.

Following the methodology by Kim et al.[21, 22], the net current density $J(V;W)$, accounting for both radiative and non-radiative recombination, is given by:

$$J(V;W) = J_{SC}(W) + J_0^{rad}(W)\left(1 - e^{\frac{eV}{k_BT}}\right) - eR(V)W \quad (8)$$

where $R(V)$ is the defect-mediated non-radiative recombination rate. W is the film thickness. J_0^{rad} is the saturation current.

Assuming that each absorbed photon contributes one collected electron hole pair, the short-circuit current J_{SC} is given by

$$J_{SC}(W) = e\int_{E_g}^{\infty} a(E;W)\phi_{sun}(E)dE \quad (9)$$

The radiative limit is obtained by setting the defect mediated nonradiative recombination term R(V) to zero and solving $J\left(V_{OC}^{rad};W\right)=0$, giving

$$V_{OC}^{rad}(W) = \frac{k_BT}{e}ln\left(\frac{J_{SC}(W)}{J_0^{rad}(W)} + 1\right) \quad (10)$$

The radiative saturation current is calculated from detailed balance as

$$J_0^{rad}(W) = e\int_0^{\infty} a(E;W)\,\phi_{bb}(E)dE \quad (11)$$

where $\phi_{bb}(E)$ is the black body photon flux. The photon absorptivity $a(E;W)$ describes the probability of absorption as a function of photon energy. In the Shockley-Queisser model, it is idealised as a step-like function at the bandgap. In our calculation, the absorptivity is defined as $a(E;W) = 1 - e^{-2\alpha(E)W}$, where $\alpha(E)$ is the optical absorption coefficient of Sb_2S_3 obtained from first-principles calculations using the HSE06 functional[23]. The factor of 2 accounts for a double-pass optical path through the absorber, assuming perfect reflection from the back contact. A film thickness of W=350 nm was used to match the experimental absorber thickness."

Reviewer 4:

The authors have adequately addressed the previous round of comments from reviewers. The newly added temperature-dependent mobility measurements support their conclusions.

The reviewer agrees that this work is novel in its experimental demonstration that the performance of Sb2S3 photovoltaics is limited by defects rather than by self-trapping, disproving a previous hypothesis published in the same journal. The study is sound and supported by experimental evidence and theory.

Therefore, the reviewer recommends this study for publication as is.

Response: We are delighted by the positive appraisal by the reviewer for our revised work, and that they have accepted our paper. We thank them for their time.

References

1. Yang, Z. L. *et al.* Ultrafast self-trapping of photoexcited carriers sets the upper limit on antimony trisulfide photovoltaic devices. *Nat. Commun.* **10**, 4540 (2019).

2. Jia, Z. L. *et al.* Charge-carrier dynamics of solution-processed antimony- and bismuth-based chalcogenide thin films. *ACS Energy Lett.* **8**, 1485-1492 (2023).

3. Zeng, Y. Y. *et al.* Quasi-vertically-orientated antimony sulfide inorganic thin-film solar cells achieved by vapor transport deposition. *ACS Appl. Mater. Interfaces* **12**, 22825-22834 (2020).

4. Mao, Y. *et al.* Molten salts assisted interfacial engineering for efficient and low-cost full-inorganic antimony sulfide solar cells. *Adv. Funct. Mater.* **32**, 2208409 (2022).

5. Krautmann, R. *et al.* Low processing temperatures explored in Sb_2S_3 solar cells by close-spaced sublimation and analysis of bulk and interface related defects. *Sol. Energ. Mater. Sol. C.* **251**, 112139 (2023).

6. Lin, L. M. *et al.* Ambient confined-space annealing for crystallization enhancement and defect passivation in Sb_2S_3 thin-film solar cells. *Nano-Micro Lett.* **18**, 359 (2026).

7. Tao, Y. F. *et al.* Cs doping engineering effectively improves photovoltaic performance of Sb_2S_3 solar cells. *J. Phys. Chem. C.* **129**, 17946-17954 (2025).

8. Yu, Z. *et al.* Copper-based semiconductor nanocrystal hole-transport layers enable efficient and stable Sb_2S_3 solar cells. *ACS Appl. Mater. Interfaces* **17**, 42118-42127 (2025).

9. Yu, F. C. *et al.* In-situ sulfur passivation enabling high open-circuit voltage in hydrothermally Sb_2S_3 solar cells. *Mater. Today Energy* **54**, 102083 (2025).

10. Tao, Y. *et al.* Cd source optimization enhances solution-processed Sb_2S_3 solar cell efficiency. J. Alloy. Compd. **1039**, 182943 (2025).

11. Sharma, I. *et al.* Vapor-transport-deposited Sb_2S_3 thin-film solar cells: Tailoring photovoltaic properties through deposition temperature. *J. Power Source. Adv.* **26**, 100143 (2024).

12. Li, Y. *et al.* Precursor engineering of solution-processed Sb_2S_3 solar cells. *Small* **20**, 2308895 (2024).

13. Wang, Z. *et al.* Self-assembled growth of quasi-vertically oriented Sb_2S_3 thin films for substrate structured solar cells. *Ceram. Int.* **50**, 4729-4736 (2024).

14. Chihi, A. Gamma-irradiated stibnite thin films set a remarkable benchmark performance for photoelectrochemical water splitting. *RSC Adv.* **14**, 12475-12495 (2024).

15. Yang, M. J. *et al.* Tailoring the crystallographic orientation of a Sb_2S_3 thin film for efficient photoelectrochemical water reduction. *ACS Catal.* **12**, 8175-8184 (2022).

16. Wang, L. *et al.* A transparent, high-performance, and stable Sb_2S_3 photoanode enabled by heterojunction engineering with conjugated polycarbazole frameworks for unbiased photoelectrochemical overall water splitting devices. *Adv. Mater.* **34**, 2200723 (2022).

17. Han, J. *et al.* Multidentate anchoring through additive engineering for highly efficient Sb_2S_3 planar thin film solar cells. *J. Mater. Sci. Technol.* **89**, 36-44 (2021).

18. Ilic, N., Zatsepin, D. A., Biryukov, D. Y., Zatsepin, A. F., Validzic, I. Temperature-dependent photoluminescence of the synthesized Sb_2S_3 particles: From nanoparticles to crystalline samples. *Opt. Mater.* **162**, 116803 (2025).

19. Liu, X. H. *et al.* Improving the optoelectronic properties of single-crystalline antimony sulfide rods through simultaneous defect suppression and surface cleaning. *J. Mater. Chem. A* **11**, 8826-8835 (2023).

20. Park, Y. S. *et al.* Surface-passivated vertically oriented Sb_2S_3 nanorods photoanode enabling efficient unbiased solar fuel production. *Adv. Energy Mater.* **13**, 2301166 (2023).

21. Kim, S., Márquez, J. A., Unold, T. & Walsh, A. Upper limit to the photovoltaic efficiency of imperfect crystals from first principles. *Energ. Environ. Sci.* **13**, 1481-1491 (2020).

22. Kim, S. & Walsh, A. Ab initio calculation of the detailed balance limit to the photovoltaic efficiency of single p-n junction kesterite solar cells. *Appl. Phys. Lett.* **118**, 243905 (2021).

23. Wang, X. W., Li, Z. Z., Kavanagh, S. R., Ganose, A. M. & Walsh, A. Lone pair driven anisotropy in antimony chalcogenide semiconductors. *Phys. Chem. Chem. Phys.* **25**, 25055-25055 (2023).

Reviewer comments copied out in black, our response in blue.

Response to reviewer comments

Reviewer 2:

I am satisfied that the temperature-related concern has been adequately addressed.

My principal concern, however, remains unresolved, because the authors' response addresses a different question from the one I raised. I asked for a quantity intrinsic to the authors' own model: the calculated Franck–Condon relaxation energies, and the predicted Stokes shifts, of the proposed dominant defects V_S(3) and Sb_S(2). The authors instead argue that the previously reported ~0.6 eV red-shift is atypical in the literature and may be an artefact.

These are logically independent. Even if Yang et al.'s value were entirely artefactual, the Stokes shift predicted by the authors' own defect model remains a well-defined quantity that has not been reported, and its evaluation does not depend on that measurement at all. An argument about non-universality therefore cannot substitute for the calculation, and the reported transition levels and capture cross sections are not the relaxation energy in question.

The authors need not explain the ~0.6 eV shift, but they must close the loop on their own mechanism: for at least V_S(3) and Sb_S(2), they should report the configuration-coordinate diagrams and calculated relaxation energies—which, if the capture cross sections were obtained from non-radiative multiphonon theory, are already available—and state the Stokes shift their model predicts, comparing it with both the ~0.6 eV value and the ~0.1 eV shift measured here. A quantitative prediction, whatever its magnitude, would resolve this; reaffirming that the ~0.6 eV shift is atypical would not. This need to be addressed.

Response: We thank the reviewer for clarifying the request. We agree that the Franck-Condon relaxation energies and Stokes shifts predicted by our defect model are well defined quantities, and we have now calculated and reported these quantities explicitly for $V_{S(3)}$ and $Sb_{S(2)}$.

- Franck-Condon relaxation energies and defect transition Stokes shifts

From the configuration coordinate diagrams in Fig. 5d and e, we evaluated the relaxation energies of the initial and final states, λ_i and λ_f, and the defect Stokes shift, $\Delta E_{Stokes} = E_{abs} - E_{em} = \lambda_i + \lambda_f$. For $V_{S(3)}$ (the +/0 transition), we obtain $\lambda_i = 0.67$ eV and $\lambda_f = 0.59$ eV, giving $\Delta E_{Stokes} = 1.26$ eV. For $Sb_{S(2)}$ (the 0/− transition), we obtain $\lambda_i = 0.32$ eV and $\lambda_f = 0.36$ eV, giving $\Delta E_{Stokes} = 0.68$ eV. These are now reported in the main text and in new Supplementary Note 4, where we also explain all of the parameters used in depth.

- Comparison with the measured photoluminescence red shift

The defect transition Stokes shift is the absorption emission separation within a specific defect charge transition. The experimental values discussed in the manuscript, about 0.1 eV in this work and about 0.6 eV in Yang et al., are PL red shifts relative to the optical gap, $E_g - E_{PL}$. To compare the calculated defect transitions with this experimental energy scale, the defect transition level E_t also needs to be included. For hole capture, $E_{PL} = E_t - \lambda_f$.

Using the calculated $E_g = 1.75$ eV and the calculated levels ($E_t = 0.59$ eV for $V_{S(3)}$, 0.61 eV for $Sb_{S(2)}$), the calculated hole capture emission energies are approximately 0 eV for $V_{S(3)}$ and 0.25 eV for $Sb_{S(2)}$. These correspond to red shifts of approximately 1.75 eV and 1.50 eV relative to the optical gap. These values are far larger than the 0.1 eV PL red shift measured here and 0.6 eV red shift reported by Yang et al.

- Interpretation

Both defects show large lattice relaxation and efficient multiphonon capture, consistent with their large calculated hole capture cross sections. The calculated configuration coordinate diagrams therefore support assigning $V_{S(3)}$ and $Sb_{S(2)}$ as deep nonradiative recombination centres. Any radiative signatures from these specific deep defect transitions would be expected to be weak or masked by the competing nonradiative capture pathway. We therefore do not assign the measured near band edge PL red shift to direct radiative emission from $V_{S(3)}$ or $Sb_{S(2)}$ based on these configuration coordinate diagrams.

We note that the defect transition Stokes shift of $Sb_{S(2)}$, 0.68 eV, is numerically close to the 0.6 eV PL red shift reported by Yang et al. However, these are different quantities: the former is the absorption emission separation within a deep defect transition, whereas the latter is a red shift

relative to the optical gap. Our analysis therefore does not require assignment of the 0.6 eV feature to either of these deep defects.

- Changes made

Main text (Page 20, highlighted in blue): we added a brief statement of the Franck–Condon relaxation energies and defect transition Stokes shifts for $V_{S(3)}$ and $Sb_{S(2)}$ with their implication for the assignment of the measured PL.

Supplementary Note 4: we added the full analysis, including the definitions of λ_i, λ_f, and ΔE_{Stokes} (Supplementary Fig. 18), together with tables (Supplementary Table 6 and 7) reporting the defect transition Stokes shifts and the defect-related emission energies.

Reviewer 3:

The authors have convincingly addressed my remaining concerns. I recommend the manuscript for publication.

Response: We are thrilled to see the reviewer recommend acceptance of our work, and would like to thank them for their time.

Reviewer 2:

The authors have addressed all my concerns, and I support the publication of this paper.

Response: We would like to thank the reviewer for their time and expertise, and are glad to see they recommend publication of our paper.
